# Etiology of *Cyclocarya paliurus* Anthracnose in Jiangsu Province, China

**DOI:** 10.3389/fpls.2020.613499

**Published:** 2021-01-18

**Authors:** Xiang-rong Zheng, Mao-jiao Zhang, Xu-lan Shang, Sheng-zuo Fang, Feng-mao Chen

**Affiliations:** Collaborative Innovation Center of Sustainable Forestry in Southern China, College of Forestry, Nanjing Forestry University, Nanjing, China

**Keywords:** *Cyclocarya paliurus*, etiology, fungicide sensitivity, *Colletotrichum*, anthracnose

## Abstract

*Cyclocarya paliurus* is an extremely valuable and multifunctional tree species whose leaves have traditionally been used in used in medicine or as a medicinal tea in China. In recent years, anthracnose has been frequently observed on young leaves of *C. paliurus* in several nurseries located in Jiangsu Province, resulting in great yield and quality losses. To date, no information is available about the prevalence of *C. paliurus* anthracnose in China. The main purpose of the present study was to characterize the etiology of *C. paliurus* anthracnose. Phylogenetic analysis of the eight-loci concatenated dataset revealed that all 44 single-spore *Colletotrichum* isolates belonged to three species in the *Colletotrichum gloeosporioides* species complex, namely, *Colletotrichum aenigma*, *Colletotrichum fructicola*, and *C. gloeosporioides* sensu stricto. Phenotypic features, including the colony appearance and the morphology of conidia, appressoria, and ascospores, were consistent with the phylogenetic grouping. Virulence tests validated that the three *Colletotrichum* species could cause typical symptoms of anthracnose on *C. paliurus* leaves, similar to those observed in the field. The optimum mycelial growth temperature ranged from 25 to 30°C for all representative isolates, while *C. gloeosporioides* s. s. isolates exhibited greater tolerance to high temperature (40°C). Fungicide sensitivity assays indicated that all three *Colletotrichum* species were sensitive to tetramycin, which may be a potential alternative for the management of *C. paliurus* anthracnose. To our knowledge, this study provides the first report of *C. aenigma*, *C. fructicola*, and *C. gloeosporioides* s. s. causing *C. paliurus* anthracnose in China as well as in the world.

## Introduction

Cultivated for fine timber and as a medicinal plant, *Cyclocarya paliurus* is the sole extant species in the genus *Cyclocarya* and is native to China, naturally distributed in mountainous regions in the middle and lower reaches of the Yangtze River (Fang et al., 2011; Deng et al., 2015; Xie et al., 2015; Zheng et al., 2020). In Chinese folklore, *C. paliurus* is commonly called the “sweet tea tree,” and its leaves have traditionally been used as drug formulations for the treatment of obesity or diabetes mellitus (Fang et al., 2011; Cao et al., 2017; Xie et al., 2018). In recent years, increasing attention has been paid to *C. paliurus* because phytochemical studies have demonstrated that the extracts of its leaves possess a wide range of biological activities beneficial to human beings, such as antihypertensive (Xie et al., 2006), hypoglycemic (Wang et al., 2013), anti-HIV-1 (Zhang et al., 2010), antioxidant (Xie et al., 2010; Wang et al., 2013; Liu et al., 2018a, b), antitumor (Liu et al., 2018b), and anticancer (Xie et al., 2013) activities. Current focal studies of *C. paliurus* have concentrated on producing or identifying the bioactive components in its leaves. Unfortunately, to date, no information about *C. paliurus* anthracnose is available, and this disease could become a limiting factor affecting the *C. paliurus* tea industry.

The Coelomycetous genus *Colletotrichum* Corda includes plant pathogens responsible for anthracnose diseases with a global distribution (Hyde et al., 2009; Wikee et al., 2011; Cannon et al., 2012; Dean et al., 2012; He et al., 2019). From the perspective of economic and scientific importance, *Colletotrichum* was denoted the eighth most significant fungal phytopathogen group worldwide (Dean et al., 2012), attacking over 3200 dicot and monocot plant species (Manire et al., 2002; O’Connell et al., 2012). The morphological taxonomy of *Colletotrichum* species has historically been arduous owing to overlapping characteristics, and the morphology sometimes varies with environmental factors (e.g., temperature, illumination, etc.) in culture (Freeman et al., 1998; Cannon et al., 2012; Damm et al., 2019). Molecular tools have been widely applied to effectively identify and define fungi at the species level. In recent years, the majority of studies regarding anthracnose were conducted principally via morphology and multigene phylogeny based on modern taxonomic concepts, which provides a more precise and robust solution (Damm et al., 2012; Liu et al., 2014, 2015; De Silva et al., 2017a; Diao et al., 2017; Guarnaccia et al., 2017; Fu et al., 2019).

*Colletotrichum* spp. infections initially occur via the attachment of spores to the host plant surface, followed by spore germination and the formation of an appressorium, which penetrates the plant cuticle. This process suggests that appressoria and spores play a critical role in the infection cycle and that certain highly inhibitory substances against spore germination and appressorium production would be potential alternatives to control anthracnose (De Silva et al., 2017b; Gao et al., 2020; Konsue et al., 2020). Currently, chemical pesticides are identified as the principal agents used for anthracnose management (Bi et al., 2011). However, excessive use of such chemicals has also brought a series of challenges over time (Lu et al., 2010; Hu et al., 2015; Duan et al., 2018), including pathogen resistance and residual toxicity that affects human health and the environment (Kim et al., 2015; Alijani et al., 2019). The selection of environmentally safe, high-efficacy and relatively new fungicides is therefore imperative.

The application of antibiotic fungicides derived from metabolites of beneficial microbes to control phytopathogens has recently attracted increased attention since these compounds have been found to be environmentally friendly and may help to overcome pesticide resistance due to their low toxicity to non-target organisms and structural versatility (Moreira and May De Mio, 2015; Simionato et al., 2017; Han et al., 2020), offering a safe and effective way to circumvent the drawbacks of chemically synthesized pesticides and decreasing the environmental risks associated with their contamination (Ma et al., 2018a; Zhu et al., 2018).

Tetramycin, the fermentation metabolite of *Streptomyces ahygroscopicus*, exhibits excellent inhibitory activity against many plant pathogens, including *Botrytis cinera*, *Passalora fulva*, *Phytophthora capsici*, and *Pyricularia oryzae* (Zhong et al., 2010; Ren et al., 2014; Song et al., 2016; Chen L. L. et al., 2017; Ma et al., 2018a), which has been registered to manage rice and fruit crop diseases in China (Zhao et al., 2010). On the other hand, a previous study reported that tetramycin has the potential to elicit disease resistance by activating plant defensive enzymes, including polyphenol oxidase (PPO), peroxidase (POD), and phenylalanine ammonia lyase (PAL) (Zhong et al., 2010). Owing to its environmental friendliness and high efficiency, tetramycin has become the preferred fungicide in recent years (Song et al., 2016; Ma et al., 2018a, b).

Phenazine-1-carboxylic acid (PCA) is an important N-containing heterocyclic secondary metabolite (Zhu et al., 2019), which has been proved having antimicrobial (Palchykovska et al., 2012; Udumula et al., 2017), antitumorigenic (Gupta et al., 2014), antiviral, and antitubercular effects (Logua et al., 2009; Palchykovska et al., 2012), widely existed in microbial metabolites of *Pseudomonads* and *Streptomycetes* (Zhu et al., 2019). Particularly, in recent years, PCA received much attention due to outstanding inhibition effects against several phytopathogenic fungi in agricultural application (Zhu et al., 2019; Han et al., 2020). In China, PCA has been registered as the biofungicide “Shenqinbactin” for its environmental friendliness, low toxicity to human and animals, and the enhancement of crop production (Zhu et al., 2018, 2019; Han et al., 2020).

Kasugamycin, the fermentation product of *Streptomyces kasugaensis*, is a member of the aminoglycoside antibiotic (Uppala and Zhou, 2018). It was originally developed as a biofungicide for the management of rice blast caused by *P. oryzae*. Kasugamycin inhibits protein biosynthesis, with both fungicidal and bactericidal activities (McGhee and Sundin, 2011). Due to it is high efficiency and friendliness to environment, the use of Kasugamycin in United States has been approved by EPA for controlling diseases of several pome fruits in the past decade^1^.

**FIGURE 1:**
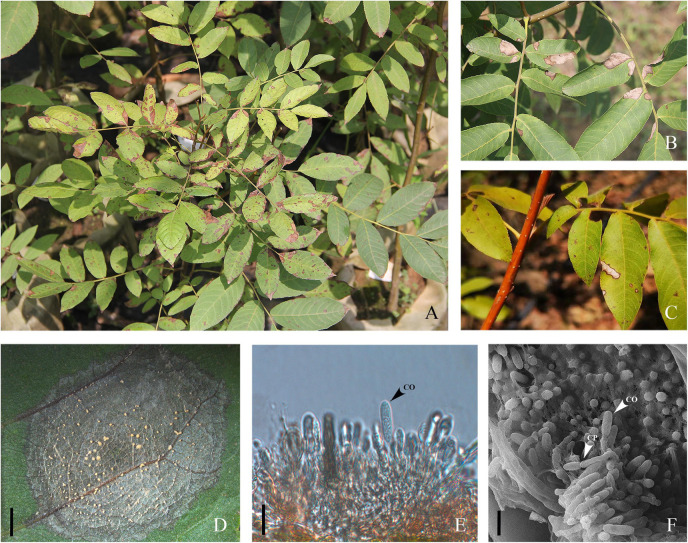
*Cyclocarya paliurus* leaves with typical necrosis symptoms of anthracnose. **(A)** Diseased leaves in the field. **(B,C)** Initial and later symptoms of *Cyclocarya paliurus* anthracnose. **(D)** Gelatinous orange spore masses oozed and arranged in concentric rings; bar = 2 mm. **(E)** Longitudinal section of acervuli developed on leaf lesion; co indicates conidia; bar = 10 μm. **(F)** Scanning electron photomicrograph of acervuli formed on leaf lesion; co and cp indicate conidia and conidiophores, respectively; bar = 10 μm.

In 2018, during an investigation of *C. paliurus*, serious anthracnose symptoms (Figure 1A) were observed in several nurseries located in the scientific research base of Nanjing Forestry University in Baima town (Baima), Nanjing. Over a half of the leaves were infected in Baima based on our observation. This anthracnose has been considered an emerging disease, but it is becoming endemic; nevertheless, the etiology, epidemiology, and management of this disease are uncertain. Hence, the objectives of the present study were to (1) accurately identify the *Colletotrichum* spp. causing *C. paliurus* anthracnose in Jiangsu Province, China, combining morphological and biological characteristics with molecular phylogenetic analyses; (2) examine the virulence of these fungi on *C. paliurus* leaves *in vitro*; and (3) characterize and compare the inhibitory effects of biofungicides against different *Colletotrichum* spp. *in vitro*.

## Materials and Methods

### Field Survey and Sampling

A field survey of *C. paliurus* anthracnose was carried out in Nanjing (five nurseries), Changzhou (four nurseries), and Yancheng (four nurseries) in September and October 2018 during the late growing season. Disease incidence was calculated as the percentage of trees displaying anthracnose symptoms out of the total number of evaluated trees (Bautista-Cruz et al., 2019). Three leaves exhibiting typical symptoms of anthracnose were randomly sampled per plant, and at least 10 symptomatic plants were sampled per nursery. All samples were then packaged in self-sealing bags and transported in an ice chest to the laboratory and then stored at 5°C prior to isolation.

### Colletotrichum Isolation

To isolate the fungus, small sections (4-by-4 mm pieces) were removed from the margin of leaf lesions, surface disinfected in 1% (vol/vol) NaClO_3_ for 45 s and 75% ethanol for 30 s, rinsed in sterile distilled water three times, and air-dried on sterilized paper. The sections were then cultured onto 2% potato dextrose agar (PDA) (five sections per plate) amended with 100 μg/mL ampicillin to inhibit bacterial growth and incubated at 25°C in the dark. The emerging edges of the fungal mycelium were observed daily and transferred aseptically onto new PDA plates. Colonies similar in morphology to *Colletotrichum* spp. were purified using the monosporic isolation procedure described by Cai et al. (2009), and single-spore cultures were preserved in PDA slant test tubes at 4°C for follow-up studies. All isolates used in this study were deposit in State Key Laboratory of Forest Protection in Nanjing Forestry University.

### Molecular Identification and Phylogenetic Analysis

For further characterization of the *Colletotrichum* spp., total genomic DNA (gDNA) of all single-spore isolates was extracted following the CTAB method described by Than et al. (2008). The concentrations of gDNA extracts were adjusted to 100 ng/μL with autoclaved double distilled water (ddH_2_O) using a NanoDrop 2000 spectrophotometer (Thermo Fisher Scientific, Madison, WI, United States) and stored at −20°C before use. Polymerase chain reaction (PCR) amplification was performed for the following loci: the ITS region, calmodulin (CAL), β-tubulin (TUB), actin (ACT), chitin synthase 1 (CHS-1), glyceraldehyde-3-phosphate dehydrogenase (GAPDH), glutamine synthetase (GS), and Apn2-Mat1-2 intergenic spacer (ApMat) genes. PCR amplifications were conducted in a 25 μL volume, mixed with 8.5 μL of ddH_2_O, 1 μL of each primer (10 μM), 2 μL of template DNA, and 12.5 μL of 2 × PCR Taq Master Mix (Applied Biological Materials Inc., Canada), using an Eppendorf Nexus Thermal Cycler (Germany). A negative control was added in all amplifications, where an equal volume of ddH_2_O replaced the template DNA. The primers and PCR settings for each locus are shown in Table 1. Amplification products were purified and sequenced by Jie Li Biotech Company (Shanghai, China). Forward and reverse DNA sequences were assembled and manually edited where necessary using Bioedit software (version 7.0.5^2^), and the consensus sequences were deposited in GenBank (Table 2). Reference sequences from ex-type or other authoritative specimens of *Colletotrichum* spp. were retrieved from GenBank and aligned with sequences generated herein for constructing phylogenetic trees, with *C. boninense* (MAFF 305972) used as an outgroup (Tables 2, 3).

**TABLE 1:** Descriptions and sequence accession numbers obtained from GenBank of the *Colletotrichum* spp. used in the phylogenetic study.

Species	Culture/Isolate^a^	Host	City/Country	GenBank accession number^b^							
				ITS	GAPDH	CAL	ACT	CHS-1	TUB	GS	ApMat
*C. aenigma*	**ICMP 18608**	*Persea americana*	Israel	JX010244	JX010044	JX009683	JX009443	JX009774	JX010389	JX010078	KM360143
	HC3^c^	*Cyclocarya paliurus*	Changzhou, China	*MT476807*	*MT501007*	*MT500919*	*MT500875*	*MT500963*	*MT501051*	*MW344671*	*MW344720*
	JS2	*C. paliurus*	Changzhou, China	*MT476808*	*MT501008*	*MT500920*	*MT500876*	*MT500964*	*MT501052*	*MW344672*	*MW344721*
	JS7	*C. paliurus*	Changzhou, China	*MT476809*	*MT501009*	*MT500921*	*MT500877*	*MT500965*	*MT501053*	*MW344673*	*MW344722*
	SC7^c^	*C. paliurus*	Nanjing, China	*MT476810*	*MT501010*	*MT500922*	*MT500878*	*MT500966*	*MT501054*	*MW344674*	*MW344723*
	YM8^c^	*C. paliurus*	Yancheng, China	*MT476811*	*MT501011*	*MT500923*	*MT500879*	*MT500967*	*MT501055*	*MW344675*	*MW344724*
	ZH2	*C. paliurus*	Yancheng, China	*MT476812*	*MT501012*	*MT500924*	*MT500880*	*MT500968*	*MT501056*	*MW344676*	*MW344725*
*C. aeschynomenes*	**ICMP 17673**	*Aeschynomene virginica*	United States	JX010176	JX009930	JX009721	JX009483	JX009799	JX010392	JX010081	KM360145
*C. alatae*	**CBS 304.67**, ICMP 17919	*Dioscorea alata*	India	JX010190	JX009990	JX009738	JX009471	JX009837	JX010383	JX010065	KC888932
*C. alienum*	**ICMP 12071**	*Malus domestica*	New Zealand	JX010251	JX010028	JX009654	JX009572	JX009882	JX010411	JX010101	KM360144
*C. aotearoa*	**ICMP 18537**	*Coprosma* sp.	New Zealand	JX010205	JX010005	JX009611	JX009564	JX009853	JX010420	JX010113	KC888930
*C. arecicola*	CGMCC 3.19667, **HNBL5**	*Areca catechu*	Wenchang, China	MK914635	MK935455		MK935374	MK935541	MK935498		MK935413
*C. asianum*	**ICMP 18580**, CBS 130418	*Coffea arabica*	Thailand	FJ972612	JX010053	FJ917506	JX009584	JX009867	JX010406	JX010096	FR718814
*C. boninense*	**MAFF 305972**	*Crinum asiaticum var. sinicum*	Japan	JX010292	JX009905	JQ005674	JX009583	JX009827	JQ005588		
*C. clidemiae*	**ICMP 18658**	*Clidemia hirta*	United States, Hawaii	JX010265	JX009989	JX009645	JX009537	JX009877	JX010438	JX010129	KC888929
*C. cordylinicola*	**MFLUCC 090551**, ICMP 18579	*Cordyline fruticosa*	Thailand	JX010226	JX009975	HM470238	HM470235	JX009864	JX010440	JX010122	JQ899274
*C. fructicola*	**ICMP 18581**, CBS 130416	*Coffea arabica*	Thailand	JX010165	JX010033	FJ917508	FJ907426	JX009866	JX010405	JX010095	JQ807838
	BM5^c^	*C. paliurus*	Nanjing, China	*MT476813*	*MT501013*	*MT500925*	*MT500881*	*MT500969*	*MT501057*	*MW344677*	*MW344726*
	BX1	*C. paliurus*	Nanjing, China	*MT476814*	*MT501014*	*MT500926*	*MT500882*	*MT500970*	*MT501058*	*MW344678*	*MW344727*
	F5	*C. paliurus*	Changzhou, China	*MT476815*	*MT501015*	*MT500927*	*MT500883*	*MT500971*	*MT501059*	*MW344679*	*MW344728*
	GX1^c^	*C. paliurus*	Changzhou, China	*MT476816*	*MT501016*	*MT500928*	*MT500884*	*MT500972*	*MT501060*	*MW344680*	*MW344729*
	GT7	*C. paliurus*	Changzhou, China	*MT476817*	*MT501017*	*MT500929*	*MT500885*	*MT500973*	*MT501061*	*MW344681*	*MW344730*
	HC2^c^	*C. paliurus*	Changzhou, China	*MT476818*	*MT501018*	*MT500930*	*MT500886*	*MT500974*	*MT501062*	*MW344682*	*MW344731*
	HC6	*C. paliurus*	Changzhou, China	*MT476819*	*MT501019*	*MT500931*	*MT500887*	*MT500975*	*MT501063*	*MW344683*	*MW344732*
	JS3	*C. paliurus*	Changzhou, China	*MT476820*	*MT501020*	*MT500932*	*MT500888*	*MT500976*	*MT501064*	*MW344684*	*MW344733*
	JS9	*C. paliurus*	Changzhou, China	*MT476821*	*MT501021*	*MT500933*	*MT500889*	*MT500977*	*MT501065*	*MW344685*	*MW344734*
	LC7^c^	*C. paliurus*	Nanjing, China	*MT476822*	*MT501022*	*MT500934*	*MT500890*	*MT500978*	*MT501066*	*MW344686*	*MW344735*
	LG2	*C. paliurus*	Nanjing, China	*MT476823*	*MT501023*	*MT500935*	*MT500891*	*MT500979*	*MT501067*	*MW344687*	*MW344736*
	LG4	*C. paliurus*	Nanjing, China	*MT476824*	*MT501024*	*MT500936*	*MT500892*	*MT500980*	*MT501068*	*MW344688*	*MW344737*
	LV2	*C. paliurus*	Nanjing, China	*MT476825*	*MT501025*	*MT500937*	*MT500893*	*MT500981*	*MT501069*	*MW344689*	*MW344738*
	NC25^c^	*C. paliurus*	Nanjing, China	*MT476826*	*MT501026*	*MT500938*	*MT500894*	*MT500982*	*MT501070*	*MW344690*	*MW344739*
	NC26	*C. paliurus*	Nanjing, China	*MT476827*	*MT501027*	*MT500939*	*MT500895*	*MT500983*	*MT501071*	*MW344691*	*MW344740*
	PL2	*C. paliurus*	Changzhou, China	*MT476828*	*MT501028*	*MT500940*	*MT500896*	*MT500984*	*MT501072*	*MW344692*	*MW344741*
	PX3	*C. paliurus*	Changzhou, China	*MT476829*	*MT501029*	*MT500941*	*MT500897*	*MT500985*	*MT501073*	*MW344693*	*MW344742*
	SC6^c^	*C. paliurus*	Nanjing, China	*MT476830*	*MT501030*	*MT500942*	*MT500898*	*MT500986*	*MT501074*	*MW344694*	*MW344743*
	SC9	*C. paliurus*	Nanjing, China	*MT476831*	*MT501031*	*MT500943*	*MT500899*	*MT500987*	*MT501075*	*MW344695*	*MW344744*
	T5	*C. paliurus*	Nanjing, China	*MT476832*	*MT501032*	*MT500944*	*MT500900*	*MT500988*	*MT501076*	*MW344696*	*MW344745*
	T9	*C. paliurus*	Nanjing, China	*MT476833*	*MT501033*	*MT500945*	*MT500901*	*MT500989*	*MT501077*	*MW344697*	*MW344746*
	H3	*C. paliurus*	Nanjing, China	*MT476834*	*MT501034*	*MT500946*	*MT500902*	*MT500990*	*MT501078*	*MW344698*	*MW344747*
	H4	*C. paliurus*	Nanjing, China	*MT476835*	*MT501035*	*MT500947*	*MT500903*	*MT500991*	*MT501079*	*MW344699*	*MW344748*
	YH6^c^	*C. paliurus*	Yancheng, China	*MT476836*	*MT501036*	*MT500948*	*MT500904*	*MT500992*	*MT501080*	*MW344700*	*MW344749*
	YH7	*C. paliurus*	Yancheng, China	*MT476837*	*MT501037*	*MT500949*	*MT500905*	*MT500993*	*MT501081*	*MW344701*	*MW344750*
	YM2	*C. paliurus*	Yancheng, China	*MT476838*	*MT501038*	*MT500950*	*MT500906*	*MT500994*	*MT501082*	*MW344702*	*MW344751*
	YM7	*C. paliurus*	Yancheng, China	*MT476839*	*MT501039*	*MT500951*	*MT500907*	*MT500995*	*MT501083*	*MW344703*	*MW344752*
	ZH6	*C. paliurus*	Yancheng, China	*MT476840*	*MT501040*	*MT500952*	*MT500908*	*MT500996*	*MT501084*	*MW344704*	*MW344753*
*C. gloeosporioides*	IMI 356878, **ICMP 17821,** CBS 112999	*Citrus sinensis*	Italy	JX010152	JX010056	JX009731	JX009531	JX009818	JX010445	JX010085	JQ807843
	BM6^c^	*C. paliurus*	Nanjing, China	*MT476841*	*MT501041*	*MT500953*	*MT500909*	*MT500997*	*MT501085*	*MW344705*	*MW344754*
	F8	*C. paliurus*	Changzhou, China	*MT476842*	*MT501042*	*MT500954*	*MT500910*	*MT500998*	*MT501086*	*MW344706*	*MW344755*
	GX3^c^	*C. paliurus*	Changzhou, China	*MT476843*	*MT501043*	*MT500955*	*MT500911*	*MT500999*	*MT501087*	*MW344707*	*MW344756*
	JS1	*C. paliurus*	Changzhou, China	*MT476844*	*MT501044*	*MT500956*	*MT500912*	*MT501000*	*MT501088*	*MW344708*	*MW344757*
	JS5	*C. paliurus*	Changzhou, China	*MT476845*	*MT501045*	*MT500957*	*MT500913*	*MT501001*	*MT501089*	*MW344709*	*MW344758*
	LC2^c^	*C. paliurus*	Nanjing, China	*MT476846*	*MT501046*	*MT500958*	*MT500914*	*MT501002*	*MT501090*	*MW344710*	*MW344759*
	LC6	*C. paliurus*	Nanjing, China	*MT476847*	*MT501047*	*MT500959*	*MT500915*	*MT501003*	*MT501091*	*MW344711*	*MW344760*
	YM4^c^	*C. paliurus*	Yancheng, China	*MT476848*	*MT501048*	*MT500960*	*MT500916*	*MT501004*	*MT501092*	*MW344712*	*MW344761*
	YM5	*C. paliurus*	Yancheng, China	*MT476849*	*MT501049*	*MT500961*	*MT500917*	*MT501005*	*MT501093*	*MW344713*	*MW344762*
	ZH3	*C. paliurus*	Yancheng, China	*MT476850*	*MT501050*	*MT500962*	*MT500918*	*MT501006*	*MT501094*	*MW344714*	*MW344763*
*C. horii*	**NBRC 7478**, ICMP 10492	*Diospyros kaki*	Japan	*GQ329690*	*GQ329681*	*JX009604*	*JX009438*	*JX009752*	*JX010450*	JX010137	JQ807840
*C. kahawae* subsp. *ciggaro*	**ICMP 18539**	*Olea europaea*	Australia	JX010230	JX009966	JX009635	JX009523	JX009800	JX010434	JX010132	
*C. kahawae* subsp. *kahawae*	IMI 319418, **ICMP 17816**	*Coffea arabica*	Kenya	JX010231	JX010012	JX009642	JX009452	JX009813	JX010444	JX010130	JQ894579
*C. ledongense*	LD1683, **CGMCC 3.18888**	*Hevea brasiliensis*	Hainan, China	MG242009	MG242017	MG242013	MG242015	MG242019	MG242011	MG242021	
*C. musae*	**CBS 116870**, ICMP 19119	*Musa* sp.	United States	JX010146	JX010050	JX009742	JX009433	JX009896	HQ596280	JX010103	KC888926
*C. noveboracense*	**AFKH109**, CBS 146410	*Malus domestica*	United States	MN646685	MN640567	MN640566	MN640565		MN640569	MN640568	MN640564
*C. nupharicola*	**ICMP 18187,** CBS:470.96	*Nuphar lutea subsp. polysepala*	United States	JX010187	JX009972	JX009663	JX009437	JX009835	JX010398	JX010088	JX145319
*C. perseae*	**GA100, CBS 141365**	*Persea americana*	Israel	KX620308	KX620242	KX620206	KX620145		KX620341	KX620275	KX620177
*C. psidii*	**CBS 145.29,** ICMP 19120	*Psidium* sp.	Italy	JX010219	JX009967	JX009743	JX009515	JX009901	JX010443	JX010133	KC888931
*C. queenslandicum*	**ICMP 1778**	*Carica papaya*	Australia	JX010276	JX009934	JX009691	JX009447	JX009899	JX010414	JX010104	KC888928
*C. salsolae*	**ICMP 19051**	*Salsola tragus*	Hungary	JX010242	JX009916	JX009696	JX009562	JX009863	JX010403	JX010093	KC888925
*C. siamense*	**ICMP 18578**, CBS 130417	*Coffea arabica*	Thailand	JX010171	JX009924	FJ917505	FJ907423	JX009865	JX010404	JX010094	JQ899289
*C. siamense* (syn. *C. hymenocallidis*)	**CBS 125378**, ICMP 18642	*Hymenocallis americana*	China	JX010278	JX010019	JX009709	GQ856775	GQ856730	JX010410	JX010100	JQ899283
*C. theobromicola*	**CBS 124945**, ICMP 18649	*Theobroma cacao*	Panama	JX010294	JX010006	JX009591	JX009444	JX009869	JX010447	JX010139	KC790726
*C. ti*	**ICMP 4832**	*Cordyline* sp.	New Zealand	JX010269	JX009952	JX009649	JX009520	JX009898	JX010442	JX010123	KM360146
*C. tropicale*	**CBS 124949**, ICMP 18653	*Theobroma cacao*	Panama	JX010264	JX010007	JX009719	JX009489	JX009870	JX010407	JX010097	KC790728
*C. xanthorrhoeae*	**BRIP 45094**, ICMP 17903, CBS 127831	*Xanthorrhoea preissii*	Australia	JX010261	JX009927	JX009653	JX009478	JX009823	JX010448	JX010138	KC790689

*^a^Culture numbers in bold type represent ex-type or other authentic specimens. BRIP, Plant Pathology Herbarium, Department of Employment, Economic, Development and Innovation, Queensland, Australia; CBS, Culture collection of the Centraalbureau voor Schimmelcultures, Fungal Biodiversity Centre, Utrecht, Netherlands; ICMP, International Collection of Microorganisms from Plants, Auckland, New Zealand; IMI, Culture collection of CABI Europe UK Centre, Egham, United Kingdom; MAFF, MAFF Genebank Project, Ministry of Agriculture, Forestry and Fisheries, Tsukuba, Japan; MFLUCC, Mae Fah Luang University Culture Collection, ChiangRai, Thailand; NBRC, NITE Biological Resource Centre, Japan. MAFF 305972 (C. boninense) was added as an outgroup. ^b^Sequences in italics were generated in this study. ITS, internal transcribed spacers 1 and 2 together with 5.8S nrDNA; GAPDH, partial glyceraldehyde-3-phosphate dehydrogenase gene; CAL, partial calmodulin gene; ACT, partial actin gene; CHS-1, partial chitin synthase 1 gene; TUB2, partial beta-tubulin gene. ^c^Isolates used for morphological and biological analysis, virulence tests, and biofungicide sensitivity assays.*

**TABLE 2:** Primers used in this study, with sequences, conditions and sources.

Gene	Product name	Primer	Direction	Sequence (5′-3′)	PCR conditions	References
ITS	Internal transcribed spacer	ITS1	Forward	CTTGGTCATTTAGAGGAAGTAA	Denaturation for 4 min at 94°C, followed by 30 cycles; 30 s at 94°C, 30 s at 55°C, 30 s at 72°C, and a final extension of 10 min at 72°C	Gardes and Bruns, 1993
		ITS4	Reverse	TCCTCCGCTTATTGATATGC		White et al., 1990
GAPDH	Glyceraldehyde-3-phosphate dehydrogenase	GDF1	Forward	GCCGTCAACGACCCCTTCATTGA	Denaturation for 4 min at 94°C, followed by 30 cycles; 30 s at 94°C, 30 s at 60°C, 30 s at 72°C, and a final extension of 10 min at 72°C	Guerber et al., 2003
		GDR1	Reverse	GGGTGGAGTCGTACTTGAGCATGT		Guerber et al., 2003
ACT	Actin	ACT-512F	Forward	ATGTGCAAGGCCGGTTTCGC	Denaturation for 4 min at 94°C, followed by 30 cycles; 30 s at 94°C, 30 s at 57°C, 30 s at 72°C, and a final extension of 10 min at 72°C	Carbone and Kohn, 1999
		ACT-783R	Reverse	TACGAGTCCTTCTGGCCCAT		Carbone and Kohn, 1999
TUB	β-tubulin	T1	Forward	AACATGCGTGAGATTGTAAGT	Denaturation for 4 min at 94°C, followed by 30 cycles; 30 s at 94°C, 30 s at 61°C, 30 s at 72°C, and a final extension of 10 min at 72°C	O’Donnell and Cigelnik, 1997
		Bt-2b	Reverse	ACCCTCAGTGTAGTGACCCTTGGC		Glass and Donaldson, 1995
CAL	Calmodulin	CL1A	Forward	GATCAAGGAGGCCTTCTC	Denaturation for 4 min at 94°C, followed by 30 cycles; 30 s at 94°C, 30 s at 58°C, 30 s at 72°C, and a final extension of 10 min at 72°C	O’Donnell et al., 2000
		CL2A	Reverse	TTTTTGCATCATGAGTTGGAC		O’Donnell et al., 2000
CHS-1	Chitin synthase 1	CHS-79F	Forward	TGGGGCAAGGATGCTTGGAAGAAG	Denaturation for 4 min at 94°C, followed by 30 cycles; 30 s at 94°C, 30 s at 58°C, 30 s at 72°C, and a final extension of 10 min at 72°C	Carbone and Kohn, 1999
		CHS-354R	Reverse	TGGAAGAACCATCTGTGAGAGTTG		Carbone and Kohn, 1999
GS	Glutamine synthetase	GSLF2	Forward	TACACGAGSAAAAGGATACGC	Denaturation for 4 min at 94°C, followed by 30 cycles; 30 s at 94°C, 30 s at 54°C, 30 s at 72°C, and a final extension of 10 min at 72°C	Liu et al., 2016
		GSLR1	Reverse	AGRCGCACATTGTCAGTATCG		Liu et al., 2016
ApMat	Apn2-Mat1-2	AM-F	Forward	TCATTCTACGTATGTGCCCG	Denaturation for 3 min at 94°C, followed by 30 cycles; 45 s at 94°C, 45 s at 62°C, 1 min at 72°C, and a final extension of 7 min at 72°C	Silva et al., 2012
		AM-R	Reverse	CCAGAAATACACCGAACTTGC		Silva et al., 2012

**TABLE 3:** Morphological characteristics of *Colletotrichum* isolates from *Cyclocarya paliurus*.

Species/Isolate	Conidia		Appressoria		Ascospore		
	Length (μm)	Width (μm)	Length (μm)	Width (μm)	Length (μm)	Width (μm)	Shape
***C. aenigma***							
HC3	18.58 ± 0.51ab (14.79–26.09)	7.44 ± 0.17b (5.56–10.2)	10.71 ± 0.25ab (8.35–13.68)	7.48 ± 0.18f (5.85–9.56)	18.4 ± 0.3bc (16.04–22.36)	7.32 ± 0.14a (6.11–9.06)	Cylindrical
SC7	18.82 ± 0.45ab (15.74–26.64)	8.17 ± 0.23a (5.73–11.13)	11.46 ± 0.42a (8.61–16.92)	7.96 ± 0.25abcde (5.87–11.73)	18.7 ± 0.24abc (16.91–21.94)	7.44 ± 0.14a (6.01–8.85)	Cylindrical
YM8	19.29 ± 0.43a (14.3–24.64)	8.29 ± 0.17a (6.44–10.97)	11.2 ± 0.24ab (9.6–14.34)	7.53 ± 0.14ab (5.87–9.06)	17.77 ± 0.16c (15.94–19.52)	6.75 ± 0.15b (5.56–9.17)	Cylindrical
***C. fructicola***							
BM5	16.24 ± 0.24d (12–18.68)	6.52 ± 0.17d (4.79–8.28)	10.7 ± 0.21ab (8.35–13.84)	7.79 ± 0.12bcdef (6.45–9.4)	19.55 ± 0.33a (16.67–23.75)	4.63 ± 0.08c (3.85–5.44)	Curved fusoid
GX1	16.52 ± 0.38d (13.64–23.14)	6.9 ± 0.19bcd (5.1–9.79)	10.75 ± 0.25ab (8.67–14.15)	7.88 ± 0.13bcdef (6.53–9.56)	19.3 ± 0.38ab (15.41–25.35)	4.68 ± 0.11c (3.59–5.81)	Curved fusoid
HC2	17.11 ± 0.42cd (12.39–22.34)	7.35 ± 0.16b (5.63–9.59)	10.87 ± 0.23ab (9.02–13.49)	7.83 ± 0.13bcdef (6.56–9.25)	18.82 ± 0.36ab (13.23–21.34)	4.72 ± 0.12c (3.4–5.84)	Curved fusoid
LC7	17.07 ± 0.24cd (14.44–19.92)	7.03 ± 0.17bcd (5.47–8.77)	10.97 ± 0.2ab (9.24–12.94)	7.69 ± 0.14def (6.55–8.97)	19.2 ± 0.34ab (14.32–24.04)	4.67 ± 0.11c (3.79–6.13)	Curved fusoid
NC25	16.38 ± 0.4d (12.89–22.79)	6.72 ± 0.18d (4.89–8.66)	11.17 ± 0.19ab (9.33–12.91)	7.77 ± 0.14cdef (6.54–9.26)	19.05 ± 0.37ab (16.53–23.89)	4.55 ± 0.09c (3.2–5.65)	Curved fusoid
SC6	16.68 ± 0.29cd (13.49–20.71)	6.58 ± 0.16d (4.5–8.25)	10.42 ± 0.23b (8.49–12.86)	7.86 ± 0.11bcdef (6.61–8.95)	19.06 ± 0.39ab (13.64–24.69)	4.68 ± 0.12c (3.1–6.19)	Curved fusoid
YH6	16.61 ± 0.38cd (14.36–22.58)	6.68 ± 0.17d (4.95–8.83)	10.68 ± 0.25ab (8.04–12.95)	7.99 ± 0.14abcde (6.76–9.97)	18.8 ± 0.31ab (15.41–21.99)	4.72 ± 0.12c (3.7–6.13)	Curved fusoid
***C. gloeosporioides***							
BM6	17.07 ± 0.28cd (14.72–19.84)	6.76 ± 0.16cd (5.15–8.45)	11.05 ± 0.2ab (9.55–13.48)	8.27 ± 0.15ab (6.94–10.39)	13.24 ± 0.3d (10.66–16.8)	4.83 ± 0.09c (3.91–5.98)	Cylindrical
GX3	17.25 ± 0.37cd (13.44–23.81)	6.93 ± 0.12bcd (5.87–8.1)	11.12 ± 0.18ab (9.13–13.01)	8.1 ± 0.16abcd (6.06–10.61)	13.14 ± 0.32d (9.86–17.07)	4.81 ± 0.09c (3.62–5.84)	Cylindrical
LC2	16.96 ± 0.26cd (15.06–21.22)	7.04 ± 0.16bcd (4.95–9.69)	11.18 ± 0.18ab (9.48–13.12)	8.24 ± 0.14abc (6.94–10.11)	12.9 ± 0.33d (9.02–16.54)	4.89 ± 0.09c (3.79–5.84)	Cylindrical
YM4	17.76 ± 0.33bc (15.69–24.82)	7.26 ± 0.13bc (5.96–8.67)	11.1 ± 0.21ab (9.63–13.91)	8.38 ± 0.12a (6.99–10.16)	13.23 ± 0.29d (10.73–16.06)	4.85 ± 0.08c (4.19–5.98)	Cylindrical

*Data are mean ± standard error, with ranges in parentheses. Columns with the same letter do not differ significantly according to Duncan’s test (P < 0.05).*

The phylogenetic analysis for each individual locus and the concatenated matrix were inferred under the Bayesian inference (BI) and maximum-likelihood (ML) criteria in MrBayes 3.2.6 (Ronquist et al., 2012) and MEGA 7 (Kumar et al., 2016), respectively. For BI analysis, the best nucleotide substitution model of each locus was ascertained by MrModeltest 2.3 according to AICc, with K2 + I identified for CHS, TN93 identified for GADPH, K2 + G identified for ACT and ApMat, GTR + G identified for CAL and GS, and TN93 + G identified for ITS and TUB. Four Markov chains were run for 30 million generations simultaneously, with trees sampled every 1000 generations. The first 25% of trees were discarded as the burn-in phase of the analyses, while the remaining trees were used for calculating posterior probabilities (PPs) in the majority rule consensus tree. ML analysis was performed based on the GTR + G + I model, and clade support was determined by 1000 bootstrap replicates, with gaps treated as missing data.

### Morphological and Biological Characterization

Fourteen representative isolates were selected for further studies according to BI/ML phylogenetic analysis (Table 1). Mycelial blocks (2 mm in side length) aseptically taken from actively growing cultures were transferred to new PDA plates and incubated at 25°C in darkness. Colony characteristics, including conidiomata or ascomata production, were determined up to 30 days post-inoculation (dpi). Conidia, appressoria, ascospores, and asci for microscopy were obtained and examined according to the procedure described by Weir et al. (2012). At least 30 measurements per structure were recorded at ×100 magnification using a ZEISS Axio Imager A2m microscope (Carl Zeiss, Göttingen, Germany) equipped with differential interference contrast (DIC) optics. To observe fungal structures developed on infected tissue, leaves showing typical symptoms of anthracnose were collected and prepared using the method of Huang et al. (2018), with photomicrographs taken by a Regulus 8100 field emission scanning electron microscope (FE-SEM, Japan).

To determine the optimal temperature for colony growth, mycelial blocks (2 mm in side length) of 14 representative isolates were cultured as described above and incubated at temperatures of 5–40°C with 5°C intervals. The colony diameter was measured at two perpendicular angles, and the average was taken at 4 dpi. Five replicates per isolate were examined at all eight temperatures, and the experiment was conducted twice. Differences in the morphological and biological characteristics of the isolates were determined by one-way analysis of variance (ANOVA) using IBM SPSS Statistics 24.0 software (SPSS, Inc., Chicago, IL, United States).

### Virulence Tests of *Colletotrichum* Isolates

Virulence tests were conducted with reference to previous reports with minor modifications (Huang et al., 2016; Chen Y. et al., 2017; Xue et al., 2019). Fourteen representative *Colletotrichum* isolates were selected and cultured on PDA and used for virulence tests on detached *C. paliurus* leaves under controlled conditions (Table 2). Conidial suspensions of each isolate were prepared as previously described and adjusted to two concentrations of 1 × 10^6^ and 1 × 10^8^ conidia/mL with ddH_2_O.

Asymptomatic *C. paliurus* leaves were surface disinfected and air-dried as mentioned above, and then one piercing wound was made on the right side of each leaf using a sterile needle (insect pin, 0.71 mm in diameter), or the leaves were left unwounded. Wound inoculation was performed by placing an 8 μL conidial suspension (1 × 10^6^ conidia/mL) or mycelial blocks (5 mm in length) from margins of actively growing colonies onto each stab wound. Non-wound inoculation was conducted by placing an 8 μL spore suspension (1 × 10^8^ conidia/mL) or mycelial blocks onto the mid-right region of the leaves without pin pricking. Leaves inoculated with ddH_2_O or non-colonized PDA blocks were treated as negative controls. The experiment was conducted in triplicate for each treatment and control, involving five leaves per replicate. All treatments and controls were placed into transparent containers (334 × 215 × 87 mm) lined with moist sterile filter paper and sealed by plastic wrap to maintain a high relative humidity and then incubated at 25°C under a 12 h photoperiod in a growth chamber. The whole experiment was carried out twice.

Disease incidence was determined at 10 dpi, while the incubated leaves were monitored for the onset of anthracnose lesions for up to 20 dpi. Virulence was determined by measuring the diameter of the necrotic lesions in two perpendicular directions at 7 and 10 dpi for the wounded and non-wounded leaves, respectively. Differences in the virulence of the isolates were determined by ANOVA, and mean values were compared by Tukey’s test (*P* < 0.05) using SPSS as previously described. Each *Colletotrichum* isolate involved in the virulence test was reisolated from the inoculated leaves, and their identity was confirmed by morphological and molecular approaches as previously described to fulfill Koch’s postulates.

### Biofungicide Sensitivity Assessments *in vitro*

#### Effects on Mycelial Growth

Phenazine-1-carboxylic acid [1% active ingredient (a.i.); Shanghai Non-gle Biological Products Co., Ltd., Shanghai, China], tetramycin (0.3% a.i.; Liaoning Wkioc Bioengineering Co., Ltd., Liaoning, China), and kasugamycin (4% a.i.; Shaanxi Microbe Biotechnology Co., Ltd., Shaanxi, China) were used. Fourteen representative isolates were selected based on the above studies. The fungicide sensitivity of each isolate was tested on complete medium (CM) plates (Yeast extract 10 g/L, Casamino-acid 5 g/L, Agar 15 g/L, 1% sterile glucose after autoclaving) amended with fungicides. Mycelial blocks (2 mm in side length) aseptically taken from actively growing cultures were placed onto CM with or without (control) fungicide amendments. The final concentrations of each a.i. in the amended media were 0.1, 0.25, 0.5, 1, 2.5, and 5 μg/mL for tetramycin and 1, 2.5, 5, 10, 25, and 50 μg/mL for PCA and kasugamycin. Each treatment was tested in triplicate, and the entire experiment was repeated twice. The mean colony diameter was measured at 4 dpi, and the formula for percent inhibition was [(radial growth of the control – radial growth at fungicide concentration)/radial growth of the control] × 100%. Half of the maximal effective concentration (EC_50_) was estimated by regression to the log_10_ probability conversion of the percentage of inhibition of the fungicide concentrations.

#### Effects on Spore Germination

Tetramycin was selected to test its ability to inhibit conidia germination. Spore suspensions and fungicide solutions were mixed with sterilized water to 10 mL volume. The final fungicide concentrations were 0.005, 0.01, 0.05, 0.1, 0.5, and 1 μg/mL, while spore suspension was adjusted to 1 × 10^5^ spores/mL for each treatment. A 20 μL droplet of each suspension was placed on a hydrophobic cover slip and incubated at 25°C for 18–20 h in a humidity chamber according to Fang et al. (2018). Each treatment was conducted in triplicate, and the entire experiment was repeated twice. Conidia were then observed at ×100 magnification using a ZEISS microscope and scored as germinated if the length of the germ tube was longer than half of the conidial length. The conidial germination inhibition rate was calculated as previously described (Munir et al., 2016).

## Results

### Field Symptoms and *Colletotrichum* Isolates

In May 2018, typical symptoms of anthracnose were first observed on newly emerged leaves of *C. paliurus* in a commercial nursery in Baima (Figure 1A), and the infection quickly spread to all *C. paliurus* nurseries within the growing season, with the infection rate reaching 64% (150 trees were investigated). Similar symptoms were observed in plant bases at Changzhou and Yancheng, with infection rates over 35 and 45% (100 trees were investigated), respectively. The initial symptoms appeared in the form of subcircular or irregular pale-brown spots scattered on the leaves (Figure 1C). Gradually, the lesions enlarged and coalesced to form large necrotic areas, which turned off-white surrounded by a dark-brown border as symptoms progressed (Figure 1B). The dead tissue withered, resulting in premature defoliation of the plant in severe cases (Figure 1A). Under high-moisture conditions, a number of acervuli were formed in concentric rings and oozed gelatinous orange spore masses (Figure 1D). Photomicrographs further corroborated the presence of conidiophores and conidia on the surfaces of leaf lesions under optical or SEM microscopy (Figures 1E,F).

**FIGURE 2:**
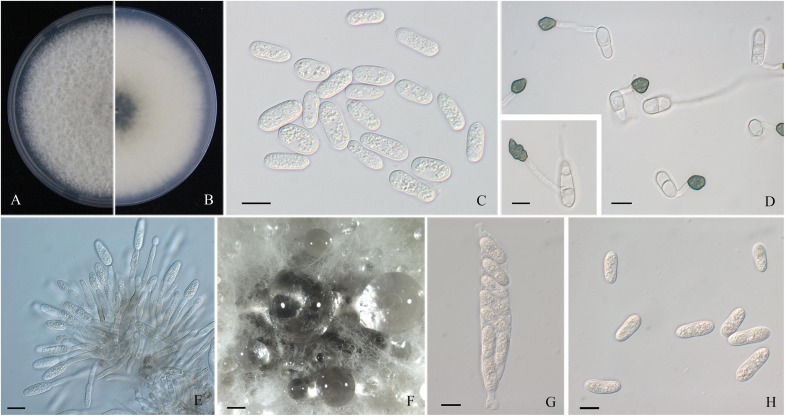
Morphological characters of *Colletotrichum aenigma*. **(A,B)** Front and back view, respectively, of 6-days-old PDA culture. **(C)** Conidia. **(D)** Appressoria. **(E)** Conidiophores. **(F)** Ascomata developed on PDA plates. **(G)** Asci. **(H)** Ascospores. Scale bars: **(C–E,G,H)** = 10 μm; **(F)** = 500 μm.

**FIGURE 3:**
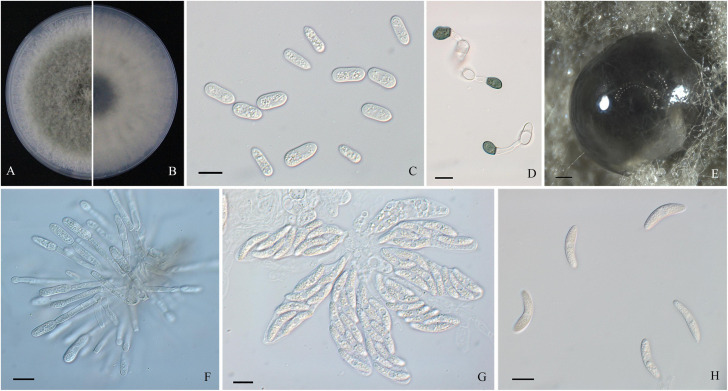
Morphological characters of *Colletotrichum fructicola*. **(A,B)** Front and back view, respectively, of 6-days-old PDA culture. **(C)** Conidia. **(D)** Appressoria. **(E)** Ascomata developed on PDA plates. **(F)** Conidiophores. **(G)** Asci. **(H)** Ascospores. Scale bars: **(C,D,F–H)** = 10 μm; **(E)** = 200 μm.

**FIGURE 4:**
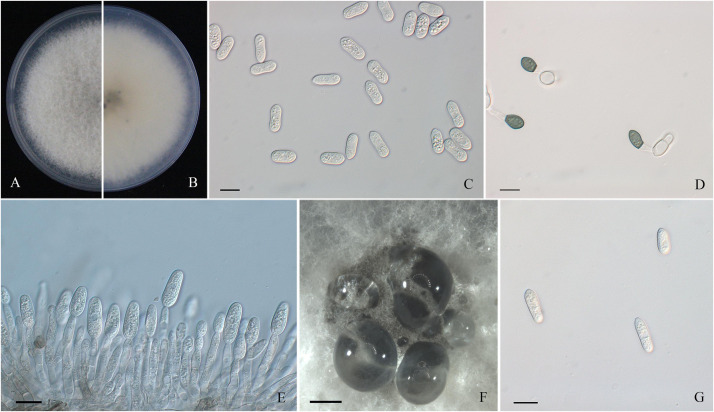
Morphological characters of *Colletotrichum gloeosporioides* sensu stricto. **(A,B)** Front and back view, respectively, of 6-days-old PDA culture. **(C)** Conidia. **(D)** Appressoria. **(E)** Conidiophores. **(F)** Ascomata developed on PDA plates. **(G)** Ascospores. Scale bars: **(C–E,G)** = 10 μm; **(F)** = 500 μm.

A total of 44 monosporic *Colletotrichum* isolates were recovered from symptomatic tissues and used for further molecular identification (Table 1). The shapes and sizes of conidia of these cultures were basically concordant with the sporulation on the lesions (Figures 2C, 3C, 4C). The general morphological characteristics of all isolates resembled those of *Colletotrichum* species.

### Molecular Identification and Phylogenetic Analysis

In the present study, the ITS, CAL, ACT, GPDH, TUB, CHS-1, GS, and ApMat region/genes of all 44 monosporic isolates were successfully amplified and sequenced (Table 1). Sequences generated herein along with reference sequences from ex-type or other authoritative specimens were concatenated for phylogeny construction, composing a dataset of 3192 characters, with 1828 constant characters, 574 parsimony-uninformative characters, and 790 parsimony-informative characters.

**FIGURE 5:**
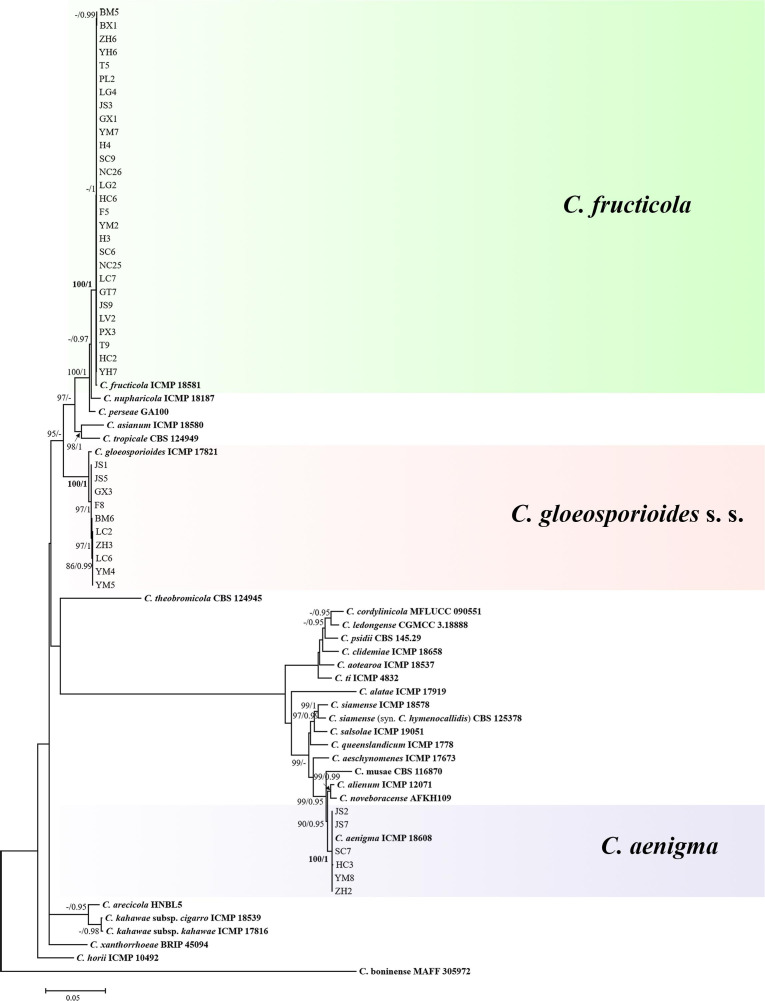
Maximum-likelihood phylogenetic tree of 66 isolates of the *Colletotrichum gloeosporioides* species complex. The tree was built using concatenated sequences of the ITS, GAPDH, CAL, ACT, CHS-1, TUB, GS, and ApMat region or genes, each with a separate model of DNA evolution. The tree generated by Bayesian inference had a similar topology. Bootstrap support values above 85% and Bayesian posterior probability values above 0.95 are shown at the nodes. Ex-type or other authoritative cultures are emphasized in bold font. *C. boninense* (MAFF 305972) was used as an outgroup. The scale bar indicates the average number of substitutions per site.

The topological structure of the phylogenetic trees constructed using BI and ML criteria was basically consistent, demonstrating that the evolutionary relationships of the experimental strains were statistically supported. A consensus tree with clade support from bootstrap proportions (BPs) and PP values was generated (Figure 5). The phylogenetic tree revealed that all 44 *Colletotrichum* isolates belonged to three well-separated clades and nested within the *C. gloeosporioides* species complex. Six *Colletotrichum* isolates composed a highly supported clade (100% BP/1.00 PP) with the *Colletotrichum aenigma* type strain ICMP 18608. Twenty-eight isolates belonged to the other highly supported clade (100% BP/1.00 PP) along with the *Colletotrichum fructicola* type strain ICMP 18581. Ten isolates clustered in another highly supported clade (100% BP/1.00 PP) with the *C. gloeosporioides* s. s. type strain IMI 356878 (Figure 5).

### Morphological and Biological Analyses

Fourteen representative isolates clustered in three clades in the ML/BI phylogenetic analysis, including three of *C. aenigma*, seven of *C. fructicola*, and four of *C. gloeosporioides* s. s., were selected for further studies (Table 1).

Colonies of *C. fructicola* isolates produced abundant grayish-green aerial hyphae with white halo edges, and the back of the colony was grayish-green with concentric rings (Figures 3A,B). Isolates of *C. aenigma* and *C. gloeosporioides* s. s. exhibited white or gray mycelia, and the back of the colony was densely arranged with a grayish-green color in the center (Figures 2A,B, 4A,B). There were few differences in the shapes of conidia, conidiophores, and appressoria among the three species. Conidia were all one-celled, hyaline, smooth-walled, mostly cylindrical with broadly rounded ends, and sometimes slightly and gradually acute to the end (Figures 2C, 3C, 4C). The average conidial sizes for isolates were as follows: *C. aenigma*, 14.3–26.6 × 5.56–11.13 μm; *C. fructicola*, 12–23.14 × 4.5–9.79 μm; and *C. gloeosporioides* s. s., 13.44–24.82 × 4.95–9.69 μm (Table 3). Conidiophores were smooth-walled, septate, and hyaline to pale brown (Figures 2E, 3F, 4E). Appressoria were dark brown, subglobose or ellipsoid, and rarely irregular (Figures 2D, 3D, 4D). The average appressorium sizes for the isolates were as follows: *C. aenigma*, 8.35–16.92 × 5.87–11.73 μm; *C. fructicola*, 8.04–14.15 × 6.45–9.97 μm; and *C. gloeosporioides* s. s., 9.13–13.91 × 6.06–10.61 μm (Table 3). Ascomata of three *Colletotrichum* species formed on PDA at 20 dpi and were semi-immersed in agar medium, dark-brown, and subglobose to pyriform (Figures 2F, 3E, 4F). Asci were clavate, fasciculate, and eight-spored in most cases, while asci of *C. gloeosporioides* s. s. were not observed (Figures 2G, 3G). Ascospores of *C. aenigma* isolates were hyaline, smooth-walled, aseptate, cylindrical, and 15.94–22.36 × 5.56–9.17 μm in size (Table 3 and Figure 2H). Ascospores of *C. gloeosporioides* s. s. isolates were hyaline, smooth-walled, aseptate, cylindrical, and 9.02–17.07 × 3.62–5.98 μm in size (Table 3 and Figure 4G). Ascospores of *C. fructicola* were hyaline, aseptate, smooth-walled, fusoid, slightly curved, straight with round ends, and 13.23–25.35 × 3.1–6.19 μm in size (Table 3 and Figure 3H).

All 14 representative isolates tested exhibited a similar growth pattern on PDA at the different treatment temperatures. No mycelial growth of any tested isolates was observed *in vitro* at 5°C. The optimum mycelial growth temperature of the three *Colletotrichum* species was 25–30°C, but the high temperature tolerance of the three species was different. Isolates of *C. aenigma* and *C. fructicola* were more sensitive to high temperature and grew very slowly (or could not grow) at 40°C, with mean growth rates lower than those of *C. gloeosporioides* s. s. isolates.

### Virulence Tests of *Colletotrichum* Isolates

**FIGURE 6:**
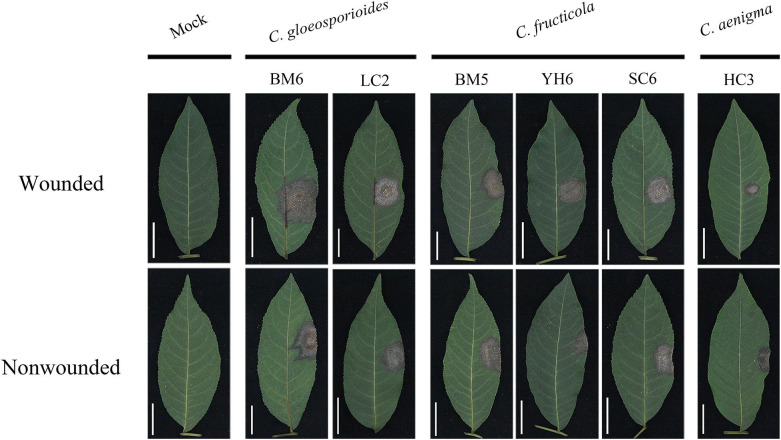
Typical symptoms induced by mycelial discs of representative isolates of *C. gloeosporioides* sensu stricto, *C. fructicola*, and *C. aenigma* on wounded (upper) and unwounded (lower) detached leaves of *Cyclocarya paliurus*.

All 14 selected isolates were pathogenic on leaves of *C. paliurus* and reproduced typical symptoms of anthracnose. Seven days after wounded or non-wounded inoculation, distinct brown or off-white necrotic lesions with dark-brown boundaries developed (Figure 6), while no symptoms developed on the corresponding mock controls.

The severity of disease caused by these isolates showed significant differences (Table 4). Isolates of *C. gloeosporioides* s. s. generally showed strong virulence, with mean lesion diameters ranging from 17.88 to 23.16 and 17.52 to 22.11 mm with wounded and non-wounded inoculation using mycelial plugs as inocula, respectively. There was no significant difference in virulence among *C. fructicola* isolates in *C. paliurus* leaves, with mean lesion diameters ranging from 16.65 to 20.52 and 17.41 to 21.09 mm with wounded and non-wounded inoculation using mycelial plugs as inocula, respectively. *C. aenigma* isolates showed much weaker virulence, with mean lesion diameters ranging from 12.38 to 14.89 and 11.78 to 14.12 mm with wounded and non-wounded inoculation using mycelial plugs as inocula, respectively. The lesions produced by mycelial inoculation were generally larger than those produced by spore suspension inoculation among the three *Colletotrichum* species (Table 4). *Colletotrichum gloeosporioides* s. s. isolate BM6 and *C. fructicola* isolate BM5 produced reproductive structures of the fungus on the necrotic lesions (Figure 6). *C. fructicola* isolate YH6 produced lesions with a wheel-shaped pattern on *C. paliurus* leaves (Figure 6). The *Colletotrichum* species were reisolated from all inoculated symptomatic leaves and were found to be morphologically and molecularly identical to the original isolates using the aforementioned methods, thus fulfilling Koch’s postulates.

**TABLE 4:** Pathogenicity of *Colletotrichum* isolates on detached leaves of *Cyclocarya paliurus*.

Species/Isolates	Infected leaves (%)^a^				Lesion diameter (mm)^b^			
	Conidial suspension		Mycelial plug		Conidial suspension		Mycelial plug	
	Wounded	Non-wounded	Wounded	Non-wounded	Wounded	Non-wounded	Wounded	Non-wounded
CK	–	–	–	–	–	–	–	–
***C. aenigma***								
HC3	80.00 ± 11.55	60.00 ± 0.00	100.00 ± 0.00	86.67 ± 6.67	9.63 ± 0.9^b^	5.79 ± 0.56^b^	12.38 ± 1.10^f^	14.12 ± 1.28*cd*
SC7	93.33 ± 6.67	60.00 ± 0.00	100.00 ± 0.00	93.33 ± 6.67	9.58 ± 0.96^b^	5.77 ± 0.48^b^	13.10 ± 0.82*ef*	14.44 ± 0.92*cd*
YM8	73.33 ± 6.67	66.67 ± 6.67	100.00 ± 0.00	86.67 ± 6.67	8.9 ± 0.81^b^	5.41 ± 0.39^b^	14.89 ± 1.07*def*	11.78 ± 1.02^d^
***C. fructicola***								
BM5	93.33 ± 6.67	80.00 ± 11.55	100.00 ± 0.00	100.00 ± 0.00	17.69 ± 0.99^a^	15.85 ± 1.26^a^	20.52 ± 0.61*abc*	20.37 ± 0.91*ab*
GX1	93.33 ± 6.67	73.33 ± 6.67	100.00 ± 0.00	93.33 ± 6.67	16.44 ± 1.07^a^	13.85 ± 1.26^a^	18.75 ± 0.76*bcd*	20.16 ± 0.98*ab*
HC2	86.67 ± 6.67	80.00 ± 11.55	100.00 ± 0.00	100.00 ± 0.00	16.41 ± 1.16^a^	13.86 ± 1.27^a^	18.18 ± 1.06*bcd*	19.75 ± 1.03*abc*
LC7	86.67 ± 6.67	86.67 ± 6.67	100.00 ± 0.00	93.33 ± 6.67	16.21 ± 1.03^a^	14.73 ± 1.49^a^	20.51 ± 0.94*abc*	18.59 ± 1.76*abc*
NC25	93.33 ± 6.67	86.67 ± 13.33	100.00 ± 0.00	93.33 ± 6.67	16.34 ± 0.88^a^	13.41 ± 1.03^a^	18.93 ± 1.07*bcd*	21.09 ± 0.95^a^
SC6	93.33 ± 6.67	73.33 ± 6.67	100.00 ± 0.00	93.33 ± 6.67	15.45 ± 1.16^a^	12.93 ± 1.52^a^	16.90 ± 0.94*cde*	18.06 ± 1.54*abc*
YH6	93.33 ± 6.67	80.00 ± 11.55	100.00 ± 0.00	100.00 ± 0.00	15.56 ± 1.04^a^	13.87 ± 1.12^a^	16.65 ± 0.87*cde*	17.41 ± 1.14*abc*
***C. gloeosporioides***								
BM6	86.67 ± 6.67	80.00 ± 11.55	100.00 ± 0.00	100.00 ± 0.00	18.43 ± 0.93^a^	15.92 ± 1.14^a^	23.16 ± 0.80^a^	22.11 ± 0.66^a^
GX3	86.67 ± 6.67	86.67 ± 6.67	100.00 ± 0.00	100.00 ± 0.00	17.24 ± 0.91^a^	16.35 ± 0.87^a^	20.29 ± 0.81*abc*	19.03 ± 1.03*abc*
LC2	86.67 ± 6.67	80.00 ± 11.55	100.00 ± 0.00	100.00 ± 0.00	17.27 ± 0.97^a^	12.98 ± 0.86^a^	17.88 ± 0.61*bcd*	17.52 ± 1.68*abc*
YM4	93.33 ± 6.67	80.00 ± 11.55	100.00 ± 0.00	100.00 ± 0.00	17.44 ± 0.63^a^	15.21 ± 1.49^a^	20.40 ± 0.58*abc*	21.39 ± 1.03^a^

*^a,b^Values were means ± standard error of three replications. Means with different letters indicate mean lesion lengths that are significantly different (P < 0.05). Data were calculated using disease incidence of 15 inoculated leaves. – represents no symptom developed on inoculated site. ^CK^Detached C. paliurus leaves were inoculated with sterile water or PDA plugs without the pathogens (as controls).*

### Sensitivity of *Colletotrichum* Isolates to Biofungicides

Fourteen representative isolates evaluated showed similar biological responses to all tested biofungicides. Kasugamycin at 50 mg/mL showed no suppressive activity against the mycelial growth of the three *Colletotrichum* spp. on CM medium (EC_50_ > 100 μg/mL). PCA showed moderate inhibition of the mycelial growth of the three *Colletotrichum* spp., with isolates of *C. fructicola* exhibiting more sensitivity to this biofungicide. The EC_50_ of tetramycin against the mycelial growth of all representative isolates was lower than that of any of the other biofungicides, including the low EC_50_ of tetramycin against spore germination, indicating that tetramycin was the most effective biofungicide against the three *Colletotrichum* spp. used in this study.

## Discussion

In recent years, the cultivation of *C. paliurus* has undergone a major expansion to meet the increasing demand for young leaves of this species for medical use or *C. paliurus* tea production in China, which may have caused the high incidence of foliar diseases in these newly established plantations. Therefore, it is of great importance to diagnose and control these fungal diseases of *C. paliurus*. Unfortunately, little information was available about these diseases, i.e., *C. paliurus* anthracnose. Hitherto, this study is first comprehensive analysis demonstrating the etiology of *C. paliurus* anthracnose in China, providing valuable information about the phenotypic and molecular characteristics, virulence, and fungicide sensitivity of the causal agents associated with this disease. Moreover, this study provides the first report of *C. aenigma*, *C. fructicola*, and *C. gloeosporioides* s. s. causing *C. paliurus* anthracnose in China as well as in the world.

*Colletotrichum gloeosporioides* species complex is regarded as the most challenging taxa within the *Colletotrichum* genus (Silva et al., 2012). Although polyphasic method is recommended for characterizing *Colletotrichum* species, there is still lack of consensus among taxonomists on the selection of markers for phylogenetic studies (Cao et al., 2020; Vieira et al., 2020). Recent studies revealed that concatenated GS and ApMat alignment can achieve a satisfactory *Colletotrichum* species identification (Liu et al., 2015; Sharma et al., 2017). Conservative region/genes (ITS, GAPDH, CAL, CHS-1, ACT, and TUB) have been previously accepted for delimiting species in this species complex (Weir et al., 2012). Therefore, in the present study, eight loci (ITS, GAPDH, CAL, CHS-1, ACT, TUB, including GS and ApMat) were selected in phylogenetic analysis for *Colletotrichum* isolates classification. Based on BI/ML multilocus concatenated phylogenetic analyses, including sequences from 28 authentic specimens in the *C. gloeosporioides* species complex, the 44 isolates were categorized into three well-separated clades: six isolates clustered in the *C. aenigma* clade (14%), 28 isolates clustered in the *C. fructicola* clade (64%), and 10 isolates clustered in the *C. gloeosporioides* s. s. clade (22%). With respect to phenotypic characterization based on colony morphology, characteristics of conidia, appressoria, ascospores, and asci were entirely in line with the results of the molecular data.

*Colletotrichum fructicola* was first described by Prihastuti et al. (2009), causing coffee berry disease in Thailand. The species is geographically diverse and threatens a wide range of hosts, which has been reported on *Fragaria* × *ananassa* and *Malus* sp. (United States), *Ficus* sp. (Germany), *Persea americana* (Australia), *Pyrus pyrifolia* (Japan), *Limonium* sp. (Israel), *Tetragastris* sp. and *Theobroma* sp. (Panama), *Dioscorea* sp. (Nigeria), *Malus* sp. (Brazil) (Weir et al., 2012), and *Mangifera indica* (China) (Mo et al., 2018). In the current study, *C. fructicola* was the most predominant species and exhibited strong pathogenicity (Table 4), which seems to be the most economically harmful species of *C. paliurus* anthracnose in Jiangsu Province, China.

*Colletotrichum gloeosporioides* s. s., a genetically and biologically diverse species, previously reported to infect fruits in tropical area (Sangeetha and Rawal, 2008; Udayanga et al., 2013), which is probably related to its ability to tolerate high temperatures (Table 5). However, this species was recently reported increasingly prevalent in the temperate region, such as Hebei, Shandong, and Shanxi Provinces of China (Jayawardena et al., 2016; Wang et al., 2020). Results in the present study demonstrated that *C. gloeosporioides* showed the strongest pathogenicity to *C. paliurus* (Table 4). The prevalence and ecological adaptation zone of *C. gloeosporioides* on *C. paliurus* in China would be further studied.

**TABLE 5:** Growth rate (mm/4d) of *Colletotrichum* isolates from *Cyclocarya paliurus* cultured on PDA at different temperatures.

Species/Isolates	5°C	10°C	15°C	20°C	25°C	30°C	35°C	40°C
***C. aenigma***								
HC3	0a	2.01 ± 0.1*ab*	3.92 ± 0.09*abc*	7.67 ± 0.01*abc*	12.43 ± 0.14^b^	12.39 ± 0.14^a^	7.6 ± 0.57^a^	2.4 ± 1.22*ab*
SC7	0a	2.26 ± 0.2^a^	3.77 ± 0.12*abc*	7.66 ± 0.04*abc*	12.66 ± 0.19^b^	12.62 ± 0.09^a^	7.69 ± 0.08^a^	2.13 ± 1.11*ab*
YM8	0a	2.24 ± 0.17^a^	3.79 ± 0.15*abc*	7.96 ± 0.05*abc*	12.28 ± 0.31^b^	12.19 ± 0.08^a^	7.72 ± 0.56^a^	0*b*
***C. fructicola***								
BM5	0a	1.11 ± 0.56*ab*	3.54 ± 0.22*abc*	8.81 ± 0.09*ab*	15.49 ± 0.04^a^	12.93 ± 0.28^a^	5.64 ± 0.48^b^	2.71 ± 1.36*ab*
GX1	0a	1.79 ± 0.12*ab*	3.61 ± 0.09*abc*	8.32 ± 0.28*abc*	15.81 ± 0.04^a^	13.56 ± 0.25^a^	5.17 ± 0.42^b^	2.71 ± 1.35*ab*
HC2	0a	1.62 ± 0.18*ab*	3.2 ± 0.19*abc*	8.46 ± 0.49*abc*	15.42 ± 0.12^a^	13.33 ± 0.27^a^	4.91 ± 0.08^b^	0*b*
LC7	0a	1.36 ± 0.14*ab*	3.72 ± 0.16*abc*	8.96 ± 0.07^a^	15.84 ± 0.07^a^	12.99 ± 0.45^a^	5.59 ± 0.11^b^	1.99 ± 0.05*ab*
NC25	0a	0.91 ± 0.46^b^	3.35 ± 0.19*abc*	8.56 ± 0.35*abc*	15.68 ± 0.13^a^	12.93 ± 0.34^a^	5.36 ± 0.46^b^	2.66 ± 1.33*ab*
SC6	0a	1.68 ± 0.28*ab*	3.27 ± 0.35*bc*	8.12 ± 0.17*abc*	15.74 ± 0.17^a^	13.45 ± 0.45^a^	5.44 ± 0.34^b^	0*b*
YH6	0a	1.61 ± 0.21*ab*	3.64 ± 0.24*abc*	8.28 ± 0.35*abc*	15.9 ± 0.15^a^	13.42 ± 0.14^a^	5.7 ± 0.47^b^	2.08 ± 0.13*ab*
***C. gloeosporioides***								
BM6	0a	1.84 ± 0.07*ab*	4.27 ± 0.13^a^	7.37 ± 0.38^c^	11.54 ± 0.36*bc*	12.37 ± 0.65^a^	8.12 ± 0.21^a^	3.36 ± 0.99*ab*
GX3	0a	1.89 ± 0.17*ab*	4.21 ± 0.2*ab*	7.62 ± 0.13*bc*	11.62 ± 0.46*bc*	12.66 ± 0.17^a^	8.35 ± 0.2^a^	5.25 ± 1.13^a^
LC2	0a	1.76 ± 0.19*ab*	4.24 ± 0.13^a^	7.57 ± 0.29*bc*	10.88 ± 0.26^c^	11.75 ± 0.18^a^	8.1 ± 0.12^a^	3.2 ± 0.76*ab*
YM4	0a	1.93 ± 0.19*ab*	4.26 ± 0.17^a^	7.73 ± 0.38*abc*	12.04 ± 0.43*bc*	12.42 ± 0.8^a^	8.01 ± 0.15^a^	2.43 ± 0.74*ab*

*Columns with the same letter do not differ significantly according to Tukey’s test (P < 0.05).*

Interestingly, multiple *Colletotrichum* species were isolated and identified from the same leaf and even within the same lesion of single *C. paliurus* trees. As reported in previous studies, several *Colletotrichum* species can cause anthracnose on the same host (Munir et al., 2016; Chen Y. et al., 2017; De Silva et al., 2017a; Diao et al., 2017; Guarnaccia et al., 2017; Fu et al., 2019; Xue et al., 2019). It is reasonable to believe that *C. paliurus* anthracnose may be a complex disease. With more samples collected, it is possible that even more *Colletotrichum* species, or even novel species, will be characterized as responsible for this disease. Consequently, future attention should be given to probe *Colletotrichum* species collected from *C. paliurus* anthracnose in different geographical areas with different latitudes or elevations in China.

Temperature is an indispensable factor that affects epidemics of anthracnose or other plant diseases (Dubrulle et al., 2020). High temperatures and their frequency may be the factors leading to the delay or non-occurrence of plant diseases (Han et al., 2016; Xue et al., 2019). In the present study, no significant differences occurred in the optimum and minimum mycelial growth temperatures of *C. aenigma* and *C. fructicola*, while *C. gloeosporioides* s. s. isolates exhibited more tolerance to high temperature, which was in concordance with previous study results (Han et al., 2016). These data may provide useful information for *C. paliurus* anthracnose control strategies: fungicide applications should be timed before the optimum growth temperature is reached.

Once infection occurs, the suppression of spore germination and mycelial growth within the plant tissue plays a crucial role in anthracnose management. In the present study, tetramycin showed excellent inhibitory effect on the mycelial growth and spore germination of the three *Colletotrichum* species (Table 6). The satisfactory inhibitory activity against different life stages of *Colletotrichum* species indicates that tetramycin may be a potential alternative for the management of *C. paliurus* anthracnose. In previous studies, the excellent curative and protective activity of tetramycin has been widely reported in Phytophthora blight, rice blast, tomato leaf mold, Corynespora leaf spot, and cucumber gray mold (Zhao et al., 2010; Miao et al., 2015; Song et al., 2016; Chen L. L. et al., 2017; Ma et al., 2018a, b), demonstrating that tetramycin would be helpful to prevent the occurrence and spread of plant diseases throughout the field. Accordingly, protective and curative activity of tetramycin on *C. paliurus* anthracnose in the field trials would be further studied before it is put into use.

**TABLE 6:** Mean half-maximal effective concentration (EC_50_) of *Colletotrichum* spp.

Species/Isolate	EC_50_ (mg/liter)^z^			
	Mycelial growth			Spore germination
	Tetramycin	PCA	Kasugamycin	Tetramycin
***C. aenigma***				
HC3	2.5 ± 0.01	22.95 ± 6.39	>100	0.02 ± 0.00
SC7	2.59 ± 0.11	42.05 ± 12.64	>100	0.15 ± 0.01
YM8	2.51 ± 0.01	39.5 ± 14.03	>100	0.02 ± 0.01
***C. fructicola***				
BM5	2.61 ± 0.12	19.54 ± 2.18	>100	0.02 ± 0.01
GX1	2.41 ± 0.08	24.67 ± 4.42	>100	0.02 ± 0.01
HC2	2.65 ± 0.22	24.67 ± 5.3	>100	0.02 ± 0.01
LC7	2.46 ± 0.11	32.24 ± 5.03	>100	0.03 ± 0.02
NC25	2.44 ± 0.01	20.14 ± 0.93	>100	0.01 ± 0.00
SC6	2.45 ± 0.13	26.16 ± 4.85	>100	0.02 ± 0.00
YH6	2.59 ± 0.07	15.61 ± 1.49	>100	0.02 ± 0.00
***C. gloeosporioides***				
BM6	3.15 ± 0.46	40.71 ± 9.07	>100	0.04 ± 0.01
GX3	2.6 ± 0.19	40.47 ± 9.56	>100	0.02 ± 0.00
LC2	3.1 ± 0.46	40.21 ± 7.54	>100	0.02 ± 0.00
YM4	3.03 ± 0.35	38.03 ± 9.7	>100	0.01 ± 0.00

*^z^Data are mean ± standard error.*

## Data Availability Statement

The datasets presented in this study can be found in online repositories. The names of the repository/repositories and accession number(s) can be found in the article/Supplementary Material.

## Author Contributions

X-RZ was responsible for the entire process of experimentation and writing the manuscript. M-JZ helped perform the experiment and analyze the results. X-LS and S-ZF provided experimental materials. F-MC supervised the work. All authors contributed to manuscript revision and read and approved the submitted version.

## Conflict of Interest

The authors declare that the research was conducted in the absence of any commercial or financial relationships that could be construed as a potential conflict of interest.

## Footnotes

1 https://www.federalregister.gov/documents/2014/08/29/2014-20502/kasugamycin-pesticide-tolerances

2 http://www.mbio.ncsu.edu/bioedit/page2.html
