# Supplementary material for: Etiology of Cyclocarya paliurus Anthracnose in Jiangsu Province, China
Source: Front Plant Sci. 2021 Jan 18;11:613499. doi: 10.3389/fpls.2020.613499 (PMC7847979; doi:10.3389/fpls.2020.613499)
Supplement: Supplementary file 1 [file Table_1.DOCX]

>HC3

TCTTGATGATGTATCCCGACTACCGTTACACCCCTCGCAAGCCGTCTGAGAAGCGCCATC

GAAAGCCTAGTGGCCAAAGCAAGAAGACCAGCTTAGCAGCGTCGATGAGGTAGAGGCCGC

CTCCTACCCGAGAGCGACAAAACCATCAGCGCGCCGACTTGTCGGCTCCAGACGGAGGAG

TATGAGTTATACCTACCGACTGCGACAAGTCGCTGTAGTATCAGTCATGGAACGGAATAG

ATATCGGCGCTGCCAGACCACCTTAGGTCGTCCTTGTGTTCTAGACTGGCAGCATCAGCG

ACCAGAGGAGCCTCAGGACATGGCGCCTTTGGAGGCTGTCTTCGATCTCTATCCAGTCAG

CGTGACCCAGGAGGCGACCACGCATCTGGTCGTCGATTCACTTCCCGCATGCTGTGGCAA

GAGTTACCTGCGCGAGAAACCAACAGACCTTTGTGCGGCGATACTTAGACGTACGAACGC

TAGGAGATCACCGGATCTGAGGAACCAAGAATCATCCTGGACCATCTGCTTCGATGGCAA

CGCGAAGCAGAGATCTTCGATTTGATGAACATATTGGTGCAAAAATGTACTCACAACCCA

TGTATTCGCATGTCAAGCGAACACGAATCTTTTTCTGTAATTATAATCTGCAAGCCAGTA

AATATCATCTTCATGGACCATCTATTCCAGCCCGATACCTAGAGTGTCTTTTGCCCCCCG

CCATGAGGACCATTGATTCGCCCACATGTTGCTCTTCGCCACTGCAGACTGCCAACGCCA

TCCATGATGCCCAGCCACTGTCTCGACTGCGCATCACGGGAATAACTACGCCTGATCTTT

TTTCCAGTCGCTGCTCCAGATGAACGTCCCGCATCTCCACTCCGTCCCCGATTCTTTGAC

TCCCGACTTCCCCAACGGGCGGGCACATACGTAGAAATGA

>JS2

TCTTGATGATGTATCCCGACTACCGTTACACCCCTCGCAAGCCGTCTGAGAAGCGCCATC

GAAAGCCTAGTGGCCAAAGCAAGAAGACCAGCTTAGCAGCGTCGATGAGGTAGAGGCCGC

CTCCTACCCGAGAGCGACAAAACCATCAGCGCGCCGACTTGTCGGCTCCAGACGGAGGAG

TATGAGTTATACCTACCGACTGCGACAAGTCGCTGTAGTATCAGTCATGGAACGGAATAG

ATATCGGCGCTGCCAGACCACCTTAGGTCGTCCTTGTGTTCTAGACTGGCAGCATCAGCG

ACCAGAGGAGCCTCAGGACATGGCGCCTTTGGAGGCTGTCTTCGATCTCTATCCAGTCAG

CGTGACCCAGGAGGCGACCACGCATCTGGTCGTCGATTCACTTCCCGCATGCTGTGGCAA

GAGTTACCTGCGCGAGAAACCAACAGACCTTTGTGCGGCGATACTTAGACGTACGAACGC

TAGGAGATCACCGGATCTGAGGAACCAAGAATCATCCTGGACCATCTGCTTCGATGGCAA

CGCGAAGCAGAGATCTTCGATTTGATGAACATATTGGTGCAAAAATGTACTCACAACCCA

TGTATTCGCATGTCAAGCGAACACGAATCTTTTTCTGTAATTATAATCTGCAAGCCAGTA

AATATCATCTTCATGGACCATCTATTCCAGCCCGATACCTAGAGTGTCTTTTGCCCCCCG

CCATGAGGACCATTGATTCGCCCACATGTTGCTCTTCGCCACTGCAGACTGCCAACGCCA

TCCATGATGCCCAGCCACTGTCTCGACTGCGCATCACGGGAATAACTACGCCTGATCTTT

TTTCCAGTCGCTGCTCCAGATGAACGTCCCGCATCTCCACTCCGTCCCCGATTCTTTGAC

TCCCGACTTCCCCAACGGGCGGGCACATACGTAGAAATGA

>JS7

TCTTGATGATGTATCCCGACTACCGTTACACCCCTCGCAAGCCGTCTGAGAAGCGCCATC

GAAAGCCTAGTGGCCAAAGCAAGAAGACCAGCTTAGCAGCGTCGATGAGGTAGAGGCCGC

CTCCTACCCGAGAGCGACAAAACCATCAGCGCGCCGACTTGTCGGCTCCAGACGGAGGAG

TATGAGTTATACCTACCGACTGCGACAAGTCGCTGTAGTATCAGTCATGGAACGGAATAG

ATATCGGCGCTGCCAGACCACCTTAGGTCGTCCTTGTGTTCTAGACTGGCAGCATCAGCG

ACCAGAGGAGCCTCAGGACATGGCGCCTTTGGAGGCTGTCTTCGATCTCTATCCAGTCAG

CGTGACCCAGGAGGCGACCACGCATCTGGTCGTCGATTCACTTCCCGCATGCTGTGGCAA

GAGTTACCTGCGCGAGAAACCAACAGACCTTTGTGCGGCGATACTTAGACGTACGAACGC

TAGGAGATCACCGGATCTGAGGAACCAAGAATCATCCTGGACCATCTGCTTCGATGGCAA

CGCGAAGCAGAGATCTTCGATTTGATGAACATATTGGTGCAAAAATGTACTCACAACCCA

TGTATTCGCATGTCAAGCGAACACGAATCTTTTTCTGTAATTATAATCTGCAAGCCAGTA

AATATCATCTTCATGGACCATCTATTCCAGCCCGATACCTAGAGTGTCTTTTGCCCCCCG

CCATGAGGACCATTGATTCGCCCACATGTTGCTCTTCGCCACTGCAGACTGCCAACGCCA

TCCATGATGCCCAGCCACTGTCTCGACTGCGCATCACGGGAATAACTACGCCTGATCTTT

TTTCCAGTCGCTGCTCCAGATGAACGTCCCGCATCTCCACTCCGTCCCCGATTCTTTGAC

TCCCGACTTCCCCAACGGGCGGGCACATACGTAGAAATGA

>SC7

TCTTGATGATGTATCCCGACTACCGTTACACCCCTCGCAAGCCGTCTGAGAAGCGCCATC

GAAAGCCTAGTGGCCAAAGCAAGAAGACCAGCTTAGCAGCGTCGATGAGGTAGAGGCCGC

CTCCTACCCGAGAGCGACAAAACCATCAGCGCGCCGACTTGTCGGCTCCAGACGGAGGAG

TATGAGTTATACCTACCGACTGCGACAAGTCGCTGTAGTATCAGTCATGGAACGGAATAG

ATATCGGCGCTGCCAGACCACCTTAGGTCGTCCTTGTGTTCTAGACTGGCAGCATCAGCG

ACCAGAGGAGCCTCAGGACATGGCGCCTTTGGAGGCTGTCTTCGATCTCTATCCAGTCAG

CGTGACCCAGGAGGCGACCACGCATCTGGTCGTCGATTCACTTCCCGCATGCTGTGGCAA

GAGTTACCTGCGCGAGAAACCAACAGACCTTTGTGCGGCGATACTTAGACGTACGAACGC

TAGGAGATCACCGGATCTGAGGAACCAAGAATCATCCTGGACCATCTGCTTCGATGGCAA

CGCGAAGCAGAGATCTTCGATTTGATGAACATATTGGTGCAAAAATGTACTCACAACCCA

TGTATTCGCATGTCAAGCGAACACGAATCTTTTTCTGTAATTATAATCTGCAAGCCAGTA

AATATCATCTTCATGGACCATCTATTCCAGCCCGATACCTAGAGTGTCTTTTGCCCCCCG

CCATGAGGACCATTGATTCGCCCACATGTTGCTCTTCGCCACTGCAGACTGCCAACGCCA

TCCATGATGCCCAGCCACTGTCTCGACTGCGCATCACGGGAATAACTACGCCTGATCTTT

TTTCCAGTCGCTGCTCCAGATGAACGTCCCGCATCTCCACTCCGTCCCCGATTCTTTGAC

TCCCGACTTCCCCAACGGGCGGGCACATACGTAGAAATGA

>YM8

TCTTGATGATGTATCCCGACTACCGTTACACCCCTCGCAAGCCGTCTGAGAAGCGCCATC

GAAAGCCTAGTGGCCAAAGCAAGAAGACCAGCTTAGCAGCGTCGATGAGGTAGAGGCCGC

CTCCTACCCGAGAGCGACAAAACCATCAGCGCGCCGACTTGTCGGCTCCAGACGGAGGAG

TATGAGTTATACCTACCGACTGCGACAAGTCGCTGTAGTATCAGTCATGGAACGGAATAG

ATATCGGCGCTGCCAGACCACCTTAGGTCGTCCTTGTGTTCTAGACTGGCAGCATCAGCG

ACCAGAGGAGCCTCAGGACATGGCGCCTTTGGAGGCTGTCTTCGATCTCTATCCAGTCAG

CGTGACCCAGGAGGCGACCACGCATCTGGTCGTCGATTCACTTCCCGCATGCTGTGGCAA

GAGTTACCTGCGCGAGAAACCAACAGACCTTTGTGCGGCGATACTTAGACGTACGAACGC

TAGGAGATCACCGGATCTGAGGAACCAAGAATCATCCTGGACCATCTGCTTCGATGGCAA

CGCGAAGCAGAGATCTTCGATTTGATGAACATATTGGTGCAAAAATGTACTCACAACCCA

TGTATTCGCATGTCAAGCGAACACGAATCTTTTTCTGTAATTATAATCTGCAAGCCAGTA

AATATCATCTTCATGGACCATCTATTCCAGCCCGATACCTAGAGTGTCTTTTGCCCCCCG

CCATGAGGACCATTGATTCGCCCACATGTTGCTCTTCGCCACTGCAGACTGCCAACGCCA

TCCATGATGCCCAGCCACTGTCTCGACTGCGCATCACGGGAATAACTACGCCTGATCTTT

TTTCCAGTCGCTGCTCCAGATGAACGTCCCGCATCTCCACTCCGTCCCCGATTCTTTGAC

TCCCGACTTCCCCAACGGGCGGGCACATACGTAGAAATGA

>ZH2

TCTTGATGATGTATCCCGACTACCGTTACACCCCTCGCAAGCCGTCTGAGAAGCGCCATC

GAAAGCCTAGTGGCCAAAGCAAGAAGACCAGCTTAGCAGCGTCGATGAGGTAGAGGCCGC

CTCCTACCCGAGAGCGACAAAACCATCAGCGCGCCGACTTGTCGGCTCCAGACGGAGGAG

TATGAGTTATACCTACCGACTGCGACAAGTCGCTGTAGTATCAGTCATGGAACGGAATAG

ATATCGGCGCTGCCAGACCACCTTAGGTCGTCCTTGTGTTCTAGACTGGCAGCATCAGCG

ACCAGAGGAGCCTCAGGACATGGCGCCTTTGGAGGCTGTCTTCGATCTCTATCCAGTCAG

CGTGACCCAGGAGGCGACCACGCATCTGGTCGTCGATTCACTTCCCGCATGCTGTGGCAA

GAGTTACCTGCGCGAGAAACCAACAGACCTTTGTGCGGCGATACTTAGACGTACGAACGC

TAGGAGATCACCGGATCTGAGGAACCAAGAATCATCCTGGACCATCTGCTTCGATGGCAA

CGCGAAGCAGAGATCTTCGATTTGATGAACATATTGGTGCAAAAATGTACTCACAACCCA

TGTATTCGCATGTCAAGCGAACACGAATCTTTTTCTGTAATTATAATCTGCAAGCCAGTA

AATATCATCTTCATGGACCATCTATTCCAGCCCGATACCTAGAGTGTCTTTTGCCCCCCG

CCATGAGGACCATTGATTCGCCCACATGTTGCTCTTCGCCACTGCAGACTGCCAACGCCA

TCCATGATGCCCAGCCACTGTCTCGACTGCGCATCACGGGAATAACTACGCCTGATCTTT

TTTCCAGTCGCTGCTCCAGATGAACGTCCCGCATCTCCACTCCGTCCCCGATTCTTTGAC

TCCCGACTTCCCCAACGGGCGGGCACATACGTAGAAATGA

>BM5

GAGCCAAAGAATCGGGGACGGAGTGGAGGTGCGGGACGTTCATCTGGAGCAGCGACTGGA

AAAAAGATCAGTACCTAGTTATTCCCGTGATGCGCAGTCGAGACAGTGGCTGGGCATCAT

GGATGGTGTTGGCAGTCTGCAGTGGCGAAGAGCAACATGTGGGCGAATCAATGGTCCTCA

CGGCGGGGGCCAAAAGACACTCTAGGTATCGGGCTGGAATAGATGGTCCATGAAGATGAT

ATTTACTGGCTTGCAGATTATAATTACAGAAAAAGATTCGTATTCGCTTGACATGCGAAT

ACATGGGTTGTGAGTACATTTTTGCACCAATATGTTCATCAAATCGAAGATCTCTGCTTC

GCGTTGCCATCGAAGCAGATGGTCCAGGATGATTCTTGGTTCCTCAGATCCGGTGATCTC

CTAGCGTTCGTACGTCTAATTATCGCCGCACAAAGGTCTGTTGGTTTCTCGCGCAGGTAA

CTCTTGCCACAGCATGCGGGAAGTGAATCGACGAACAGATGCGTGGTCGCCTCCTGGGTC

ACGCTGACTGGATAGAGATCGAAGACAGCCTCCAAAGGCGCCATGTTCTGAGGCTCCTCT

GGTCGCTGATGCTGCCAGTCTAGAACACAAGGACGACCTAAAGTCGTTTGGCAGCGCCGA

TATCTATTCCGTTCCATGACTGATACTACAGCGACTTGTCGCAGTCGGTAGGTATAACTC

ATACTCCTCCGTCTGGAGCCGACAAGTGGGCGCGCTGATGGTTTTGTCGCTCTCGGGTAG

GAGGCGGCCTCTACCTCATCGACGCTGCTAAGCTGGTCTTCTTGCTTTGGCCACTAGGCT

TTCGATGGCGCTTCTCAGACGGCTTGCGAGGGGTGTAACGGTAGTCGGGATACATCATCA

AGA

>BX1

GAGCCAAAGAATCGGGGACGGAGTGGAGGTGCGGGACGTTCATCTGGAGCAGCGACTGGA

AAAAAGATCAGTACCTAGTTATTCCCGTGATGCGCAGTCGAGACAGTGGCTGGGCATCAT

GGATGGTGTTGGCAGTCTGCAGTGGCGAAGAGCAACATGTGGGCGAATCAATGGTCCTCA

CGGCGGGGGCCAAAAGACACTCTAGGTATCGGGCTGGAATAGATGGTCCATGAAGATGAT

ATTTACTGGCTTGCAGATTATAATTACAGAAAAAGATTCGTATTCGCTTGACATGCGAAT

ACATGGGTTGTGAGTACATTTTTGCACCAATATGTTCATCAAATCGAAGATCTCTGCTTC

GCGTTGCCATCGAAGCAGATGGTCCAGGATGATTCTTGGTTCCTCAGATCCGGTGATCTC

CTAGCGTTCGTACGTCTAATTATCGCCGCACAAAGGTCTGTTGGTTTCTCGCGCAGGTAA

CTCTTGCCACAGCATGCGGGAAGTGAATCGACGAACAGATGCGTGGTCGCCTCCTGGGTC

ACGCTGACTGGATAGAGATCGAAGACAGCCTCCAAAGGCGCCATGTTCTGAGGCTCCTCT

GGTCGCTGATGCTGCCAGTCTAGAACACAAGGACGACCTAAAGTCGTTTGGCAGCGCCGA

TATCTATTCCGTTCCATGACTGATACTACAGCGACTTGTCGCAGTCGGTAGGTATAACTC

ATACTCCTCCGTCTGGAGCCGACAAGTGGGCGCGCTGATGGTTTTGTCGCTCTCGGGTAG

GAGGCGGCCTCTACCTCATCGACGCTGCTAAGCTGGTCTTCTTGCTTTGGCCACTAGGCT

TTCGATGGCGCTTCTCAGACGGCTTGCGAGGGGTGTAACGGTAGTCGGGATACATCATCA

AGA

>F5

GAGCCAAAGAATCGGGGACGGAGTGGAGGTGCGGGACGTTCATCTGGAGCAGCGACTGGA

AAAAAGATCAGTACCTAGTTATTCCCGTGATGCGCAGTCGAGACAGTGGCTGGGCATCAT

GGATGGTGTTGGCAGTCTGCAGTGGCGAAGAGCAACATGTGGGCGAATCAATGGTCCTCA

CGGCGGGGGCCAAAAGACACTCTAGGTATCGGGCTGGAATAGATGGTCCATGAAGATGAT

ATTTACTGGCTTGCAGATTATAATTACAGAAAAAGATTCGTATTCGCTTGACATGCGAAT

ACATGGGTTGTGAGTACATTTTTGCACCAATATGTTCATCAAATCGAAGATCTCTGCTTC

GCGTTGCCATCGAAGCAGATGGTCCAGGATGATTCTTGGTTCCTCAGATCCGGTGATCTC

CTAGCGTTCGTACGTCTAATTATCGCCGCACAAAGGTCTGTTGGTTTCTCGCGCAGGTAA

CTCTTGCCACAGCATGCGGGAAGTGAATCGACGAACAGATGCGTGGTCGCCTCCTGGGTC

ACGCTGACTGGATAGAGATCGAAGACAGCCTCCAAAGGCGCCATGTTCTGAGGCTCCTCT

GGTCGCTGATGCTGCCAGTCTAGAACACAAGGACGACCTAAAGTCGTTTGGCAGCGCCGA

TATCTATTCCGTTCCATGACTGATACTACAGCGACTTGTCGCAGTCGGTAGGTATAACTC

ATACTCCTCCGTCTGGAGCCGACAAGTGGGCGCGCTGATGGTTTTGTCGCTCTCGGGTAG

GAGGCGGCCTCTACCTCATCGACGCTGCTAAGCTGGTCTTCTTGCTTTGGCCACTAGGCT

TTCGATGGCGCTTCTCAGACGGCTTGCGAGGGGTGTAACGGTAGTCGGGATACATCATCA

AGA

>GX1

CGCCCGTTGGGGAAGTCGGGAGCCAAAGAATCGGGGACGGAGTGGAGGTGCGGGACATTC

ATCTGGAGCAGCGACCGGAAAAAAGATCAGACCTAGTTATTCCCGTGATGCGCAGTCGAG

ACAGTGGCTGGGCATCATGGATGGTGTTGGCAGTCTGCAGTGGCGAAGAGCAACATGTGG

GCGAATCAATGGTCCTCACGGCGGGGGCCAAAAGACACTCTAGGTATCGGGCTGGAATAG

ATGGTCCATGAAGATGATATTTACTGGCTTGCAGATTATAATTACAGAAAAAGATTCGTA

TTCGCTTGACATGCGAATACATGGGTTGTGAGTACATTTTTGCACCAATATGTTCATCAA

ATCGAAGATCTCTGCTTCGCGTTGCCATCGAAGCAGATGGTCCAGGATGATTCTTGGTTC

CTCAGATCCGGTGATCTCCTAGCGTTCGTACGTCTAATTATCGCCGCACAAAGGTCTGTT

GGTTTCTCGCGCAGGTAACTCTTGCCACAGCATGCGGGAAGTGAATCGACGAACAGATGC

GTGGTCGCCTCCTGGGTCACGCTGACTGGATAGAGATCGAAGACAGCCTCCAAAGGCGCC

ATGTTCTGAGGCTCCTCTGGTCGCTGATGCTGCCAGTCTAGAACACAAGGACGACCTAAA

GTCGTTTGGCAGCGCCGATATCTATTCCGTTCCATGACTGATACTACAGCGACTTGTCGC

AGTCGGTAGGTATAACTCATACTCCTCCGTCTGGAGCCGACAAGTGGGCGCGCTGATGGT

TTTGTCGCTCTCGGGTAGGAGGCGGCCTCTACCTCATCGACGCTGCTAAGCTGGTCTTCT

TGCTTTGGCCACTAGGCTTTCGATGGCGCTTCTCAGACGGCTTGCGAGGGGTGTAACGGT

AGTCGGGATACATCATCAAGAGGCGCTCCTTGTGCATCTTTGC

>GT7

CGCCCGTTGGGGAAGTCGGGAGCCAAAGAATCGGGGACGGAGTGGAGGTGCGGGACATTC

ATCTGGAGCAGCGACCGGAAAAAAGATCAGACCTAGTTATTCCCGTGATGCGCAGTCGAG

ACAGTGGCTGGGCATCATGGATGGTGTTGGCAGTCTGCAGTGGCGAAGAGCAACATGTGG

GCGAATCAATGGTCCTCACGGCGGGGGCCAAAAGACACTCTAGGTATCGGGCTGGAATAG

ATGGTCCATGAAGATGATATTTACTGGCTTGCAGATTATAATTACAGAAAAAGATTCGTA

TTCGCTTGACATGCGAATACATGGGTTGTGAGTACATTTTTGCACCAATATGTTCATCAA

ATCGAAGATCTCTGCTTCGCGTTGCCATCGAAGCAGATGGTCCAGGATGATTCTTGGTTC

CTCAGATCCGGTGATCTCCTAGCGTTCGTACGTCTAATTATCGCCGCACAAAGGTCTGTT

GGTTTCTCGCGCAGGTAACTCTTGCCACAGCATGCGGGAAGTGAATCGACGAACAGATGC

GTGGTCGCCTCCTGGGTCACGCTGACTGGATAGAGATCGAAGACAGCCTCCAAAGGCGCC

ATGTTCTGAGGCTCCTCTGGTCGCTGATGCTGCCAGTCTAGAACACAAGGACGACCTAAA

GTCGTTTGGCAGCGCCGATATCTATTCCGTTCCATGACTGATACTACAGCGACTTGTCGC

AGTCGGTAGGTATAACTCATACTCCTCCGTCTGGAGCCGACAAGTGGGCGCGCTGATGGT

TTTGTCGCTCTCGGGTAGGAGGCGGCCTCTACCTCATCGACGCTGCTAAGCTGGTCTTCT

TGCTTTGGCCACTAGGCTTTCGATGGCGCTTCTCAGACGGCTTGCGAGGGGTGTAACGGT

AGTCGGGATACATCATCAAGAGGCGCTCCTTGTGCATCTTTGC

>HC2

CGCCCGTTGGGGAAGTCGGGAGCCAAAGAATCGGGGACGGAGTGGAGGTGCGGGACATTC

ATCTGGAGCAGCGACCGGAAAAAAGATCAGACCTAGTTATTCCCGTGATGCGCAGTCGAG

ACAGTGGCTGGGCATCATGGATGGTGTTGGCAGTCTGCAGTGGCGAAGAGCAACATGTGG

GCGAATCAATGGTCCTCACGGCGGGGGCCAAAAGACACTCTAGGTATCGGGCTGGAATAG

ATGGTCCATGAAGATGATATTTACTGGCTTGCAGATTATAATTACAGAAAAAGATTCGTA

TTCGCTTGACATGCGAATACATGGGTTGTGAGTACATTTTTGCACCAATATGTTCATCAA

ATCGAAGATCTCTGCTTCGCGTTGCCATCGAAGCAGATGGTCCAGGATGATTCTTGGTTC

CTCAGATCCGGTGATCTCCTAGCGTTCGTACGTCTAATTATCGCCGCACAAAGGTCTGTT

GGTTTCTCGCGCAGGTAACTCTTGCCACAGCATGCGGGAAGTGAATCGACGAACAGATGC

GTGGTCGCCTCCTGGGTCACGCTGACTGGATAGAGATCGAAGACAGCCTCCAAAGGCGCC

ATGTTCTGAGGCTCCTCTGGTCGCTGATGCTGCCAGTCTAGAACACAAGGACGACCTAAA

GTCGTTTGGCAGCGCCGATATCTATTCCGTTCCATGACTGATACTACAGCGACTTGTCGC

AGTCGGTAGGTATAACTCATACTCCTCCGTCTGGAGCCGACAAGTGGGCGCGCTGATGGT

TTTGTCGCTCTCGGGTAGGAGGCGGCCTCTACCTCATCGACGCTGCTAAGCTGGTCTTCT

TGCTTTGGCCACTAGGCTTTCGATGGCGCTTCTCAGACGGCTTGCGAGGGGTGTAACGGT

AGTCGGGATACATCATCAAGAGGCGCTCCTTGTGCATCTTTGC

>HC6

CGCCCGTTGGGGAAGTCGGGAGCCAAAGAATCGGGGACGGAGTGGAGGTGCGGGACATTC

ATCTGGAGCAGCGACCGGAAAAAAGATCAGACCTAGTTATTCCCGTGATGCGCAGTCGAG

ACAGTGGCTGGGCATCATGGATGGTGTTGGCAGTCTGCAGTGGCGAAGAGCAACATGTGG

GCGAATCAATGGTCCTCACGGCGGGGGCCAAAAGACACTCTAGGTATCGGGCTGGAATAG

ATGGTCCATGAAGATGATATTTACTGGCTTGCAGATTATAATTACAGAAAAAGATTCGTA

TTCGCTTGACATGCGAATACATGGGTTGTGAGTACATTTTTGCACCAATATGTTCATCAA

ATCGAAGATCTCTGCTTCGCGTTGCCATCGAAGCAGATGGTCCAGGATGATTCTTGGTTC

CTCAGATCCGGTGATCTCCTAGCGTTCGTACGTCTAATTATCGCCGCACAAAGGTCTGTT

GGTTTCTCGCGCAGGTAACTCTTGCCACAGCATGCGGGAAGTGAATCGACGAACAGATGC

GTGGTCGCCTCCTGGGTCACGCTGACTGGATAGAGATCGAAGACAGCCTCCAAAGGCGCC

ATGTTCTGAGGCTCCTCTGGTCGCTGATGCTGCCAGTCTAGAACACAAGGACGACCTAAA

GTCGTTTGGCAGCGCCGATATCTATTCCGTTCCATGACTGATACTACAGCGACTTGTCGC

AGTCGGTAGGTATAACTCATACTCCTCCGTCTGGAGCCGACAAGTGGGCGCGCTGATGGT

TTTGTCGCTCTCGGGTAGGAGGCGGCCTCTACCTCATCGACGCTGCTAAGCTGGTCTTCT

TGCTTTGGCCACTAGGCTTTCGATGGCGCTTCTCAGACGGCTTGCGAGGGGTGTAACGGT

AGTCGGGATACATCATCAAGAGGCGCTCCTTGTGCATCTTTGC

>JS3

CGCCCGTTGGGGAAGTCGGGAGCCAAAGAATCGGGGACGGAGTGGAGGTGCGGGACATTC

ATCTGGAGCAGCGACCGGAAAAAAGATCAGACCTAGTTATTCCCGTGATGCGCAGTCGAG

ACAGTGGCTGGGCATCATGGATGGTGTTGGCAGTCTGCAGTGGCGAAGAGCAACATGTGG

GCGAATCAATGGTCCTCACGGCGGGGGCCAAAAGACACTCTAGGTATCGGGCTGGAATAG

ATGGTCCATGAAGATGATATTTACTGGCTTGCAGATTATAATTACAGAAAAAGATTCGTA

TTCGCTTGACATGCGAATACATGGGTTGTGAGTACATTTTTGCACCAATATGTTCATCAA

ATCGAAGATCTCTGCTTCGCGTTGCCATCGAAGCAGATGGTCCAGGATGATTCTTGGTTC

CTCAGATCCGGTGATCTCCTAGCGTTCGTACGTCTAATTATCGCCGCACAAAGGTCTGTT

GGTTTCTCGCGCAGGTAACTCTTGCCACAGCATGCGGGAAGTGAATCGACGAACAGATGC

GTGGTCGCCTCCTGGGTCACGCTGACTGGATAGAGATCGAAGACAGCCTCCAAAGGCGCC

ATGTTCTGAGGCTCCTCTGGTCGCTGATGCTGCCAGTCTAGAACACAAGGACGACCTAAA

GTCGTTTGGCAGCGCCGATATCTATTCCGTTCCATGACTGATACTACAGCGACTTGTCGC

AGTCGGTAGGTATAACTCATACTCCTCCGTCTGGAGCCGACAAGTGGGCGCGCTGATGGT

TTTGTCGCTCTCGGGTAGGAGGCGGCCTCTACCTCATCGACGCTGCTAAGCTGGTCTTCT

TGCTTTGGCCACTAGGCTTTCGATGGCGCTTCTCAGACGGCTTGCGAGGGGTGTAACGGT

AGTCGGGATACATCATCAAGAGGCGCTCCTTGTGCATCTTTGC

>JS9

CGCCCGTTGGGGAAGTCGGGAGCCAAAGAATCGGGGACGGAGTGGAGGTGCGGGACATTC

ATCTGGAGCAGCGACCGGAAAAAAGATCAGACCTAGTTATTCCCGTGATGCGCAGTCGAG

ACAGTGGCTGGGCATCATGGATGGTGTTGGCAGTCTGCAGTGGCGAAGAGCAACATGTGG

GCGAATCAATGGTCCTCACGGCGGGGGCCAAAAGACACTCTAGGTATCGGGCTGGAATAG

ATGGTCCATGAAGATGATATTTACTGGCTTGCAGATTATAATTACAGAAAAAGATTCGTA

TTCGCTTGACATGCGAATACATGGGTTGTGAGTACATTTTTGCACCAATATGTTCATCAA

ATCGAAGATCTCTGCTTCGCGTTGCCATCGAAGCAGATGGTCCAGGATGATTCTTGGTTC

CTCAGATCCGGTGATCTCCTAGCGTTCGTACGTCTAATTATCGCCGCACAAAGGTCTGTT

GGTTTCTCGCGCAGGTAACTCTTGCCACAGCATGCGGGAAGTGAATCGACGAACAGATGC

GTGGTCGCCTCCTGGGTCACGCTGACTGGATAGAGATCGAAGACAGCCTCCAAAGGCGCC

ATGTTCTGAGGCTCCTCTGGTCGCTGATGCTGCCAGTCTAGAACACAAGGACGACCTAAA

GTCGTTTGGCAGCGCCGATATCTATTCCGTTCCATGACTGATACTACAGCGACTTGTCGC

AGTCGGTAGGTATAACTCATACTCCTCCGTCTGGAGCCGACAAGTGGGCGCGCTGATGGT

TTTGTCGCTCTCGGGTAGGAGGCGGCCTCTACCTCATCGACGCTGCTAAGCTGGTCTTCT

TGCTTTGGCCACTAGGCTTTCGATGGCGCTTCTCAGACGGCTTGCGAGGGGTGTAACGGT

AGTCGGGATACATCATCAAGAGGCGCTCCTTGTGCATCTTTGC

>LC7

CGCCCGTTGGGGAAGTCGGGAGCCAAAGAATCGGGGACGGAGTGGAGGTGCGGGACATTC

ATCTGGAGCAGCGACCGGAAAAAAGATCAGACCTAGTTATTCCCGTGATGCGCAGTCGAG

ACAGTGGCTGGGCATCATGGATGGTGTTGGCAGTCTGCAGTGGCGAAGAGCAACATGTGG

GCGAATCAATGGTCCTCACGGCGGGGGCCAAAAGACACTCTAGGTATCGGGCTGGAATAG

ATGGTCCATGAAGATGATATTTACTGGCTTGCAGATTATAATTACAGAAAAAGATTCGTA

TTCGCTTGACATGCGAATACATGGGTTGTGAGTACATTTTTGCACCAATATGTTCATCAA

ATCGAAGATCTCTGCTTCGCGTTGCCATCGAAGCAGATGGTCCAGGATGATTCTTGGTTC

CTCAGATCCGGTGATCTCCTAGCGTTCGTACGTCTAATTATCGCCGCACAAAGGTCTGTT

GGTTTCTCGCGCAGGTAACTCTTGCCACAGCATGCGGGAAGTGAATCGACGAACAGATGC

GTGGTCGCCTCCTGGGTCACGCTGACTGGATAGAGATCGAAGACAGCCTCCAAAGGCGCC

ATGTTCTGAGGCTCCTCTGGTCGCTGATGCTGCCAGTCTAGAACACAAGGACGACCTAAA

GTCGTTTGGCAGCGCCGATATCTATTCCGTTCCATGACTGATACTACAGCGACTTGTCGC

AGTCGGTAGGTATAACTCATACTCCTCCGTCTGGAGCCGACAAGTGGGCGCGCTGATGGT

TTTGTCGCTCTCGGGTAGGAGGCGGCCTCTACCTCATCGACGCTGCTAAGCTGGTCTTCT

TGCTTTGGCCACTAGGCTTTCGATGGCGCTTCTCAGACGGCTTGCGAGGGGTGTAACGGT

AGTCGGGATACATCATCAAGAGGCGCTCCTTGTGCATCTTTGC

>LG2

CGCCCGTTGGGGAAGTCGGGAGCCAAAGAATCGGGGACGGAGTGGAGGTGCGGGACATTC

ATCTGGAGCAGCGACCGGAAAAAAGATCAGACCTAGTTATTCCCGTGATGCGCAGTCGAG

ACAGTGGCTGGGCATCATGGATGGTGTTGGCAGTCTGCAGTGGCGAAGAGCAACATGTGG

GCGAATCAATGGTCCTCACGGCGGGGGCCAAAAGACACTCTAGGTATCGGGCTGGAATAG

ATGGTCCATGAAGATGATATTTACTGGCTTGCAGATTATAATTACAGAAAAAGATTCGTA

TTCGCTTGACATGCGAATACATGGGTTGTGAGTACATTTTTGCACCAATATGTTCATCAA

ATCGAAGATCTCTGCTTCGCGTTGCCATCGAAGCAGATGGTCCAGGATGATTCTTGGTTC

CTCAGATCCGGTGATCTCCTAGCGTTCGTACGTCTAATTATCGCCGCACAAAGGTCTGTT

GGTTTCTCGCGCAGGTAACTCTTGCCACAGCATGCGGGAAGTGAATCGACGAACAGATGC

GTGGTCGCCTCCTGGGTCACGCTGACTGGATAGAGATCGAAGACAGCCTCCAAAGGCGCC

ATGTTCTGAGGCTCCTCTGGTCGCTGATGCTGCCAGTCTAGAACACAAGGACGACCTAAA

GTCGTTTGGCAGCGCCGATATCTATTCCGTTCCATGACTGATACTACAGCGACTTGTCGC

AGTCGGTAGGTATAACTCATACTCCTCCGTCTGGAGCCGACAAGTGGGCGCGCTGATGGT

TTTGTCGCTCTCGGGTAGGAGGCGGCCTCTACCTCATCGACGCTGCTAAGCTGGTCTTCT

TGCTTTGGCCACTAGGCTTTCGATGGCGCTTCTCAGACGGCTTGCGAGGGGTGTAACGGT

AGTCGGGATACATCATCAAGAGGCGCTCCTTGTGCATCTTTGC

>LG4

CGCCCGTTGGGGAAGTCGGGAGCCAAAGAATCGGGGACGGAGTGGAGGTGCGGGACATTC

ATCTGGAGCAGCGACCGGAAAAAAGATCAGACCTAGTTATTCCCGTGATGCGCAGTCGAG

ACAGTGGCTGGGCATCATGGATGGTGTTGGCAGTCTGCAGTGGCGAAGAGCAACATGTGG

GCGAATCAATGGTCCTCACGGCGGGGGCCAAAAGACACTCTAGGTATCGGGCTGGAATAG

ATGGTCCATGAAGATGATATTTACTGGCTTGCAGATTATAATTACAGAAAAAGATTCGTA

TTCGCTTGACATGCGAATACATGGGTTGTGAGTACATTTTTGCACCAATATGTTCATCAA

ATCGAAGATCTCTGCTTCGCGTTGCCATCGAAGCAGATGGTCCAGGATGATTCTTGGTTC

CTCAGATCCGGTGATCTCCTAGCGTTCGTACGTCTAATTATCGCCGCACAAAGGTCTGTT

GGTTTCTCGCGCAGGTAACTCTTGCCACAGCATGCGGGAAGTGAATCGACGAACAGATGC

GTGGTCGCCTCCTGGGTCACGCTGACTGGATAGAGATCGAAGACAGCCTCCAAAGGCGCC

ATGTTCTGAGGCTCCTCTGGTCGCTGATGCTGCCAGTCTAGAACACAAGGACGACCTAAA

GTCGTTTGGCAGCGCCGATATCTATTCCGTTCCATGACTGATACTACAGCGACTTGTCGC

AGTCGGTAGGTATAACTCATACTCCTCCGTCTGGAGCCGACAAGTGGGCGCGCTGATGGT

TTTGTCGCTCTCGGGTAGGAGGCGGCCTCTACCTCATCGACGCTGCTAAGCTGGTCTTCT

TGCTTTGGCCACTAGGCTTTCGATGGCGCTTCTCAGACGGCTTGCGAGGGGTGTAACGGT

AGTCGGGATACATCATCAAGAGGCGCTCCTTGTGCATCTTTGC

>LV2

CGCCCGTTGGGGAAGTCGGGAGCCAAAGAATCGGGGACGGAGTGGAGGTGCGGGACATTC

ATCTGGAGCAGCGACCGGAAAAAAGATCAGACCTAGTTATTCCCGTGATGCGCAGTCGAG

ACAGTGGCTGGGCATCATGGATGGTGTTGGCAGTCTGCAGTGGCGAAGAGCAACATGTGG

GCGAATCAATGGTCCTCACGGCGGGGGCCAAAAGACACTCTAGGTATCGGGCTGGAATAG

ATGGTCCATGAAGATGATATTTACTGGCTTGCAGATTATAATTACAGAAAAAGATTCGTA

TTCGCTTGACATGCGAATACATGGGTTGTGAGTACATTTTTGCACCAATATGTTCATCAA

ATCGAAGATCTCTGCTTCGCGTTGCCATCGAAGCAGATGGTCCAGGATGATTCTTGGTTC

CTCAGATCCGGTGATCTCCTAGCGTTCGTACGTCTAATTATCGCCGCACAAAGGTCTGTT

GGTTTCTCGCGCAGGTAACTCTTGCCACAGCATGCGGGAAGTGAATCGACGAACAGATGC

GTGGTCGCCTCCTGGGTCACGCTGACTGGATAGAGATCGAAGACAGCCTCCAAAGGCGCC

ATGTTCTGAGGCTCCTCTGGTCGCTGATGCTGCCAGTCTAGAACACAAGGACGACCTAAA

GTCGTTTGGCAGCGCCGATATCTATTCCGTTCCATGACTGATACTACAGCGACTTGTCGC

AGTCGGTAGGTATAACTCATACTCCTCCGTCTGGAGCCGACAAGTGGGCGCGCTGATGGT

TTTGTCGCTCTCGGGTAGGAGGCGGCCTCTACCTCATCGACGCTGCTAAGCTGGTCTTCT

TGCTTTGGCCACTAGGCTTTCGATGGCGCTTCTCAGACGGCTTGCGAGGGGTGTAACGGT

AGTCGGGATACATCATCAAGAGGCGCTCCTTGTGCATCTTTGC

>NC25

GAGCCAAAGAATCGGGGACGGAGTGGAGGTGCGGGACGTTCATCTGGAGCAGCGACTGGA

AAAAAGATCAGTACCTAGTTATTCCCGTGATGCGCAGTCGAGACAGTGGCTGGGCATCAT

GGATGGTGTTGGCAGTCTGCAGTGGCGAAGAGCAACATGTGGGCGAATCAATGGTCCTCA

CGGCGGGGGCCAAAAGACACTCTAGGTATCGGGCTGGAATAGATGGTCCATGAAGATGAT

ATTTACTGGCTTGCAGATTATAATTACAGAAAAAGATTCGTATTCGCTTGACATGCGAAT

ACATGGGTTGTGAGTACATTTTTGCACCAATATGTTCATCAAATCGAAGATCTCTGCTTC

GCGTTGCCATCGAAGCAGATGGTCCAGGATGATTCTTGGTTCCTCAGATCCGGTGATCTC

CTAGCGTTCGTACGTCTAATTATCGCCGCACAAAGGTCTGTTGGTTTCTCGCGCAGGTAA

CTCTTGCCACAGCATGCGGGAAGTGAATCGACGAACAGATGCGTGGTCGCCTCCTGGGTC

ACGCTGACTGGATAGAGATCGAAGACAGCCTCCAAAGGCGCCATGTTCTGAGGCTCCTCT

GGTCGCTGATGCTGCCAGTCTAGAACACAAGGACGACCTAAAGTCGTTTGGCAGCGCCGA

TATCTATTCCGTTCCATGACTGATACTACAGCGACTTGTCGCAGTCGGTAGGTATAACTC

ATACTCCTCCGTCTGGAGCCGACAAGTGGGCGCGCTGATGGTTTTGTCGCTCTCGGGTAG

GAGGCGGCCTCTACCTCATCGACGCTGCTAAGCTGGTCTTCTTGCTTTGGCCACTAGGCT

TTCGATGGCGCTTCTCAGACGGCTTGCGAGGGGTGTAACGGTAGTCGGGATACATCATCA

AGA

>NC26

GAGCCAAAGAATCGGGGACGGAGTGGAGGTGCGGGACGTTCATCTGGAGCAGCGACTGGA

AAAAAGATCAGTACCTAGTTATTCCCGTGATGCGCAGTCGAGACAGTGGCTGGGCATCAT

GGATGGTGTTGGCAGTCTGCAGTGGCGAAGAGCAACATGTGGGCGAATCAATGGTCCTCA

CGGCGGGGGCCAAAAGACACTCTAGGTATCGGGCTGGAATAGATGGTCCATGAAGATGAT

ATTTACTGGCTTGCAGATTATAATTACAGAAAAAGATTCGTATTCGCTTGACATGCGAAT

ACATGGGTTGTGAGTACATTTTTGCACCAATATGTTCATCAAATCGAAGATCTCTGCTTC

GCGTTGCCATCGAAGCAGATGGTCCAGGATGATTCTTGGTTCCTCAGATCCGGTGATCTC

CTAGCGTTCGTACGTCTAATTATCGCCGCACAAAGGTCTGTTGGTTTCTCGCGCAGGTAA

CTCTTGCCACAGCATGCGGGAAGTGAATCGACGAACAGATGCGTGGTCGCCTCCTGGGTC

ACGCTGACTGGATAGAGATCGAAGACAGCCTCCAAAGGCGCCATGTTCTGAGGCTCCTCT

GGTCGCTGATGCTGCCAGTCTAGAACACAAGGACGACCTAAAGTCGTTTGGCAGCGCCGA

TATCTATTCCGTTCCATGACTGATACTACAGCGACTTGTCGCAGTCGGTAGGTATAACTC

ATACTCCTCCGTCTGGAGCCGACAAGTGGGCGCGCTGATGGTTTTGTCGCTCTCGGGTAG

GAGGCGGCCTCTACCTCATCGACGCTGCTAAGCTGGTCTTCTTGCTTTGGCCACTAGGCT

TTCGATGGCGCTTCTCAGACGGCTTGCGAGGGGTGTAACGGTAGTCGGGATACATCATCA

AGA

>PL2

CGCCCGTTGGGGAAGTCGGGAGCCAAAGAATCGGGGACGGAGTGGAGGTGCGGGACATTC

ATCTGGAGCAGCGACCGGAAAAAAGATCAGACCTAGTTATTCCCGTGATGCGCAGTCGAG

ACAGTGGCTGGGCATCATGGATGGTGTTGGCAGTCTGCAGTGGCGAAGAGCAACATGTGG

GCGAATCAATGGTCCTCACGGCGGGGGCCAAAAGACACTCTAGGTATCGGGCTGGAATAG

ATGGTCCATGAAGATGATATTTACTGGCTTGCAGATTATAATTACAGAAAAAGATTCGTA

TTCGCTTGACATGCGAATACATGGGTTGTGAGTACATTTTTGCACCAATATGTTCATCAA

ATCGAAGATCTCTGCTTCGCGTTGCCATCGAAGCAGATGGTCCAGGATGATTCTTGGTTC

CTCAGATCCGGTGATCTCCTAGCGTTCGTACGTCTAATTATCGCCGCACAAAGGTCTGTT

GGTTTCTCGCGCAGGTAACTCTTGCCACAGCATGCGGGAAGTGAATCGACGAACAGATGC

GTGGTCGCCTCCTGGGTCACGCTGACTGGATAGAGATCGAAGACAGCCTCCAAAGGCGCC

ATGTTCTGAGGCTCCTCTGGTCGCTGATGCTGCCAGTCTAGAACACAAGGACGACCTAAA

GTCGTTTGGCAGCGCCGATATCTATTCCGTTCCATGACTGATACTACAGCGACTTGTCGC

AGTCGGTAGGTATAACTCATACTCCTCCGTCTGGAGCCGACAAGTGGGCGCGCTGATGGT

TTTGTCGCTCTCGGGTAGGAGGCGGCCTCTACCTCATCGACGCTGCTAAGCTGGTCTTCT

TGCTTTGGCCACTAGGCTTTCGATGGCGCTTCTCAGACGGCTTGCGAGGGGTGTAACGGT

AGTCGGGATACATCATCAAGAGGCGCTCCTTGTGCATCTTTGC

>PX3

CGCCCGTTGGGGAAGTCGGGAGCCAAAGAATCGGGGACGGAGTGGAGGTGCGGGACATTC

ATCTGGAGCAGCGACCGGAAAAAAGATCAGACCTAGTTATTCCCGTGATGCGCAGTCGAG

ACAGTGGCTGGGCATCATGGATGGTGTTGGCAGTCTGCAGTGGCGAAGAGCAACATGTGG

GCGAATCAATGGTCCTCACGGCGGGGGCCAAAAGACACTCTAGGTATCGGGCTGGAATAG

ATGGTCCATGAAGATGATATTTACTGGCTTGCAGATTATAATTACAGAAAAAGATTCGTA

TTCGCTTGACATGCGAATACATGGGTTGTGAGTACATTTTTGCACCAATATGTTCATCAA

ATCGAAGATCTCTGCTTCGCGTTGCCATCGAAGCAGATGGTCCAGGATGATTCTTGGTTC

CTCAGATCCGGTGATCTCCTAGCGTTCGTACGTCTAATTATCGCCGCACAAAGGTCTGTT

GGTTTCTCGCGCAGGTAACTCTTGCCACAGCATGCGGGAAGTGAATCGACGAACAGATGC

GTGGTCGCCTCCTGGGTCACGCTGACTGGATAGAGATCGAAGACAGCCTCCAAAGGCGCC

ATGTTCTGAGGCTCCTCTGGTCGCTGATGCTGCCAGTCTAGAACACAAGGACGACCTAAA

GTCGTTTGGCAGCGCCGATATCTATTCCGTTCCATGACTGATACTACAGCGACTTGTCGC

AGTCGGTAGGTATAACTCATACTCCTCCGTCTGGAGCCGACAAGTGGGCGCGCTGATGGT

TTTGTCGCTCTCGGGTAGGAGGCGGCCTCTACCTCATCGACGCTGCTAAGCTGGTCTTCT

TGCTTTGGCCACTAGGCTTTCGATGGCGCTTCTCAGACGGCTTGCGAGGGGTGTAACGGT

AGTCGGGATACATCATCAAGAGGCGCTCCTTGTGCATCTTTGC

>SC6

CGCCCGTTGGGGAAGTCGGGAGCCAAAGAATCGGGGACGGAGTGGAGGTGCGGGACATTC

ATCTGGAGCAGCGACCGGAAAAAAGATCAGACCTAGTTATTCCCGTGATGCGCAGTCGAG

ACAGTGGCTGGGCATCATGGATGGTGTTGGCAGTCTGCAGTGGCGAAGAGCAACATGTGG

GCGAATCAATGGTCCTCACGGCGGGGGCCAAAAGACACTCTAGGTATCGGGCTGGAATAG

ATGGTCCATGAAGATGATATTTACTGGCTTGCAGATTATAATTACAGAAAAAGATTCGTA

TTCGCTTGACATGCGAATACATGGGTTGTGAGTACATTTTTGCACCAATATGTTCATCAA

ATCGAAGATCTCTGCTTCGCGTTGCCATCGAAGCAGATGGTCCAGGATGATTCTTGGTTC

CTCAGATCCGGTGATCTCCTAGCGTTCGTACGTCTAATTATCGCCGCACAAAGGTCTGTT

GGTTTCTCGCGCAGGTAACTCTTGCCACAGCATGCGGGAAGTGAATCGACGAACAGATGC

GTGGTCGCCTCCTGGGTCACGCTGACTGGATAGAGATCGAAGACAGCCTCCAAAGGCGCC

ATGTTCTGAGGCTCCTCTGGTCGCTGATGCTGCCAGTCTAGAACACAAGGACGACCTAAA

GTCGTTTGGCAGCGCCGATATCTATTCCGTTCCATGACTGATACTACAGCGACTTGTCGC

AGTCGGTAGGTATAACTCATACTCCTCCGTCTGGAGCCGACAAGTGGGCGCGCTGATGGT

TTTGTCGCTCTCGGGTAGGAGGCGGCCTCTACCTCATCGACGCTGCTAAGCTGGTCTTCT

TGCTTTGGCCACTAGGCTTTCGATGGCGCTTCTCAGACGGCTTGCGAGGGGTGTAACGGT

AGTCGGGATACATCATCAAGAGGCGCTCCTTGTGCATCTTTGC

>SC9

CGCCCGTTGGGGAAGTCGGGAGCCAAAGAATCGGGGACGGAGTGGAGGTGCGGGACATTC

ATCTGGAGCAGCGACCGGAAAAAAGATCAGACCTAGTTATTCCCGTGATGCGCAGTCGAG

ACAGTGGCTGGGCATCATGGATGGTGTTGGCAGTCTGCAGTGGCGAAGAGCAACATGTGG

GCGAATCAATGGTCCTCACGGCGGGGGCCAAAAGACACTCTAGGTATCGGGCTGGAATAG

ATGGTCCATGAAGATGATATTTACTGGCTTGCAGATTATAATTACAGAAAAAGATTCGTA

TTCGCTTGACATGCGAATACATGGGTTGTGAGTACATTTTTGCACCAATATGTTCATCAA

ATCGAAGATCTCTGCTTCGCGTTGCCATCGAAGCAGATGGTCCAGGATGATTCTTGGTTC

CTCAGATCCGGTGATCTCCTAGCGTTCGTACGTCTAATTATCGCCGCACAAAGGTCTGTT

GGTTTCTCGCGCAGGTAACTCTTGCCACAGCATGCGGGAAGTGAATCGACGAACAGATGC

GTGGTCGCCTCCTGGGTCACGCTGACTGGATAGAGATCGAAGACAGCCTCCAAAGGCGCC

ATGTTCTGAGGCTCCTCTGGTCGCTGATGCTGCCAGTCTAGAACACAAGGACGACCTAAA

GTCGTTTGGCAGCGCCGATATCTATTCCGTTCCATGACTGATACTACAGCGACTTGTCGC

AGTCGGTAGGTATAACTCATACTCCTCCGTCTGGAGCCGACAAGTGGGCGCGCTGATGGT

TTTGTCGCTCTCGGGTAGGAGGCGGCCTCTACCTCATCGACGCTGCTAAGCTGGTCTTCT

TGCTTTGGCCACTAGGCTTTCGATGGCGCTTCTCAGACGGCTTGCGAGGGGTGTAACGGT

AGTCGGGATACATCATCAAGAGGCGCTCCTTGTGCATCTTTGC

>T5

CGCCCGTTGGGGAAGTCGGGAGCCAAAGAATCGGGGACGGAGTGGAGGTGCGGGACATTC

ATCTGGAGCAGCGACCGGAAAAAAGATCAGACCTAGTTATTCCCGTGATGCGCAGTCGAG

ACAGTGGCTGGGCATCATGGATGGTGTTGGCAGTCTGCAGTGGCGAAGAGCAACATGTGG

GCGAATCAATGGTCCTCACGGCGGGGGCCAAAAGACACTCTAGGTATCGGGCTGGAATAG

ATGGTCCATGAAGATGATATTTACTGGCTTGCAGATTATAATTACAGAAAAAGATTCGTA

TTCGCTTGACATGCGAATACATGGGTTGTGAGTACATTTTTGCACCAATATGTTCATCAA

ATCGAAGATCTCTGCTTCGCGTTGCCATCGAAGCAGATGGTCCAGGATGATTCTTGGTTC

CTCAGATCCGGTGATCTCCTAGCGTTCGTACGTCTAATTATCGCCGCACAAAGGTCTGTT

GGTTTCTCGCGCAGGTAACTCTTGCCACAGCATGCGGGAAGTGAATCGACGAACAGATGC

GTGGTCGCCTCCTGGGTCACGCTGACTGGATAGAGATCGAAGACAGCCTCCAAAGGCGCC

ATGTTCTGAGGCTCCTCTGGTCGCTGATGCTGCCAGTCTAGAACACAAGGACGACCTAAA

GTCGTTTGGCAGCGCCGATATCTATTCCGTTCCATGACTGATACTACAGCGACTTGTCGC

AGTCGGTAGGTATAACTCATACTCCTCCGTCTGGAGCCGACAAGTGGGCGCGCTGATGGT

TTTGTCGCTCTCGGGTAGGAGGCGGCCTCTACCTCATCGACGCTGCTAAGCTGGTCTTCT

TGCTTTGGCCACTAGGCTTTCGATGGCGCTTCTCAGACGGCTTGCGAGGGGTGTAACGGT

AGTCGGGATACATCATCAAGAGGCGCTCCTTGTGCATCTTTGC

>T9

CGCCCGTTGGGGAAGTCGGGAGCCAAAGAATCGGGGACGGAGTGGAGGTGCGGGACATTC

ATCTGGAGCAGCGACCGGAAAAAAGATCAGACCTAGTTATTCCCGTGATGCGCAGTCGAG

ACAGTGGCTGGGCATCATGGATGGTGTTGGCAGTCTGCAGTGGCGAAGAGCAACATGTGG

GCGAATCAATGGTCCTCACGGCGGGGGCCAAAAGACACTCTAGGTATCGGGCTGGAATAG

ATGGTCCATGAAGATGATATTTACTGGCTTGCAGATTATAATTACAGAAAAAGATTCGTA

TTCGCTTGACATGCGAATACATGGGTTGTGAGTACATTTTTGCACCAATATGTTCATCAA

ATCGAAGATCTCTGCTTCGCGTTGCCATCGAAGCAGATGGTCCAGGATGATTCTTGGTTC

CTCAGATCCGGTGATCTCCTAGCGTTCGTACGTCTAATTATCGCCGCACAAAGGTCTGTT

GGTTTCTCGCGCAGGTAACTCTTGCCACAGCATGCGGGAAGTGAATCGACGAACAGATGC

GTGGTCGCCTCCTGGGTCACGCTGACTGGATAGAGATCGAAGACAGCCTCCAAAGGCGCC

ATGTTCTGAGGCTCCTCTGGTCGCTGATGCTGCCAGTCTAGAACACAAGGACGACCTAAA

GTCGTTTGGCAGCGCCGATATCTATTCCGTTCCATGACTGATACTACAGCGACTTGTCGC

AGTCGGTAGGTATAACTCATACTCCTCCGTCTGGAGCCGACAAGTGGGCGCGCTGATGGT

TTTGTCGCTCTCGGGTAGGAGGCGGCCTCTACCTCATCGACGCTGCTAAGCTGGTCTTCT

TGCTTTGGCCACTAGGCTTTCGATGGCGCTTCTCAGACGGCTTGCGAGGGGTGTAACGGT

AGTCGGGATACATCATCAAGAGGCGCTCCTTGTGCATCTTTGC

>H3

GAGCCAAAGAATCGGGGACGGAGTGGAGGTGCGGGACGTTCATCTGGAGCAGCGACTGGA

AAAAAGATCAGTACCTAGTTATTCCCGTGATGCGCAGTCGAGACAGTGGCTGGGCATCAT

GGATGGTGTTGGCAGTCTGCAGTGGCGAAGAGCAACATGTGGGCGAATCAATGGTCCTCA

CGGCGGGGGCCAAAAGACACTCTAGGTATCGGGCTGGAATAGATGGTCCATGAAGATGAT

ATTTACTGGCTTGCAGATTATAATTACAGAAAAAGATTCGTATTCGCTTGACATGCGAAT

ACATGGGTTGTGAGTACATTTTTGCACCAATATGTTCATCAAATCGAAGATCTCTGCTTC

GCGTTGCCATCGAAGCAGATGGTCCAGGATGATTCTTGGTTCCTCAGATCCGGTGATCTC

CTAGCGTTCGTACGTCTAATTATCGCCGCACAAAGGTCTGTTGGTTTCTCGCGCAGGTAA

CTCTTGCCACAGCATGCGGGAAGTGAATCGACGAACAGATGCGTGGTCGCCTCCTGGGTC

ACGCTGACTGGATAGAGATCGAAGACAGCCTCCAAAGGCGCCATGTTCTGAGGCTCCTCT

GGTCGCTGATGCTGCCAGTCTAGAACACAAGGACGACCTAAAGTCGTTTGGCAGCGCCGA

TATCTATTCCGTTCCATGACTGATACTACAGCGACTTGTCGCAGTCGGTAGGTATAACTC

ATACTCCTCCGTCTGGAGCCGACAAGTGGGCGCGCTGATGGTTTTGTCGCTCTCGGGTAG

GAGGCGGCCTCTACCTCATCGACGCTGCTAAGCTGGTCTTCTTGCTTTGGCCACTAGGCT

TTCGATGGCGCTTCTCAGACGGCTTGCGAGGGGTGTAACGGTAGTCGGGATACATCATCA

AGA

>H4

GAGCCAAAGAATCGGGGACGGAGTGGAGGTGCGGGACGTTCATCTGGAGCAGCGACTGGA

AAAAAGATCAGTACCTAGTTATTCCCGTGATGCGCAGTCGAGACAGTGGCTGGGCATCAT

GGATGGTGTTGGCAGTCTGCAGTGGCGAAGAGCAACATGTGGGCGAATCAATGGTCCTCA

CGGCGGGGGCCAAAAGACACTCTAGGTATCGGGCTGGAATAGATGGTCCATGAAGATGAT

ATTTACTGGCTTGCAGATTATAATTACAGAAAAAGATTCGTATTCGCTTGACATGCGAAT

ACATGGGTTGTGAGTACATTTTTGCACCAATATGTTCATCAAATCGAAGATCTCTGCTTC

GCGTTGCCATCGAAGCAGATGGTCCAGGATGATTCTTGGTTCCTCAGATCCGGTGATCTC

CTAGCGTTCGTACGTCTAATTATCGCCGCACAAAGGTCTGTTGGTTTCTCGCGCAGGTAA

CTCTTGCCACAGCATGCGGGAAGTGAATCGACGAACAGATGCGTGGTCGCCTCCTGGGTC

ACGCTGACTGGATAGAGATCGAAGACAGCCTCCAAAGGCGCCATGTTCTGAGGCTCCTCT

GGTCGCTGATGCTGCCAGTCTAGAACACAAGGACGACCTAAAGTCGTTTGGCAGCGCCGA

TATCTATTCCGTTCCATGACTGATACTACAGCGACTTGTCGCAGTCGGTAGGTATAACTC

ATACTCCTCCGTCTGGAGCCGACAAGTGGGCGCGCTGATGGTTTTGTCGCTCTCGGGTAG

GAGGCGGCCTCTACCTCATCGACGCTGCTAAGCTGGTCTTCTTGCTTTGGCCACTAGGCT

TTCGATGGCGCTTCTCAGACGGCTTGCGAGGGGTGTAACGGTAGTCGGGATACATCATCA

AGA

>YH6

CGCCCGTTGGGGAAGTCGGGAGCCAAAGAATCGGGGACGGAGTGGAGGTGCGGGACATTC

ATCTGGAGCAGCGACCGGAAAAAAGATCAGACCTAGTTATTCCCGTGATGCGCAGTCGAG

ACAGTGGCTGGGCATCATGGATGGTGTTGGCAGTCTGCAGTGGCGAAGAGCAACATGTGG

GCGAATCAATGGTCCTCACGGCGGGGGCCAAAAGACACTCTAGGTATCGGGCTGGAATAG

ATGGTCCATGAAGATGATATTTACTGGCTTGCAGATTATAATTACAGAAAAAGATTCGTA

TTCGCTTGACATGCGAATACATGGGTTGTGAGTACATTTTTGCACCAATATGTTCATCAA

ATCGAAGATCTCTGCTTCGCGTTGCCATCGAAGCAGATGGTCCAGGATGATTCTTGGTTC

CTCAGATCCGGTGATCTCCTAGCGTTCGTACGTCTAATTATCGCCGCACAAAGGTCTGTT

GGTTTCTCGCGCAGGTAACTCTTGCCACAGCATGCGGGAAGTGAATCGACGAACAGATGC

GTGGTCGCCTCCTGGGTCACGCTGACTGGATAGAGATCGAAGACAGCCTCCAAAGGCGCC

ATGTTCTGAGGCTCCTCTGGTCGCTGATGCTGCCAGTCTAGAACACAAGGACGACCTAAA

GTCGTTTGGCAGCGCCGATATCTATTCCGTTCCATGACTGATACTACAGCGACTTGTCGC

AGTCGGTAGGTATAACTCATACTCCTCCGTCTGGAGCCGACAAGTGGGCGCGCTGATGGT

TTTGTCGCTCTCGGGTAGGAGGCGGCCTCTACCTCATCGACGCTGCTAAGCTGGTCTTCT

TGCTTTGGCCACTAGGCTTTCGATGGCGCTTCTCAGACGGCTTGCGAGGGGTGTAACGGT

AGTCGGGATACATCATCAAGAGGCGCTCCTTGTGCATCTTTGC

>YH7

CGCCCGTTGGGGAAGTCGGGAGCCAAAGAATCGGGGACGGAGTGGAGGTGCGGGACATTC

ATCTGGAGCAGCGACCGGAAAAAAGATCAGACCTAGTTATTCCCGTGATGCGCAGTCGAG

ACAGTGGCTGGGCATCATGGATGGTGTTGGCAGTCTGCAGTGGCGAAGAGCAACATGTGG

GCGAATCAATGGTCCTCACGGCGGGGGCCAAAAGACACTCTAGGTATCGGGCTGGAATAG

ATGGTCCATGAAGATGATATTTACTGGCTTGCAGATTATAATTACAGAAAAAGATTCGTA

TTCGCTTGACATGCGAATACATGGGTTGTGAGTACATTTTTGCACCAATATGTTCATCAA

ATCGAAGATCTCTGCTTCGCGTTGCCATCGAAGCAGATGGTCCAGGATGATTCTTGGTTC

CTCAGATCCGGTGATCTCCTAGCGTTCGTACGTCTAATTATCGCCGCACAAAGGTCTGTT

GGTTTCTCGCGCAGGTAACTCTTGCCACAGCATGCGGGAAGTGAATCGACGAACAGATGC

GTGGTCGCCTCCTGGGTCACGCTGACTGGATAGAGATCGAAGACAGCCTCCAAAGGCGCC

ATGTTCTGAGGCTCCTCTGGTCGCTGATGCTGCCAGTCTAGAACACAAGGACGACCTAAA

GTCGTTTGGCAGCGCCGATATCTATTCCGTTCCATGACTGATACTACAGCGACTTGTCGC

AGTCGGTAGGTATAACTCATACTCCTCCGTCTGGAGCCGACAAGTGGGCGCGCTGATGGT

TTTGTCGCTCTCGGGTAGGAGGCGGCCTCTACCTCATCGACGCTGCTAAGCTGGTCTTCT

TGCTTTGGCCACTAGGCTTTCGATGGCGCTTCTCAGACGGCTTGCGAGGGGTGTAACGGT

AGTCGGGATACATCATCAAGAGGCGCTCCTTGTGCATCTTTGC

>YM2

CGCCCGTTGGGGAAGTCGGGAGCCAAAGAATCGGGGACGGAGTGGAGGTGCGGGACATTC

ATCTGGAGCAGCGACCGGAAAAAAGATCAGACCTAGTTATTCCCGTGATGCGCAGTCGAG

ACAGTGGCTGGGCATCATGGATGGTGTTGGCAGTCTGCAGTGGCGAAGAGCAACATGTGG

GCGAATCAATGGTCCTCACGGCGGGGGCCAAAAGACACTCTAGGTATCGGGCTGGAATAG

ATGGTCCATGAAGATGATATTTACTGGCTTGCAGATTATAATTACAGAAAAAGATTCGTA

TTCGCTTGACATGCGAATACATGGGTTGTGAGTACATTTTTGCACCAATATGTTCATCAA

ATCGAAGATCTCTGCTTCGCGTTGCCATCGAAGCAGATGGTCCAGGATGATTCTTGGTTC

CTCAGATCCGGTGATCTCCTAGCGTTCGTACGTCTAATTATCGCCGCACAAAGGTCTGTT

GGTTTCTCGCGCAGGTAACTCTTGCCACAGCATGCGGGAAGTGAATCGACGAACAGATGC

GTGGTCGCCTCCTGGGTCACGCTGACTGGATAGAGATCGAAGACAGCCTCCAAAGGCGCC

ATGTTCTGAGGCTCCTCTGGTCGCTGATGCTGCCAGTCTAGAACACAAGGACGACCTAAA

GTCGTTTGGCAGCGCCGATATCTATTCCGTTCCATGACTGATACTACAGCGACTTGTCGC

AGTCGGTAGGTATAACTCATACTCCTCCGTCTGGAGCCGACAAGTGGGCGCGCTGATGGT

TTTGTCGCTCTCGGGTAGGAGGCGGCCTCTACCTCATCGACGCTGCTAAGCTGGTCTTCT

TGCTTTGGCCACTAGGCTTTCGATGGCGCTTCTCAGACGGCTTGCGAGGGGTGTAACGGT

AGTCGGGATACATCATCAAGAGGCGCTCCTTGTGCATCTTTGC

>YM7

CGCCCGTTGGGGAAGTCGGGAGCCAAAGAATCGGGGACGGAGTGGAGGTGCGGGACATTC

ATCTGGAGCAGCGACCGGAAAAAAGATCAGACCTAGTTATTCCCGTGATGCGCAGTCGAG

ACAGTGGCTGGGCATCATGGATGGTGTTGGCAGTCTGCAGTGGCGAAGAGCAACATGTGG

GCGAATCAATGGTCCTCACGGCGGGGGCCAAAAGACACTCTAGGTATCGGGCTGGAATAG

ATGGTCCATGAAGATGATATTTACTGGCTTGCAGATTATAATTACAGAAAAAGATTCGTA

TTCGCTTGACATGCGAATACATGGGTTGTGAGTACATTTTTGCACCAATATGTTCATCAA

ATCGAAGATCTCTGCTTCGCGTTGCCATCGAAGCAGATGGTCCAGGATGATTCTTGGTTC

CTCAGATCCGGTGATCTCCTAGCGTTCGTACGTCTAATTATCGCCGCACAAAGGTCTGTT

GGTTTCTCGCGCAGGTAACTCTTGCCACAGCATGCGGGAAGTGAATCGACGAACAGATGC

GTGGTCGCCTCCTGGGTCACGCTGACTGGATAGAGATCGAAGACAGCCTCCAAAGGCGCC

ATGTTCTGAGGCTCCTCTGGTCGCTGATGCTGCCAGTCTAGAACACAAGGACGACCTAAA

GTCGTTTGGCAGCGCCGATATCTATTCCGTTCCATGACTGATACTACAGCGACTTGTCGC

AGTCGGTAGGTATAACTCATACTCCTCCGTCTGGAGCCGACAAGTGGGCGCGCTGATGGT

TTTGTCGCTCTCGGGTAGGAGGCGGCCTCTACCTCATCGACGCTGCTAAGCTGGTCTTCT

TGCTTTGGCCACTAGGCTTTCGATGGCGCTTCTCAGACGGCTTGCGAGGGGTGTAACGGT

AGTCGGGATACATCATCAAGAGGCGCTCCTTGTGCATCTTTGC

>ZH6

GAGCCAAAGAATCGGGGACGGAGTGGAGGTGCGGGACGTTCATCTGGAGCAGCGACTGGA

AAAAAGATCAGTACCTAGTTATTCCCGTGATGCGCAGTCGAGACAGTGGCTGGGCATCAT

GGATGGTGTTGGCAGTCTGCAGTGGCGAAGAGCAACATGTGGGCGAATCAATGGTCCTCA

CGGCGGGGGCCAAAAGACACTCTAGGTATCGGGCTGGAATAGATGGTCCATGAAGATGAT

ATTTACTGGCTTGCAGATTATAATTACAGAAAAAGATTCGTATTCGCTTGACATGCGAAT

ACATGGGTTGTGAGTACATTTTTGCACCAATATGTTCATCAAATCGAAGATCTCTGCTTC

GCGTTGCCATCGAAGCAGATGGTCCAGGATGATTCTTGGTTCCTCAGATCCGGTGATCTC

CTAGCGTTCGTACGTCTAATTATCGCCGCACAAAGGTCTGTTGGTTTCTCGCGCAGGTAA

CTCTTGCCACAGCATGCGGGAAGTGAATCGACGAACAGATGCGTGGTCGCCTCCTGGGTC

ACGCTGACTGGATAGAGATCGAAGACAGCCTCCAAAGGCGCCATGTTCTGAGGCTCCTCT

GGTCGCTGATGCTGCCAGTCTAGAACACAAGGACGACCTAAAGTCGTTTGGCAGCGCCGA

TATCTATTCCGTTCCATGACTGATACTACAGCGACTTGTCGCAGTCGGTAGGTATAACTC

ATACTCCTCCGTCTGGAGCCGACAAGTGGGCGCGCTGATGGTTTTGTCGCTCTCGGGTAG

GAGGCGGCCTCTACCTCATCGACGCTGCTAAGCTGGTCTTCTTGCTTTGGCCACTAGGCT

TTCGATGGCGCTTCTCAGACGGCTTGCGAGGGGTGTAACGGTAGTCGGGATACATCATCA

AGA

>BM6

GTGTGCCGCCCGTTGGGGAAGTCGGGGGTCAAAGAATCGGGGACCGAGTGGAGATGCGGG

ACCTTCATCTGGAGCAGCGACTGGAAAAAAGATCAGACCTAGTATTCCCGTGATGCGCGG

TCGTGACAGTGGCGAAGAGCATCACAAGGGCGAATCATTATTTTTATGCTAGGAAAAGAA

ATGACACTCTAGGTATCGGGCTGGAATAGATGGTCTATGAAGATGATATTTACTGGCTTG

CGGATTATAATTACAGAAAAAGGTTCGTGTTCGCTTTGAACATGCGAATACATGGGTTGT

GAGTACATTCTTCTACCAATATGTTCATCAAATCGAAGATCTCTGGTTCGCGTAGCCATC

GAAGCAGATGGGCCAGGATGATTCTTGGTTCGTCAGATCCGGTGATCTCCTAGCGTTCGT

ACGTCTAAGTATCGCCGCACAAAGGTCTGTTGGTTTCCGGCGCAGGGTAACTCTTGCCGC

AGCATATAGGAAGGGAATCGATGACCAGATGCATGGTTGCCTGCTTGGCCACGCTGACTG

GATAGAGATCGAAGACAGCTGCACAAAGGCGCCATATTCTTAGGCTTCCCTGGTCGCTGA

TGCTGCCAGTCAAGGGCACAAAGACAACCTAAGGTGGTCTGGCAGCGCCGATATCGGTTC

CAATTCGTTGCTGATGCTACAGCGACTTGTCGCAGTCGGTAGGTGTAACTCATACTCCTC

CGTCTTGAGCTGACAAGTCGGCGCGCTGATGGTGTCGTCGCTCTCGGGTAGGAGACGACC

TCTACCTCATCGACGCTGCTAAGCTGGCCTTCTTGCTTTGGCCACTGGGCTTTCGATGAC

GCTTCTCAGACGGCTTGCGTGGGGTGTAACGGTAGTCGGGGTACATCATCAGGAGGCGCT

CCTTGTGCATCTTTGCAAGTCG

>F8

GTGTGCCGCCCGTTGGGGAAGTCGGGGGTCAAAGAATCGGGGACCGAGTGGAGATGCGGG

ACCTTCATCTGGAGCAGCGACTGGAAAAAAGATCAGACCTAGTATTCCCGTGATGCGCGG

TCGTGACAGTGGCGAAGAGCATCACAAGGGCGAATCATTATTTTTATGCTAGGAAAAGAA

ATGACACTCTAGGTATCGGGCTGGAATAGATGGTCTATGAAGATGATATTTACTGGCTTG

CGGATTATAATTACAGAAAAAGGTTCGTGTTCGCTTTGAACATGCGAATACATGGGTTGT

GAGTACATTCTTCTACCAATATGTTCATCAAATCGAAGATCTCTGGTTCGCGTAGCCATC

GAAGCAGATGGGCCAGGATGATTCTTGGTTCGTCAGATCCGGTGATCTCCTAGCGTTCGT

ACGTCTAAGTATCGCCGCACAAAGGTCTGTTGGTTTCCGGCGCAGGGTAACTCTTGCCGC

AGCATATAGGAAGGGAATCGATGACCAGATGCATGGTTGCCTGCTTGGCCACGCTGACTG

GATAGAGATCGAAGACAGCTGCACAAAGGCGCCATATTCTTAGGCTTCCCTGGTCGCTGA

TGCTGCCAGTCAAGGGCACAAAGACAACCTAAGGTGGTCTGGCAGCGCCGATATCGGTTC

CAATTCGTTGCTGATGCTACAGCGACTTGTCGCAGTCGGTAGGTGTAACTCATACTCCTC

CGTCTTGAGCTGACAAGTCGGCGCGCTGATGGTGTCGTCGCTCTCGGGTAGGAGACGACC

TCTACCTCATCGACGCTGCTAAGCTGGCCTTCTTGCTTTGGCCACTGGGCTTTCGATGAC

GCTTCTCAGACGGCTTGCGTGGGGTGTAACGGTAGTCGGGGTACATCATCAGGAGGCGCT

CCTTGTGCATCTTTGCAAGTCG

>GX3

GTGTGCCGCCCGTTGGGGAAGTCGGGGGTCAAAGAATCGGGGACCGAGTGGAGATGCGGG

ACCTTCATCTGGAGCAGCGACTGGAAAAAAGATCAGACCTAGTATTCCCGTGATGCGCGG

TCGTGACAGTGGCGAAGAGCATCACAAGGGCGAATCATTATTTTTATGCTAGGAAAAGAA

ATGACACTCTAGGTATCGGGCTGGAATAGATGGTCTATGAAGATGATATTTACTGGCTTG

CGGATTATAATTACAGAAAAAGGTTCGTGTTCGCTTTGAACATGCGAATACATGGGTTGT

GAGTACATTCTTCTACCAATATGTTCATCAAATCGAAGATCTCTGGTTCGCGTAGCCATC

GAAGCAGATGGGCCAGGATGATTCTTGGTTCGTCAGATCCGGTGATCTCCTAGCGTTCGT

ACGTCTAAGTATCGCCGCACAAAGGTCTGTTGGTTTCCGGCGCAGGGTAACTCTTGCCGC

AGCATATAGGAAGGGAATCGATGACCAGATGCATGGTTGCCTGCTTGGCCACGCTGACTG

GATAGAGATCGAAGACAGCTGCACAAAGGCGCCATATTCTTAGGCTTCCCTGGTCGCTGA

TGCTGCCAGTCAAGGGCACAAAGACAACCTAAGGTGGTCTGGCAGCGCCGATATCGGTTC

CAATTCGTTGCTGATGCTACAGCGACTTGTCGCAGTCGGTAGGTGTAACTCATACTCCTC

CGTCTTGAGCTGACAAGTCGGCGCGCTGATGGTGTCGTCGCTCTCGGGTAGGAGACGACC

TCTACCTCATCGACGCTGCTAAGCTGGCCTTCTTGCTTTGGCCACTGGGCTTTCGATGAC

GCTTCTCAGACGGCTTGCGTGGGGTGTAACGGTAGTCGGGGTACATCATCAGGAGGCGCT

CCTTGTGCATCTTTGCAAGTCG

>JS1

GTGTGCCGCCCGTTGGGGAAGTCGGGGGTCAAAGAATCGGGGACCGAGTGGAGATGCGGG

ACCTTCATCTGGAGCAGCGACTGGAAAAAAGATCAGACCTAGTATTCCCGTGATGCGCGG

TCGTGACAGTGGCGAAGAGCATCACAAGGGCGAATCATTATTTTTATGCTAGGAAAAGAA

ATGACACTCTAGGTATCGGGCTGGAATAGATGGTCTATGAAGATGATATTTACTGGCTTG

CGGATTATAATTACAGAAAAAGGTTCGTGTTCGCTTTGAACATGCGAATACATGGGTTGT

GAGTACATTCTTCTACCAATATGTTCATCAAATCGAAGATCTCTGGTTCGCGTAGCCATC

GAAGCAGATGGGCCAGGATGATTCTTGGTTCGTCAGATCCGGTGATCTCCTAGCGTTCGT

ACGTCTAAGTATCGCCGCACAAAGGTCTGTTGGTTTCCGGCGCAGGGTAACTCTTGCCGC

AGCATATAGGAAGGGAATCGATGACCAGATGCATGGTTGCCTGCTTGGCCACGCTGACTG

GATAGAGATCGAAGACAGCTGCACAAAGGCGCCATATTCTTAGGCTTCCCTGGTCGCTGA

TGCTGCCAGTCAAGGGCACAAAGACAACCTAAGGTGGTCTGGCAGCGCCGATATCGGTTC

CAATTCGTTGCTGATGCTACAGCGACTTGTCGCAGTCGGTAGGTGTAACTCATACTCCTC

CGTCTTGAGCTGACAAGTCGGCGCGCTGATGGTGTCGTCGCTCTCGGGTAGGAGACGACC

TCTACCTCATCGACGCTGCTAAGCTGGCCTTCTTGCTTTGGCCACTGGGCTTTCGATGAC

GCTTCTCAGACGGCTTGCGTGGGGTGTAACGGTAGTCGGGGTACATCATCAGGAGGCGCT

CCTTGTGCATCTTTGCAAGTCG

>JS5

GTGTGCCGCCCGTTGGGGAAGTCGGGGGTCAAAGAATCGGGGACCGAGTGGAGATGCGGG

ACCTTCATCTGGAGCAGCGACTGGAAAAAAGATCAGACCTAGTATTCCCGTGATGCGCGG

TCGTGACAGTGGCGAAGAGCATCACAAGGGCGAATCATTATTTTTATGCTAGGAAAAGAA

ATGACACTCTAGGTATCGGGCTGGAATAGATGGTCTATGAAGATGATATTTACTGGCTTG

CGGATTATAATTACAGAAAAAGGTTCGTGTTCGCTTTGAACATGCGAATACATGGGTTGT

GAGTACATTCTTCTACCAATATGTTCATCAAATCGAAGATCTCTGGTTCGCGTAGCCATC

GAAGCAGATGGGCCAGGATGATTCTTGGTTCGTCAGATCCGGTGATCTCCTAGCGTTCGT

ACGTCTAAGTATCGCCGCACAAAGGTCTGTTGGTTTCCGGCGCAGGGTAACTCTTGCCGC

AGCATATAGGAAGGGAATCGATGACCAGATGCATGGTTGCCTGCTTGGCCACGCTGACTG

GATAGAGATCGAAGACAGCTGCACAAAGGCGCCATATTCTTAGGCTTCCCTGGTCGCTGA

TGCTGCCAGTCAAGGGCACAAAGACAACCTAAGGTGGTCTGGCAGCGCCGATATCGGTTC

CAATTCGTTGCTGATGCTACAGCGACTTGTCGCAGTCGGTAGGTGTAACTCATACTCCTC

CGTCTTGAGCTGACAAGTCGGCGCGCTGATGGTGTCGTCGCTCTCGGGTAGGAGACGACC

TCTACCTCATCGACGCTGCTAAGCTGGCCTTCTTGCTTTGGCCACTGGGCTTTCGATGAC

GCTTCTCAGACGGCTTGCGTGGGGTGTAACGGTAGTCGGGGTACATCATCAGGAGGCGCT

CCTTGTGCATCTTTGCAAGTCG

>LC2

GTGTGCCGCCCGTTGGGGAAGTCGGGGGTCAAAGAATCGGGGACCGAGTGGAGATGCGGG

ACCTTCATCTGGAGCAGCGACTGGAAAAAAGATCAGACCTAGTATTCCCGTGATGCGCGG

TCGTGACAGTGGCGAAGAGCATCACAAGGGCGAATCATTATTTTTATGCTAGGAAAAGAA

ATGACACTCTAGGTATCGGGCTGGAATAGATGGTCTATGAAGATGATATTTACTGGCTTG

CGGATTATAATTACAGAAAAAGGTTCGTGTTCGCTTTGAACATGCGAATACATGGGTTGT

GAGTACATTCTTCTACCAATATGTTCATCAAATCGAAGATCTCTGGTTCGCGTAGCCATC

GAAGCAGATGGGCCAGGATGATTCTTGGTTCGTCAGATCCGGTGATCTCCTAGCGTTCGT

ACGTCTAAGTATCGCCGCACAAAGGTCTGTTGGTTTCCGGCGCAGGGTAACTCTTGCCGC

AGCATATAGGAAGGGAATCGATGACCAGATGCATGGTTGCCTGCTTGGCCACGCTGACTG

GATAGAGATCGAAGACAGCTGCACAAAGGCGCCATATTCTTAGGCTTCCCTGGTCGCTGA

TGCTGCCAGTCAAGGGCACAAAGACAACCTAAGGTGGTCTGGCAGCGCCGATATCGGTTC

CAATTCGTTGCTGATGCTACAGCGACTTGTCGCAGTCGGTAGGTGTAACTCATACTCCTC

CGTCTTGAGCTGACAAGTCGGCGCGCTGATGGTGTCGTCGCTCTCGGGTAGGAGACGACC

TCTACCTCATCGACGCTGCTAAGCTGGCCTTCTTGCTTTGGCCACTGGGCTTTCGATGAC

GCTTCTCAGACGGCTTGCGTGGGGTGTAACGGTAGTCGGGGTACATCATCAGGAGGCGCT

CCTTGTGCATCTTTGCAAGTCG

>LC6

GTGTGCCGCCCGTTGGGGAAGTCGGGGGTCAAAGAATCGGGGACCGAGTGGAGATGCGGG

ACCTTCATCTGGAGCAGCGACTGGAAAAAAGATCAGACCTAGTATTCCCGTGATGCGCGG

TCGTGACAGTGGCGAAGAGCATCACAAGGGCGAATCATTATTTTTATGCTAGGAAAAGAA

ATGACACTCTAGGTATCGGGCTGGAATAGATGGTCTATGAAGATGATATTTACTGGCTTG

CGGATTATAATTACAGAAAAAGGTTCGTGTTCGCTTTGAACATGCGAATACATGGGTTGT

GAGTACATTCTTCTACCAATATGTTCATCAAATCGAAGATCTCTGGTTCGCGTAGCCATC

GAAGCAGATGGGCCAGGATGATTCTTGGTTCGTCAGATCCGGTGATCTCCTAGCGTTCGT

ACGTCTAAGTATCGCCGCACAAAGGTCTGTTGGTTTCCGGCGCAGGGTAACTCTTGCCGC

AGCATATAGGAAGGGAATCGATGACCAGATGCATGGTTGCCTGCTTGGCCACGCTGACTG

GATAGAGATCGAAGACAGCTGCACAAAGGCGCCATATTCTTAGGCTTCCCTGGTCGCTGA

TGCTGCCAGTCAAGGGCACAAAGACAACCTAAGGTGGTCTGGCAGCGCCGATATCGGTTC

CAATTCGTTGCTGATGCTACAGCGACTTGTCGCAGTCGGTAGGTGTAACTCATACTCCTC

CGTCTTGAGCTGACAAGTCGGCGCGCTGATGGTGTCGTCGCTCTCGGGTAGGAGACGACC

TCTACCTCATCGACGCTGCTAAGCTGGCCTTCTTGCTTTGGCCACTGGGCTTTCGATGAC

GCTTCTCAGACGGCTTGCGTGGGGTGTAACGGTAGTCGGGGTACATCATCAGGAGGCGCT

CCTTGTGCATCTTTGCAAGTCG

>YM4

GTGTGCCGCCCGTTGGGGAAGTCGGGGGTCAAAGAATCGGGGACCGAGTGGAGATGCGGG

ACCTTCATCTGGAGCAGCGACTGGAAAAAAGATCAGACCTAGTATTCCCGTGATGCGCGG

TCGTGACAGTGGCGAAGAGCATCACAAGGGCGAATCATTATTTTTATGCTAGGAAAAGAA

ATGACACTCTAGGTATCGGGCTGGAATAGATGGTCTATGAAGATGATATTTACTGGCTTG

CGGATTATAATTACAGAAAAAGGTTCGTGTTCGCTTTGAACATGCGAATACATGGGTTGT

GAGTACATTCTTCTACCAATATGTTCATCAAATCGAAGATCTCTGGTTCGCGTAGCCATC

GAAGCAGATGGGCCAGGATGATTCTTGGTTCGTCAGATCCGGTGATCTCCTAGCGTTCGT

ACGTCTAAGTATCGCCGCACAAAGGTCTGTTGGTTTCCGGCGCAGGGTAACTCTTGCCGC

AGCATATAGGAAGGGAATCGATGACCAGATGCATGGTTGCCTGCTTGGCCACGCTGACTG

GATAGAGATCGAAGACAGCTGCACAAAGGCGCCATATTCTTAGGCTTCCCTGGTCGCTGA

TGCTGCCAGTCAAGGGCACAAAGACAACCTAAGGTGGTCTGGCAGCGCCGATATCGGTTC

CAATTCGTTGCTGATGCTACAGCGACTTGTCGCAGTCGGTAGGTGTAACTCATACTCCTC

CGTCTTGAGCTGACAAGTCGGCGCGCTGATGGTGTCGTCGCTCTCGGGTAGGAGACGACC

TCTACCTCATCGACGCTGCTAAGCTGGCCTTCTTGCTTTGGCCACTGGGCTTTCGATGAC

GCTTCTCAGACGGCTTGCGTGGGGTGTAACGGTAGTCGGGGTACATCATCAGGAGGCGCT

CCTTGTGCATCTTTGCAAGTCG

>YM5

GTGTGCCGCCCGTTGGGGAAGTCGGGGGTCAAAGAATCGGGGACCGAGTGGAGATGCGGG

ACCTTCATCTGGAGCAGCGACTGGAAAAAAGATCAGACCTAGTATTCCCGTGATGCGCGG

TCGTGACAGTGGCGAAGAGCATCACAAGGGCGAATCATTATTTTTATGCTAGGAAAAGAA

ATGACACTCTAGGTATCGGGCTGGAATAGATGGTCTATGAAGATGATATTTACTGGCTTG

CGGATTATAATTACAGAAAAAGGTTCGTGTTCGCTTTGAACATGCGAATACATGGGTTGT

GAGTACATTCTTCTACCAATATGTTCATCAAATCGAAGATCTCTGGTTCGCGTAGCCATC

GAAGCAGATGGGCCAGGATGATTCTTGGTTCGTCAGATCCGGTGATCTCCTAGCGTTCGT

ACGTCTAAGTATCGCCGCACAAAGGTCTGTTGGTTTCCGGCGCAGGGTAACTCTTGCCGC

AGCATATAGGAAGGGAATCGATGACCAGATGCATGGTTGCCTGCTTGGCCACGCTGACTG

GATAGAGATCGAAGACAGCTGCACAAAGGCGCCATATTCTTAGGCTTCCCTGGTCGCTGA

TGCTGCCAGTCAAGGGCACAAAGACAACCTAAGGTGGTCTGGCAGCGCCGATATCGGTTC

CAATTCGTTGCTGATGCTACAGCGACTTGTCGCAGTCGGTAGGTGTAACTCATACTCCTC

CGTCTTGAGCTGACAAGTCGGCGCGCTGATGGTGTCGTCGCTCTCGGGTAGGAGACGACC

TCTACCTCATCGACGCTGCTAAGCTGGCCTTCTTGCTTTGGCCACTGGGCTTTCGATGAC

GCTTCTCAGACGGCTTGCGTGGGGTGTAACGGTAGTCGGGGTACATCATCAGGAGGCGCT

CCTTGTGCATCTTTGCAAGTCG

>ZH3

GTGTGCCGCCCGTTGGGGAAGTCGGGGGTCAAAGAATCGGGGACCGAGTGGAGATGCGGG

ACCTTCATCTGGAGCAGCGACTGGAAAAAAGATCAGACCTAGTATTCCCGTGATGCGCGG

TCGTGACAGTGGCGAAGAGCATCACAAGGGCGAATCATTATTTTTATGCTAGGAAAAGAA

ATGACACTCTAGGTATCGGGCTGGAATAGATGGTCTATGAAGATGATATTTACTGGCTTG

CGGATTATAATTACAGAAAAAGGTTCGTGTTCGCTTTGAACATGCGAATACATGGGTTGT

GAGTACATTCTTCTACCAATATGTTCATCAAATCGAAGATCTCTGGTTCGCGTAGCCATC

GAAGCAGATGGGCCAGGATGATTCTTGGTTCGTCAGATCCGGTGATCTCCTAGCGTTCGT

ACGTCTAAGTATCGCCGCACAAAGGTCTGTTGGTTTCCGGCGCAGGGTAACTCTTGCCGC

AGCATATAGGAAGGGAATCGATGACCAGATGCATGGTTGCCTGCTTGGCCACGCTGACTG

GATAGAGATCGAAGACAGCTGCACAAAGGCGCCATATTCTTAGGCTTCCCTGGTCGCTGA

TGCTGCCAGTCAAGGGCACAAAGACAACCTAAGGTGGTCTGGCAGCGCCGATATCGGTTC

CAATTCGTTGCTGATGCTACAGCGACTTGTCGCAGTCGGTAGGTGTAACTCATACTCCTC

CGTCTTGAGCTGACAAGTCGGCGCGCTGATGGTGTCGTCGCTCTCGGGTAGGAGACGACC

TCTACCTCATCGACGCTGCTAAGCTGGCCTTCTTGCTTTGGCCACTGGGCTTTCGATGAC

GCTTCTCAGACGGCTTGCGTGGGGTGTAACGGTAGTCGGGGTACATCATCAGGAGGCGCT

CCTTGTGCATCTTTGCAAGTCG
