# Supplementary material for: Etiology of Cyclocarya paliurus Anthracnose in Jiangsu Province, China
Source: Front Plant Sci. 2021 Jan 18;11:613499. doi: 10.3389/fpls.2020.613499 (PMC7847979; doi:10.3389/fpls.2020.613499)
Supplement: Supplementary file 2 [file Table_2.DOCX]

>HC3

TGTCACACCCGCCGGGCGTTGGCTCGGTTCGCCGCCCGCAAAATCGCCCGCACTGCTGCA

GCCGGAGAATTCTTTACACGAACAAAAGGATACGCCTCTTCCAGCGTTGGCCCGCCACGC

GTTGCAGCTGAGCCGGTTAATGCCCTCTCACGGCCTGCGGCGGCGGGCGCAACAAAGCTG

GGGAAGCGGCCCCGTGTTTTGAGGAATCATTGCCTCGGGGTCTCTCCGAGTCTGCCCCGG

ACTGAGATTTAGGCGGGCTGCTGCAGCAGGTTGCGGCGACGGCAAGCACTGGGGCTTGGC

GGGGGTCAAACCACCGCCTGCCCCGCGGATGCTGTGTTGGGTGTTGGGTTGGCGGTCGTT

GTACGTACCTGCCCAGCTCTGCTCGGCTGGGCTTCTGGTTATTGCCAGTAGCCGGGGCTG

CTGGCTGACAGGATTCGCACACGACTCGGTTCTGCAAGGACCCCCGCCCCACCTGGCTGG

TGCAGCGACTCCGAGGTCTCGCCTTGCCTCCAGCTGCAGGGTTCAACGCGGCATCTCGGG

TATTGCAGGTCTTGCAAACAAAAAAGTCTAGAGGCGTCTTTGTCCGCTCGCCGTCGGGGC

GCATCCTTTTTGCCGGTTGGCCAGCCGCCGGCGCGACGTGATAGTGGTCACGACCCCACT

GGCGATGGGCCCGAGACCCAAAAGCCCCGAATGAGCGACTGGAGCTCCCCGCTTGAGCAT

CGTCAGTTCGACGACAAGGCGCATCGCATGATGTCAGCCTTGCCGGCCAAAGTTGGCCAC

GCGCCGAGCATCTACCATGCCCAGACTTGCATCATAACGCCACATTCACAACATCGATAC

TGACAATGTGTGCCTACAGACACTCAA

>JS2

TGTCACACCCGCCGGGCGTTGGCTCGGTTCGCCGCCCGCAAAATCGCCCGCACTGCTGCA

GCCGGAGAATTCTTTACACGAACAAAAGGATACGCCTCTTCCAGCGTTGGCCCGCCACGC

GTTGCAGCTGAGCCGGTTAATGCCCTCTCACGGCCTGCGGCGGCGGGCGCAACAAAGCTG

GGGAAGCGGCCCCGTGTTTTGAGGAATCATTGCCTCGGGGTCTCTCCGAGTCTGCCCCGG

ACTGAGATTTAGGCGGGCTGCTGCAGCAGGTTGCGGCGACGGCAAGCACTGGGGCTTGGC

GGGGGTCAAACCACCGCCTGCCCCGCGGATGCTGTGTTGGGTGTTGGGTTGGCGGTCGTT

GTACGTACCTGCCCAGCTCTGCTCGGCTGGGCTTCTGGTTATTGCCAGTAGCCGGGGCTG

CTGGCTGACAGGATTCGCACACGACTCGGTTCTGCAAGGACCCCCGCCCCACCTGGCTGG

TGCAGCGACTCCGAGGTCTCGCCTTGCCTCCAGCTGCAGGGTTCAACGCGGCATCTCGGG

TATTGCAGGTCTTGCAAACAAAAAAGTCTAGAGGCGTCTTTGTCCGCTCGCCGTCGGGGC

GCATCCTTTTTGCCGGTTGGCCAGCCGCCGGCGCGACGTGATAGTGGTCACGACCCCACT

GGCGATGGGCCCGAGACCCAAAAGCCCCGAATGAGCGACTGGAGCTCCCCGCTTGAGCAT

CGTCAGTTCGACGACAAGGCGCATCGCATGATGTCAGCCTTGCCGGCCAAAGTTGGCCAC

GCGCCGAGCATCTACCATGCCCAGACTTGCATCATAACGCCACATTCACAACATCGATAC

TGACAATGTGTGCCTACAGACACTCAA

>JS7

TGTCACACCCGCCGGGCGTTGGCTCGGTTCGCCGCCCGCAAAATCGCCCGCACTGCTGCA

GCCGGAGAATTCTTTACACGAACAAAAGGATACGCCTCTTCCAGCGTTGGCCCGCCACGC

GTTGCAGCTGAGCCGGTTAATGCCCTCTCACGGCCTGCGGCGGCGGGCGCAACAAAGCTG

GGGAAGCGGCCCCGTGTTTTGAGGAATCATTGCCTCGGGGTCTCTCCGAGTCTGCCCCGG

ACTGAGATTTAGGCGGGCTGCTGCAGCAGGTTGCGGCGACGGCAAGCACTGGGGCTTGGC

GGGGGTCAAACCACCGCCTGCCCCGCGGATGCTGTGTTGGGTGTTGGGTTGGCGGTCGTT

GTACGTACCTGCCCAGCTCTGCTCGGCTGGGCTTCTGGTTATTGCCAGTAGCCGGGGCTG

CTGGCTGACAGGATTCGCACACGACTCGGTTCTGCAAGGACCCCCGCCCCACCTGGCTGG

TGCAGCGACTCCGAGGTCTCGCCTTGCCTCCAGCTGCAGGGTTCAACGCGGCATCTCGGG

TATTGCAGGTCTTGCAAACAAAAAAGTCTAGAGGCGTCTTTGTCCGCTCGCCGTCGGGGC

GCATCCTTTTTGCCGGTTGGCCAGCCGCCGGCGCGACGTGATAGTGGTCACGACCCCACT

GGCGATGGGCCCGAGACCCAAAAGCCCCGAATGAGCGACTGGAGCTCCCCGCTTGAGCAT

CGTCAGTTCGACGACAAGGCGCATCGCATGATGTCAGCCTTGCCGGCCAAAGTTGGCCAC

GCGCCGAGCATCTACCATGCCCAGACTTGCATCATAACGCCACATTCACAACATCGATAC

TGACAATGTGTGCCTACAGACACTCAA

>SC7

TGTCACACCCGCCGGGCGTTGGCTCGGTTCGCCGCCCGCAAAATCGCCCGCACTGCTGCA

GCCGGAGAATTCTTTACACGAACAAAAGGATACGCCTCTTCCAGCGTTGGCCCGCCACGC

GTTGCAGCTGAGCCGGTTAATGCCCTCTCACGGCCTGCGGCGGCGGGCGCAACAAAGCTG

GGGAAGCGGCCCCGTGTTTTGAGGAATCATTGCCTCGGGGTCTCTCCGAGTCTGCCCCGG

ACTGAGATTTAGGCGGGCTGCTGCAGCAGGTTGCGGCGACGGCAAGCACTGGGGCTTGGC

GGGGGTCAAACCACCGCCTGCCCCGCGGATGCTGTGTTGGGTGTTGGGTTGGCGGTCGTT

GTACGTACCTGCCCAGCTCTGCTCGGCTGGGCTTCTGGTTATTGCCAGTAGCCGGGGCTG

CTGGCTGACAGGATTCGCACACGACTCGGTTCTGCAAGGACCCCCGCCCCACCTGGCTGG

TGCAGCGACTCCGAGGTCTCGCCTTGCCTCCAGCTGCAGGGTTCAACGCGGCATCTCGGG

TATTGCAGGTCTTGCAAACAAAAAAGTCTAGAGGCGTCTTTGTCCGCTCGCCGTCGGGGC

GCATCCTTTTTGCCGGTTGGCCAGCCGCCGGCGCGACGTGATAGTGGTCACGACCCCACT

GGCGATGGGCCCGAGACCCAAAAGCCCCGAATGAGCGACTGGAGCTCCCCGCTTGAGCAT

CGTCAGTTCGACGACAAGGCGCATCGCATGATGTCAGCCTTGCCGGCCAAAGTTGGCCAC

GCGCCGAGCATCTACCATGCCCAGACTTGCATCATAACGCCACATTCACAACATCGATAC

TGACAATGTGTGCCTACAGACACTCAA

>YM8

TGTCACACCCGCCGGGCGTTGGCTCGGTTCGCCGCCCGCAAAATCGCCCGCACTGCTGCA

GCCGGAGAATTCTTTACACGAACAAAAGGATACGCCTCTTCCAGCGTTGGCCCGCCACGC

GTTGCAGCTGAGCCGGTTAATGCCCTCTCACGGCCTGCGGCGGCGGGCGCAACAAAGCTG

GGGAAGCGGCCCCGTGTTTTGAGGAATCATTGCCTCGGGGTCTCTCCGAGTCTGCCCCGG

ACTGAGATTTAGGCGGGCTGCTGCAGCAGGTTGCGGCGACGGCAAGCACTGGGGCTTGGC

GGGGGTCAAACCACCGCCTGCCCCGCGGATGCTGTGTTGGGTGTTGGGTTGGCGGTCGTT

GTACGTACCTGCCCAGCTCTGCTCGGCTGGGCTTCTGGTTATTGCCAGTAGCCGGGGCTG

CTGGCTGACAGGATTCGCACACGACTCGGTTCTGCAAGGACCCCCGCCCCACCTGGCTGG

TGCAGCGACTCCGAGGTCTCGCCTTGCCTCCAGCTGCAGGGTTCAACGCGGCATCTCGGG

TATTGCAGGTCTTGCAAACAAAAAAGTCTAGAGGCGTCTTTGTCCGCTCGCCGTCGGGGC

GCATCCTTTTTGCCGGTTGGCCAGCCGCCGGCGCGACGTGATAGTGGTCACGACCCCACT

GGCGATGGGCCCGAGACCCAAAAGCCCCGAATGAGCGACTGGAGCTCCCCGCTTGAGCAT

CGTCAGTTCGACGACAAGGCGCATCGCATGATGTCAGCCTTGCCGGCCAAAGTTGGCCAC

GCGCCGAGCATCTACCATGCCCAGACTTGCATCATAACGCCACATTCACAACATCGATAC

TGACAATGTGTGCCTACAGACACTCAA

>ZH2

TGTCACACCCGCCGGGCGTTGGCTCGGTTCGCCGCCCGCAAAATCGCCCGCACTGCTGCA

GCCGGAGAATTCTTTACACGAACAAAAGGATACGCCTCTTCCAGCGTTGGCCCGCCACGC

GTTGCAGCTGAGCCGGTTAATGCCCTCTCACGGCCTGCGGCGGCGGGCGCAACAAAGCTG

GGGAAGCGGCCCCGTGTTTTGAGGAATCATTGCCTCGGGGTCTCTCCGAGTCTGCCCCGG

ACTGAGATTTAGGCGGGCTGCTGCAGCAGGTTGCGGCGACGGCAAGCACTGGGGCTTGGC

GGGGGTCAAACCACCGCCTGCCCCGCGGATGCTGTGTTGGGTGTTGGGTTGGCGGTCGTT

GTACGTACCTGCCCAGCTCTGCTCGGCTGGGCTTCTGGTTATTGCCAGTAGCCGGGGCTG

CTGGCTGACAGGATTCGCACACGACTCGGTTCTGCAAGGACCCCCGCCCCACCTGGCTGG

TGCAGCGACTCCGAGGTCTCGCCTTGCCTCCAGCTGCAGGGTTCAACGCGGCATCTCGGG

TATTGCAGGTCTTGCAAACAAAAAAGTCTAGAGGCGTCTTTGTCCGCTCGCCGTCGGGGC

GCATCCTTTTTGCCGGTTGGCCAGCCGCCGGCGCGACGTGATAGTGGTCACGACCCCACT

GGCGATGGGCCCGAGACCCAAAAGCCCCGAATGAGCGACTGGAGCTCCCCGCTTGAGCAT

CGTCAGTTCGACGACAAGGCGCATCGCATGATGTCAGCCTTGCCGGCCAAAGTTGGCCAC

GCGCCGAGCATCTACCATGCCCAGACTTGCATCATAACGCCACATTCACAACATCGATAC

TGACAATGTGTGCCTACAGACACTCAA

>BM5

TCACATCCGCCGGGCGTTGGCTCGGTTCGCCGCCCGCACAATCGCCCGCACTGCTGCAGCCGGAGAATCC

TTTACACGAGCAAAAGGATACGCCTCTCCCAGCGTTGGCCCGCCAGGCATTGCAGCTGAGCCGGTTAATG

CCTCTCACGGCCTGCGGCGGCGGGCGCAACAAAGCTGGGGAAGCGGCCCCGTGTTTTGAGGAATCATTGC

CTCGGGGTCTCTCCGAGTCTGCCCCGGACTGAGATTTAGGCGGGCTGCTGCAGCAGGTTGCGGCGACGGC

AAGCACTGGGGCTTGGCGGGGGTCAAACCACCGCCTGCCCCGCGGATGCTGTGTTGGGTGTTGGGTTGGC

GGTCGTTGTACGTACCTGCTCAGCTCTGCTCGGCTGGGCTTCTGGTTATTGCCAGTAGCCGGGGCTGCTG

GCTGACAGGATTCGCACACGACTCGGTTCTGCAAGGAACCCCACCCCACCTGGCTGGTGCAGCGACTCCG

AGGCCTCGCCTTGCCTCCAGCTGCAGGGTTCAACGCGGCATCTCGGGTATTGCAGGTCTTGCAAACAACA

AGTCTAGAGGCGTCTTTGTCCGCTCGCCGTCGGGGCGCATCCTTTTTGCCGGTTGGCCAGCCGCCGGCGC

GACGTGATAGCGGTCACGACCCCACTGGCGATGGGCCTGAGACCCAAAAGCCCCGAATGAGCGACTGGAG

CTCCCCGCTTGAGCATCGTCAGTTCGACGACAAGGCGCATCGCATGATGTCAGCCTTGCCGGCCAAAGTT

GGCCACGCGCCGAGCATCTACCATGCCCAGACTTGCATCATAACGCCCATTCATGACATCGATACTGACA

ATGTGTGCCTACAGACACT

>BX1

TCACATCCGCCGGGCGTTGGCTCGGTTCGCCGCCCGCACAATCGCCCGCACTGCTGCAGCCGGAGAATCC

TTTACACGAGCAAAAGGATACGCCTCTCCCAGCGTTGGCCCGCCAGGCATTGCAGCTGAGCCGGTTAATG

CCTCTCACGGCCTGCGGCGGCGGGCGCAACAAAGCTGGGGAAGCGGCCCCGTGTTTTGAGGAATCATTGC

CTCGGGGTCTCTCCGAGTCTGCCCCGGACTGAGATTTAGGCGGGCTGCTGCAGCAGGTTGCGGCGACGGC

AAGCACTGGGGCTTGGCGGGGGTCAAACCACCGCCTGCCCCGCGGATGCTGTGTTGGGTGTTGGGTTGGC

GGTCGTTGTACGTACCTGCTCAGCTCTGCTCGGCTGGGCTTCTGGTTATTGCCAGTAGCCGGGGCTGCTG

GCTGACAGGATTCGCACACGACTCGGTTCTGCAAGGAACCCCACCCCACCTGGCTGGTGCAGCGACTCCG

AGGCCTCGCCTTGCCTCCAGCTGCAGGGTTCAACGCGGCATCTCGGGTATTGCAGGTCTTGCAAACAACA

AGTCTAGAGGCGTCTTTGTCCGCTCGCCGTCGGGGCGCATCCTTTTTGCCGGTTGGCCAGCCGCCGGCGC

GACGTGATAGCGGTCACGACCCCACTGGCGATGGGCCTGAGACCCAAAAGCCCCGAATGAGCGACTGGAG

CTCCCCGCTTGAGCATCGTCAGTTCGACGACAAGGCGCATCGCATGATGTCAGCCTTGCCGGCCAAAGTT

GGCCACGCGCCGAGCATCTACCATGCCCAGACTTGCATCATAACGCCCATTCATGACATCGATACTGACA

ATGTGTGCCTACAGACACT

>F5

TCACATCCGCCGGGCGTTGGCTCGGTTCGCCGCCCGCACAATCGCCCGCACTGCTGCAGCCGGAGAATCC

TTTACACGAGCAAAAGGATACGCCTCTCCCAGCGTTGGCCCGCCAGGCATTGCAGCTGAGCCGGTTAATG

CCTCTCACGGCCTGCGGCGGCGGGCGCAACAAAGCTGGGGAAGCGGCCCCGTGTTTTGAGGAATCATTGC

CTCGGGGTCTCTCCGAGTCTGCCCCGGACTGAGATTTAGGCGGGCTGCTGCAGCAGGTTGCGGCGACGGC

AAGCACTGGGGCTTGGCGGGGGTCAAACCACCGCCTGCCCCGCGGATGCTGTGTTGGGTGTTGGGTTGGC

GGTCGTTGTACGTACCTGCTCAGCTCTGCTCGGCTGGGCTTCTGGTTATTGCCAGTAGCCGGGGCTGCTG

GCTGACAGGATTCGCACACGACTCGGTTCTGCAAGGAACCCCACCCCACCTGGCTGGTGCAGCGACTCCG

AGGCCTCGCCTTGCCTCCAGCTGCAGGGTTCAACGCGGCATCTCGGGTATTGCAGGTCTTGCAAACAACA

AGTCTAGAGGCGTCTTTGTCCGCTCGCCGTCGGGGCGCATCCTTTTTGCCGGTTGGCCAGCCGCCGGCGC

GACGTGATAGCGGTCACGACCCCACTGGCGATGGGCCTGAGACCCAAAAGCCCCGAATGAGCGACTGGAG

CTCCCCGCTTGAGCATCGTCAGTTCGACGACAAGGCGCATCGCATGATGTCAGCCTTGCCGGCCAAAGTT

GGCCACGCGCCGAGCATCTACCATGCCCAGACTTGCATCATAACGCCCATTCATGACATCGATACTGACA

ATGTGTGCCTACAGACACT

>GX1

GCAGCCGGAGAATCCTTTACACGAGCAAAAGGATACGCCTCTCCCAGCGTTGGCCCGCCA

GGCATTGCAGCTGAGCCGGTTAATGCCTCTCACGGCCTGCGGCGGCGGGCGCAACAAAGC

TGGGGAAGCGGCCCCGTGTTTTGAGGAATCATTGCCTCGGGGTCTCTCCGAGTCTGCCCC

GGACTGAGATTTAGGCGGGCTGCTGCAGCAGGTTGCGGCGACGGCAAGCACTGGGGCTTG

GCGGGGGTCAAACCACCGCCTGCCCCGCGGATGCTGTGTTGGGTGTTGGGTTGGCGGTCG

TTGTACGTACCTGCTCAGCTCTGCTCGGCTGGGCTTCTGGTTATTGCCAGTAGCCGGGGC

TGCTGGCTGACAGGATTCGCACACGACTCGGTTCTGCAAGGAACCCCACCCCACCTGGCT

GGTGCAGCGACTCCGAGGCCTCGCCTTGCCTCCAGCTGCAGGGTTCAACGCGGCATCTCG

GGTATTGCAGGTCTTGCAAACAACAAGTCTAGAGGCGTCTTTGTCCGCTCGCCGTCGGGG

CGCATCCTTTTTGCCGGTTGGCCAGCCGCCGGCGCGACGTGATAGCGGTCACGACCCCAC

TGGCGATGGGCCTGAGACCCAAAAGCCCCGAATGAGCGACTGGAGCTCCCCGCTTGAGCA

TCGTCAGTTCGACGACAAGGCGCATCGCATGATGTCAGCCTTGCCGGCCAAAGTTGGCCA

CGCGCCGAGCATCTACCATGCCCAGACTTGCATCATAACGCCCATTCATGACATCGATAC

TGACAATGTGTGCCTACAGACACTCAA

>GT7

GCAGCCGGAGAATCCTTTACACGAGCAAAAGGATACGCCTCTCCCAGCGTTGGCCCGCCA

GGCATTGCAGCTGAGCCGGTTAATGCCTCTCACGGCCTGCGGCGGCGGGCGCAACAAAGC

TGGGGAAGCGGCCCCGTGTTTTGAGGAATCATTGCCTCGGGGTCTCTCCGAGTCTGCCCC

GGACTGAGATTTAGGCGGGCTGCTGCAGCAGGTTGCGGCGACGGCAAGCACTGGGGCTTG

GCGGGGGTCAAACCACCGCCTGCCCCGCGGATGCTGTGTTGGGTGTTGGGTTGGCGGTCG

TTGTACGTACCTGCTCAGCTCTGCTCGGCTGGGCTTCTGGTTATTGCCAGTAGCCGGGGC

TGCTGGCTGACAGGATTCGCACACGACTCGGTTCTGCAAGGAACCCCACCCCACCTGGCT

GGTGCAGCGACTCCGAGGCCTCGCCTTGCCTCCAGCTGCAGGGTTCAACGCGGCATCTCG

GGTATTGCAGGTCTTGCAAACAACAAGTCTAGAGGCGTCTTTGTCCGCTCGCCGTCGGGG

CGCATCCTTTTTGCCGGTTGGCCAGCCGCCGGCGCGACGTGATAGCGGTCACGACCCCAC

TGGCGATGGGCCTGAGACCCAAAAGCCCCGAATGAGCGACTGGAGCTCCCCGCTTGAGCA

TCGTCAGTTCGACGACAAGGCGCATCGCATGATGTCAGCCTTGCCGGCCAAAGTTGGCCA

CGCGCCGAGCATCTACCATGCCCAGACTTGCATCATAACGCCCATTCATGACATCGATAC

TGACAATGTGTGCCTACAGACACTCAA

>HC2

GCAGCCGGAGAATCCTTTACACGAGCAAAAGGATACGCCTCTCCCAGCGTTGGCCCGCCA

GGCATTGCAGCTGAGCCGGTTAATGCCTCTCACGGCCTGCGGCGGCGGGCGCAACAAAGC

TGGGGAAGCGGCCCCGTGTTTTGAGGAATCATTGCCTCGGGGTCTCTCCGAGTCTGCCCC

GGACTGAGATTTAGGCGGGCTGCTGCAGCAGGTTGCGGCGACGGCAAGCACTGGGGCTTG

GCGGGGGTCAAACCACCGCCTGCCCCGCGGATGCTGTGTTGGGTGTTGGGTTGGCGGTCG

TTGTACGTACCTGCTCAGCTCTGCTCGGCTGGGCTTCTGGTTATTGCCAGTAGCCGGGGC

TGCTGGCTGACAGGATTCGCACACGACTCGGTTCTGCAAGGAACCCCACCCCACCTGGCT

GGTGCAGCGACTCCGAGGCCTCGCCTTGCCTCCAGCTGCAGGGTTCAACGCGGCATCTCG

GGTATTGCAGGTCTTGCAAACAACAAGTCTAGAGGCGTCTTTGTCCGCTCGCCGTCGGGG

CGCATCCTTTTTGCCGGTTGGCCAGCCGCCGGCGCGACGTGATAGCGGTCACGACCCCAC

TGGCGATGGGCCTGAGACCCAAAAGCCCCGAATGAGCGACTGGAGCTCCCCGCTTGAGCA

TCGTCAGTTCGACGACAAGGCGCATCGCATGATGTCAGCCTTGCCGGCCAAAGTTGGCCA

CGCGCCGAGCATCTACCATGCCCAGACTTGCATCATAACGCCCATTCATGACATCGATAC

TGACAATGTGTGCCTACAGACACTCAA

>HC6

GCAGCCGGAGAATCCTTTACACGAGCAAAAGGATACGCCTCTCCCAGCGTTGGCCCGCCA

GGCATTGCAGCTGAGCCGGTTAATGCCTCTCACGGCCTGCGGCGGCGGGCGCAACAAAGC

TGGGGAAGCGGCCCCGTGTTTTGAGGAATCATTGCCTCGGGGTCTCTCCGAGTCTGCCCC

GGACTGAGATTTAGGCGGGCTGCTGCAGCAGGTTGCGGCGACGGCAAGCACTGGGGCTTG

GCGGGGGTCAAACCACCGCCTGCCCCGCGGATGCTGTGTTGGGTGTTGGGTTGGCGGTCG

TTGTACGTACCTGCTCAGCTCTGCTCGGCTGGGCTTCTGGTTATTGCCAGTAGCCGGGGC

TGCTGGCTGACAGGATTCGCACACGACTCGGTTCTGCAAGGAACCCCACCCCACCTGGCT

GGTGCAGCGACTCCGAGGCCTCGCCTTGCCTCCAGCTGCAGGGTTCAACGCGGCATCTCG

GGTATTGCAGGTCTTGCAAACAACAAGTCTAGAGGCGTCTTTGTCCGCTCGCCGTCGGGG

CGCATCCTTTTTGCCGGTTGGCCAGCCGCCGGCGCGACGTGATAGCGGTCACGACCCCAC

TGGCGATGGGCCTGAGACCCAAAAGCCCCGAATGAGCGACTGGAGCTCCCCGCTTGAGCA

TCGTCAGTTCGACGACAAGGCGCATCGCATGATGTCAGCCTTGCCGGCCAAAGTTGGCCA

CGCGCCGAGCATCTACCATGCCCAGACTTGCATCATAACGCCCATTCATGACATCGATAC

TGACAATGTGTGCCTACAGACACTCAA

>JS3

GCAGCCGGAGAATCCTTTACACGAGCAAAAGGATACGCCTCTCCCAGCGTTGGCCCGCCA

GGCATTGCAGCTGAGCCGGTTAATGCCTCTCACGGCCTGCGGCGGCGGGCGCAACAAAGC

TGGGGAAGCGGCCCCGTGTTTTGAGGAATCATTGCCTCGGGGTCTCTCCGAGTCTGCCCC

GGACTGAGATTTAGGCGGGCTGCTGCAGCAGGTTGCGGCGACGGCAAGCACTGGGGCTTG

GCGGGGGTCAAACCACCGCCTGCCCCGCGGATGCTGTGTTGGGTGTTGGGTTGGCGGTCG

TTGTACGTACCTGCTCAGCTCTGCTCGGCTGGGCTTCTGGTTATTGCCAGTAGCCGGGGC

TGCTGGCTGACAGGATTCGCACACGACTCGGTTCTGCAAGGAACCCCACCCCACCTGGCT

GGTGCAGCGACTCCGAGGCCTCGCCTTGCCTCCAGCTGCAGGGTTCAACGCGGCATCTCG

GGTATTGCAGGTCTTGCAAACAACAAGTCTAGAGGCGTCTTTGTCCGCTCGCCGTCGGGG

CGCATCCTTTTTGCCGGTTGGCCAGCCGCCGGCGCGACGTGATAGCGGTCACGACCCCAC

TGGCGATGGGCCTGAGACCCAAAAGCCCCGAATGAGCGACTGGAGCTCCCCGCTTGAGCA

TCGTCAGTTCGACGACAAGGCGCATCGCATGATGTCAGCCTTGCCGGCCAAAGTTGGCCA

CGCGCCGAGCATCTACCATGCCCAGACTTGCATCATAACGCCCATTCATGACATCGATAC

TGACAATGTGTGCCTACAGACACTCAA

>JS9

GCAGCCGGAGAATCCTTTACACGAGCAAAAGGATACGCCTCTCCCAGCGTTGGCCCGCCA

GGCATTGCAGCTGAGCCGGTTAATGCCTCTCACGGCCTGCGGCGGCGGGCGCAACAAAGC

TGGGGAAGCGGCCCCGTGTTTTGAGGAATCATTGCCTCGGGGTCTCTCCGAGTCTGCCCC

GGACTGAGATTTAGGCGGGCTGCTGCAGCAGGTTGCGGCGACGGCAAGCACTGGGGCTTG

GCGGGGGTCAAACCACCGCCTGCCCCGCGGATGCTGTGTTGGGTGTTGGGTTGGCGGTCG

TTGTACGTACCTGCTCAGCTCTGCTCGGCTGGGCTTCTGGTTATTGCCAGTAGCCGGGGC

TGCTGGCTGACAGGATTCGCACACGACTCGGTTCTGCAAGGAACCCCACCCCACCTGGCT

GGTGCAGCGACTCCGAGGCCTCGCCTTGCCTCCAGCTGCAGGGTTCAACGCGGCATCTCG

GGTATTGCAGGTCTTGCAAACAACAAGTCTAGAGGCGTCTTTGTCCGCTCGCCGTCGGGG

CGCATCCTTTTTGCCGGTTGGCCAGCCGCCGGCGCGACGTGATAGCGGTCACGACCCCAC

TGGCGATGGGCCTGAGACCCAAAAGCCCCGAATGAGCGACTGGAGCTCCCCGCTTGAGCA

TCGTCAGTTCGACGACAAGGCGCATCGCATGATGTCAGCCTTGCCGGCCAAAGTTGGCCA

CGCGCCGAGCATCTACCATGCCCAGACTTGCATCATAACGCCCATTCATGACATCGATAC

TGACAATGTGTGCCTACAGACACTCAA

>LC7

GCAGCCGGAGAATCCTTTACACGAGCAAAAGGATACGCCTCTCCCAGCGTTGGCCCGCCA

GGCATTGCAGCTGAGCCGGTTAATGCCTCTCACGGCCTGCGGCGGCGGGCGCAACAAAGC

TGGGGAAGCGGCCCCGTGTTTTGAGGAATCATTGCCTCGGGGTCTCTCCGAGTCTGCCCC

GGACTGAGATTTAGGCGGGCTGCTGCAGCAGGTTGCGGCGACGGCAAGCACTGGGGCTTG

GCGGGGGTCAAACCACCGCCTGCCCCGCGGATGCTGTGTTGGGTGTTGGGTTGGCGGTCG

TTGTACGTACCTGCTCAGCTCTGCTCGGCTGGGCTTCTGGTTATTGCCAGTAGCCGGGGC

TGCTGGCTGACAGGATTCGCACACGACTCGGTTCTGCAAGGAACCCCACCCCACCTGGCT

GGTGCAGCGACTCCGAGGCCTCGCCTTGCCTCCAGCTGCAGGGTTCAACGCGGCATCTCG

GGTATTGCAGGTCTTGCAAACAACAAGTCTAGAGGCGTCTTTGTCCGCTCGCCGTCGGGG

CGCATCCTTTTTGCCGGTTGGCCAGCCGCCGGCGCGACGTGATAGCGGTCACGACCCCAC

TGGCGATGGGCCTGAGACCCAAAAGCCCCGAATGAGCGACTGGAGCTCCCCGCTTGAGCA

TCGTCAGTTCGACGACAAGGCGCATCGCATGATGTCAGCCTTGCCGGCCAAAGTTGGCCA

CGCGCCGAGCATCTACCATGCCCAGACTTGCATCATAACGCCCATTCATGACATCGATAC

TGACAATGTGTGCCTACAGACACTCAA

>LG2

GCAGCCGGAGAATCCTTTACACGAGCAAAAGGATACGCCTCTCCCAGCGTTGGCCCGCCA

GGCATTGCAGCTGAGCCGGTTAATGCCTCTCACGGCCTGCGGCGGCGGGCGCAACAAAGC

TGGGGAAGCGGCCCCGTGTTTTGAGGAATCATTGCCTCGGGGTCTCTCCGAGTCTGCCCC

GGACTGAGATTTAGGCGGGCTGCTGCAGCAGGTTGCGGCGACGGCAAGCACTGGGGCTTG

GCGGGGGTCAAACCACCGCCTGCCCCGCGGATGCTGTGTTGGGTGTTGGGTTGGCGGTCG

TTGTACGTACCTGCTCAGCTCTGCTCGGCTGGGCTTCTGGTTATTGCCAGTAGCCGGGGC

TGCTGGCTGACAGGATTCGCACACGACTCGGTTCTGCAAGGAACCCCACCCCACCTGGCT

GGTGCAGCGACTCCGAGGCCTCGCCTTGCCTCCAGCTGCAGGGTTCAACGCGGCATCTCG

GGTATTGCAGGTCTTGCAAACAACAAGTCTAGAGGCGTCTTTGTCCGCTCGCCGTCGGGG

CGCATCCTTTTTGCCGGTTGGCCAGCCGCCGGCGCGACGTGATAGCGGTCACGACCCCAC

TGGCGATGGGCCTGAGACCCAAAAGCCCCGAATGAGCGACTGGAGCTCCCCGCTTGAGCA

TCGTCAGTTCGACGACAAGGCGCATCGCATGATGTCAGCCTTGCCGGCCAAAGTTGGCCA

CGCGCCGAGCATCTACCATGCCCAGACTTGCATCATAACGCCCATTCATGACATCGATAC

TGACAATGTGTGCCTACAGACACTCAA

>LG4

GCAGCCGGAGAATCCTTTACACGAGCAAAAGGATACGCCTCTCCCAGCGTTGGCCCGCCA

GGCATTGCAGCTGAGCCGGTTAATGCCTCTCACGGCCTGCGGCGGCGGGCGCAACAAAGC

TGGGGAAGCGGCCCCGTGTTTTGAGGAATCATTGCCTCGGGGTCTCTCCGAGTCTGCCCC

GGACTGAGATTTAGGCGGGCTGCTGCAGCAGGTTGCGGCGACGGCAAGCACTGGGGCTTG

GCGGGGGTCAAACCACCGCCTGCCCCGCGGATGCTGTGTTGGGTGTTGGGTTGGCGGTCG

TTGTACGTACCTGCTCAGCTCTGCTCGGCTGGGCTTCTGGTTATTGCCAGTAGCCGGGGC

TGCTGGCTGACAGGATTCGCACACGACTCGGTTCTGCAAGGAACCCCACCCCACCTGGCT

GGTGCAGCGACTCCGAGGCCTCGCCTTGCCTCCAGCTGCAGGGTTCAACGCGGCATCTCG

GGTATTGCAGGTCTTGCAAACAACAAGTCTAGAGGCGTCTTTGTCCGCTCGCCGTCGGGG

CGCATCCTTTTTGCCGGTTGGCCAGCCGCCGGCGCGACGTGATAGCGGTCACGACCCCAC

TGGCGATGGGCCTGAGACCCAAAAGCCCCGAATGAGCGACTGGAGCTCCCCGCTTGAGCA

TCGTCAGTTCGACGACAAGGCGCATCGCATGATGTCAGCCTTGCCGGCCAAAGTTGGCCA

CGCGCCGAGCATCTACCATGCCCAGACTTGCATCATAACGCCCATTCATGACATCGATAC

TGACAATGTGTGCCTACAGACACTCAA

>LV2

GCAGCCGGAGAATCCTTTACACGAGCAAAAGGATACGCCTCTCCCAGCGTTGGCCCGCCA

GGCATTGCAGCTGAGCCGGTTAATGCCTCTCACGGCCTGCGGCGGCGGGCGCAACAAAGC

TGGGGAAGCGGCCCCGTGTTTTGAGGAATCATTGCCTCGGGGTCTCTCCGAGTCTGCCCC

GGACTGAGATTTAGGCGGGCTGCTGCAGCAGGTTGCGGCGACGGCAAGCACTGGGGCTTG

GCGGGGGTCAAACCACCGCCTGCCCCGCGGATGCTGTGTTGGGTGTTGGGTTGGCGGTCG

TTGTACGTACCTGCTCAGCTCTGCTCGGCTGGGCTTCTGGTTATTGCCAGTAGCCGGGGC

TGCTGGCTGACAGGATTCGCACACGACTCGGTTCTGCAAGGAACCCCACCCCACCTGGCT

GGTGCAGCGACTCCGAGGCCTCGCCTTGCCTCCAGCTGCAGGGTTCAACGCGGCATCTCG

GGTATTGCAGGTCTTGCAAACAACAAGTCTAGAGGCGTCTTTGTCCGCTCGCCGTCGGGG

CGCATCCTTTTTGCCGGTTGGCCAGCCGCCGGCGCGACGTGATAGCGGTCACGACCCCAC

TGGCGATGGGCCTGAGACCCAAAAGCCCCGAATGAGCGACTGGAGCTCCCCGCTTGAGCA

TCGTCAGTTCGACGACAAGGCGCATCGCATGATGTCAGCCTTGCCGGCCAAAGTTGGCCA

CGCGCCGAGCATCTACCATGCCCAGACTTGCATCATAACGCCCATTCATGACATCGATAC

TGACAATGTGTGCCTACAGACACTCAA

>NC25

TCACATCCGCCGGGCGTTGGCTCGGTTCGCCGCCCGCACAATCGCCCGCACTGCTGCAGCCGGAGAATCC

TTTACACGAGCAAAAGGATACGCCTCTCCCAGCGTTGGCCCGCCAGGCATTGCAGCTGAGCCGGTTAATG

CCTCTCACGGCCTGCGGCGGCGGGCGCAACAAAGCTGGGGAAGCGGCCCCGTGTTTTGAGGAATCATTGC

CTCGGGGTCTCTCCGAGTCTGCCCCGGACTGAGATTTAGGCGGGCTGCTGCAGCAGGTTGCGGCGACGGC

AAGCACTGGGGCTTGGCGGGGGTCAAACCACCGCCTGCCCCGCGGATGCTGTGTTGGGTGTTGGGTTGGC

GGTCGTTGTACGTACCTGCTCAGCTCTGCTCGGCTGGGCTTCTGGTTATTGCCAGTAGCCGGGGCTGCTG

GCTGACAGGATTCGCACACGACTCGGTTCTGCAAGGAACCCCACCCCACCTGGCTGGTGCAGCGACTCCG

AGGCCTCGCCTTGCCTCCAGCTGCAGGGTTCAACGCGGCATCTCGGGTATTGCAGGTCTTGCAAACAACA

AGTCTAGAGGCGTCTTTGTCCGCTCGCCGTCGGGGCGCATCCTTTTTGCCGGTTGGCCAGCCGCCGGCGC

GACGTGATAGCGGTCACGACCCCACTGGCGATGGGCCTGAGACCCAAAAGCCCCGAATGAGCGACTGGAG

CTCCCCGCTTGAGCATCGTCAGTTCGACGACAAGGCGCATCGCATGATGTCAGCCTTGCCGGCCAAAGTT

GGCCACGCGCCGAGCATCTACCATGCCCAGACTTGCATCATAACGCCCATTCATGACATCGATACTGACA

ATGTGTGCCTACAGACACT

>NC26

TCACATCCGCCGGGCGTTGGCTCGGTTCGCCGCCCGCACAATCGCCCGCACTGCTGCAGCCGGAGAATCC

TTTACACGAGCAAAAGGATACGCCTCTCCCAGCGTTGGCCCGCCAGGCATTGCAGCTGAGCCGGTTAATG

CCTCTCACGGCCTGCGGCGGCGGGCGCAACAAAGCTGGGGAAGCGGCCCCGTGTTTTGAGGAATCATTGC

CTCGGGGTCTCTCCGAGTCTGCCCCGGACTGAGATTTAGGCGGGCTGCTGCAGCAGGTTGCGGCGACGGC

AAGCACTGGGGCTTGGCGGGGGTCAAACCACCGCCTGCCCCGCGGATGCTGTGTTGGGTGTTGGGTTGGC

GGTCGTTGTACGTACCTGCTCAGCTCTGCTCGGCTGGGCTTCTGGTTATTGCCAGTAGCCGGGGCTGCTG

GCTGACAGGATTCGCACACGACTCGGTTCTGCAAGGAACCCCACCCCACCTGGCTGGTGCAGCGACTCCG

AGGCCTCGCCTTGCCTCCAGCTGCAGGGTTCAACGCGGCATCTCGGGTATTGCAGGTCTTGCAAACAACA

AGTCTAGAGGCGTCTTTGTCCGCTCGCCGTCGGGGCGCATCCTTTTTGCCGGTTGGCCAGCCGCCGGCGC

GACGTGATAGCGGTCACGACCCCACTGGCGATGGGCCTGAGACCCAAAAGCCCCGAATGAGCGACTGGAG

CTCCCCGCTTGAGCATCGTCAGTTCGACGACAAGGCGCATCGCATGATGTCAGCCTTGCCGGCCAAAGTT

GGCCACGCGCCGAGCATCTACCATGCCCAGACTTGCATCATAACGCCCATTCATGACATCGATACTGACA

ATGTGTGCCTACAGACACT

>PL2

GCAGCCGGAGAATCCTTTACACGAGCAAAAGGATACGCCTCTCCCAGCGTTGGCCCGCCA

GGCATTGCAGCTGAGCCGGTTAATGCCTCTCACGGCCTGCGGCGGCGGGCGCAACAAAGC

TGGGGAAGCGGCCCCGTGTTTTGAGGAATCATTGCCTCGGGGTCTCTCCGAGTCTGCCCC

GGACTGAGATTTAGGCGGGCTGCTGCAGCAGGTTGCGGCGACGGCAAGCACTGGGGCTTG

GCGGGGGTCAAACCACCGCCTGCCCCGCGGATGCTGTGTTGGGTGTTGGGTTGGCGGTCG

TTGTACGTACCTGCTCAGCTCTGCTCGGCTGGGCTTCTGGTTATTGCCAGTAGCCGGGGC

TGCTGGCTGACAGGATTCGCACACGACTCGGTTCTGCAAGGAACCCCACCCCACCTGGCT

GGTGCAGCGACTCCGAGGCCTCGCCTTGCCTCCAGCTGCAGGGTTCAACGCGGCATCTCG

GGTATTGCAGGTCTTGCAAACAACAAGTCTAGAGGCGTCTTTGTCCGCTCGCCGTCGGGG

CGCATCCTTTTTGCCGGTTGGCCAGCCGCCGGCGCGACGTGATAGCGGTCACGACCCCAC

TGGCGATGGGCCTGAGACCCAAAAGCCCCGAATGAGCGACTGGAGCTCCCCGCTTGAGCA

TCGTCAGTTCGACGACAAGGCGCATCGCATGATGTCAGCCTTGCCGGCCAAAGTTGGCCA

CGCGCCGAGCATCTACCATGCCCAGACTTGCATCATAACGCCCATTCATGACATCGATAC

TGACAATGTGTGCCTACAGACACTCAA

>PX3

GCAGCCGGAGAATCCTTTACACGAGCAAAAGGATACGCCTCTCCCAGCGTTGGCCCGCCA

GGCATTGCAGCTGAGCCGGTTAATGCCTCTCACGGCCTGCGGCGGCGGGCGCAACAAAGC

TGGGGAAGCGGCCCCGTGTTTTGAGGAATCATTGCCTCGGGGTCTCTCCGAGTCTGCCCC

GGACTGAGATTTAGGCGGGCTGCTGCAGCAGGTTGCGGCGACGGCAAGCACTGGGGCTTG

GCGGGGGTCAAACCACCGCCTGCCCCGCGGATGCTGTGTTGGGTGTTGGGTTGGCGGTCG

TTGTACGTACCTGCTCAGCTCTGCTCGGCTGGGCTTCTGGTTATTGCCAGTAGCCGGGGC

TGCTGGCTGACAGGATTCGCACACGACTCGGTTCTGCAAGGAACCCCACCCCACCTGGCT

GGTGCAGCGACTCCGAGGCCTCGCCTTGCCTCCAGCTGCAGGGTTCAACGCGGCATCTCG

GGTATTGCAGGTCTTGCAAACAACAAGTCTAGAGGCGTCTTTGTCCGCTCGCCGTCGGGG

CGCATCCTTTTTGCCGGTTGGCCAGCCGCCGGCGCGACGTGATAGCGGTCACGACCCCAC

TGGCGATGGGCCTGAGACCCAAAAGCCCCGAATGAGCGACTGGAGCTCCCCGCTTGAGCA

TCGTCAGTTCGACGACAAGGCGCATCGCATGATGTCAGCCTTGCCGGCCAAAGTTGGCCA

CGCGCCGAGCATCTACCATGCCCAGACTTGCATCATAACGCCCATTCATGACATCGATAC

TGACAATGTGTGCCTACAGACACTCAA

>SC6

GCAGCCGGAGAATCCTTTACACGAGCAAAAGGATACGCCTCTCCCAGCGTTGGCCCGCCA

GGCATTGCAGCTGAGCCGGTTAATGCCTCTCACGGCCTGCGGCGGCGGGCGCAACAAAGC

TGGGGAAGCGGCCCCGTGTTTTGAGGAATCATTGCCTCGGGGTCTCTCCGAGTCTGCCCC

GGACTGAGATTTAGGCGGGCTGCTGCAGCAGGTTGCGGCGACGGCAAGCACTGGGGCTTG

GCGGGGGTCAAACCACCGCCTGCCCCGCGGATGCTGTGTTGGGTGTTGGGTTGGCGGTCG

TTGTACGTACCTGCTCAGCTCTGCTCGGCTGGGCTTCTGGTTATTGCCAGTAGCCGGGGC

TGCTGGCTGACAGGATTCGCACACGACTCGGTTCTGCAAGGAACCCCACCCCACCTGGCT

GGTGCAGCGACTCCGAGGCCTCGCCTTGCCTCCAGCTGCAGGGTTCAACGCGGCATCTCG

GGTATTGCAGGTCTTGCAAACAACAAGTCTAGAGGCGTCTTTGTCCGCTCGCCGTCGGGG

CGCATCCTTTTTGCCGGTTGGCCAGCCGCCGGCGCGACGTGATAGCGGTCACGACCCCAC

TGGCGATGGGCCTGAGACCCAAAAGCCCCGAATGAGCGACTGGAGCTCCCCGCTTGAGCA

TCGTCAGTTCGACGACAAGGCGCATCGCATGATGTCAGCCTTGCCGGCCAAAGTTGGCCA

CGCGCCGAGCATCTACCATGCCCAGACTTGCATCATAACGCCCATTCATGACATCGATAC

TGACAATGTGTGCCTACAGACACTCAA

>SC9

GCAGCCGGAGAATCCTTTACACGAGCAAAAGGATACGCCTCTCCCAGCGTTGGCCCGCCA

GGCATTGCAGCTGAGCCGGTTAATGCCTCTCACGGCCTGCGGCGGCGGGCGCAACAAAGC

TGGGGAAGCGGCCCCGTGTTTTGAGGAATCATTGCCTCGGGGTCTCTCCGAGTCTGCCCC

GGACTGAGATTTAGGCGGGCTGCTGCAGCAGGTTGCGGCGACGGCAAGCACTGGGGCTTG

GCGGGGGTCAAACCACCGCCTGCCCCGCGGATGCTGTGTTGGGTGTTGGGTTGGCGGTCG

TTGTACGTACCTGCTCAGCTCTGCTCGGCTGGGCTTCTGGTTATTGCCAGTAGCCGGGGC

TGCTGGCTGACAGGATTCGCACACGACTCGGTTCTGCAAGGAACCCCACCCCACCTGGCT

GGTGCAGCGACTCCGAGGCCTCGCCTTGCCTCCAGCTGCAGGGTTCAACGCGGCATCTCG

GGTATTGCAGGTCTTGCAAACAACAAGTCTAGAGGCGTCTTTGTCCGCTCGCCGTCGGGG

CGCATCCTTTTTGCCGGTTGGCCAGCCGCCGGCGCGACGTGATAGCGGTCACGACCCCAC

TGGCGATGGGCCTGAGACCCAAAAGCCCCGAATGAGCGACTGGAGCTCCCCGCTTGAGCA

TCGTCAGTTCGACGACAAGGCGCATCGCATGATGTCAGCCTTGCCGGCCAAAGTTGGCCA

CGCGCCGAGCATCTACCATGCCCAGACTTGCATCATAACGCCCATTCATGACATCGATAC

TGACAATGTGTGCCTACAGACACTCAA

>T5

GCAGCCGGAGAATCCTTTACACGAGCAAAAGGATACGCCTCTCCCAGCGTTGGCCCGCCA

GGCATTGCAGCTGAGCCGGTTAATGCCTCTCACGGCCTGCGGCGGCGGGCGCAACAAAGC

TGGGGAAGCGGCCCCGTGTTTTGAGGAATCATTGCCTCGGGGTCTCTCCGAGTCTGCCCC

GGACTGAGATTTAGGCGGGCTGCTGCAGCAGGTTGCGGCGACGGCAAGCACTGGGGCTTG

GCGGGGGTCAAACCACCGCCTGCCCCGCGGATGCTGTGTTGGGTGTTGGGTTGGCGGTCG

TTGTACGTACCTGCTCAGCTCTGCTCGGCTGGGCTTCTGGTTATTGCCAGTAGCCGGGGC

TGCTGGCTGACAGGATTCGCACACGACTCGGTTCTGCAAGGAACCCCACCCCACCTGGCT

GGTGCAGCGACTCCGAGGCCTCGCCTTGCCTCCAGCTGCAGGGTTCAACGCGGCATCTCG

GGTATTGCAGGTCTTGCAAACAACAAGTCTAGAGGCGTCTTTGTCCGCTCGCCGTCGGGG

CGCATCCTTTTTGCCGGTTGGCCAGCCGCCGGCGCGACGTGATAGCGGTCACGACCCCAC

TGGCGATGGGCCTGAGACCCAAAAGCCCCGAATGAGCGACTGGAGCTCCCCGCTTGAGCA

TCGTCAGTTCGACGACAAGGCGCATCGCATGATGTCAGCCTTGCCGGCCAAAGTTGGCCA

CGCGCCGAGCATCTACCATGCCCAGACTTGCATCATAACGCCCATTCATGACATCGATAC

TGACAATGTGTGCCTACAGACACTCAA

>T9

GCAGCCGGAGAATCCTTTACACGAGCAAAAGGATACGCCTCTCCCAGCGTTGGCCCGCCA

GGCATTGCAGCTGAGCCGGTTAATGCCTCTCACGGCCTGCGGCGGCGGGCGCAACAAAGC

TGGGGAAGCGGCCCCGTGTTTTGAGGAATCATTGCCTCGGGGTCTCTCCGAGTCTGCCCC

GGACTGAGATTTAGGCGGGCTGCTGCAGCAGGTTGCGGCGACGGCAAGCACTGGGGCTTG

GCGGGGGTCAAACCACCGCCTGCCCCGCGGATGCTGTGTTGGGTGTTGGGTTGGCGGTCG

TTGTACGTACCTGCTCAGCTCTGCTCGGCTGGGCTTCTGGTTATTGCCAGTAGCCGGGGC

TGCTGGCTGACAGGATTCGCACACGACTCGGTTCTGCAAGGAACCCCACCCCACCTGGCT

GGTGCAGCGACTCCGAGGCCTCGCCTTGCCTCCAGCTGCAGGGTTCAACGCGGCATCTCG

GGTATTGCAGGTCTTGCAAACAACAAGTCTAGAGGCGTCTTTGTCCGCTCGCCGTCGGGG

CGCATCCTTTTTGCCGGTTGGCCAGCCGCCGGCGCGACGTGATAGCGGTCACGACCCCAC

TGGCGATGGGCCTGAGACCCAAAAGCCCCGAATGAGCGACTGGAGCTCCCCGCTTGAGCA

TCGTCAGTTCGACGACAAGGCGCATCGCATGATGTCAGCCTTGCCGGCCAAAGTTGGCCA

CGCGCCGAGCATCTACCATGCCCAGACTTGCATCATAACGCCCATTCATGACATCGATAC

TGACAATGTGTGCCTACAGACACTCAA

>H3

TCACATCCGCCGGGCGTTGGCTCGGTTCGCCGCCCGCACAATCGCCCGCACTGCTGCAGCCGGAGAATCC

TTTACACGAGCAAAAGGATACGCCTCTCCCAGCGTTGGCCCGCCAGGCATTGCAGCTGAGCCGGTTAATG

CCTCTCACGGCCTGCGGCGGCGGGCGCAACAAAGCTGGGGAAGCGGCCCCGTGTTTTGAGGAATCATTGC

CTCGGGGTCTCTCCGAGTCTGCCCCGGACTGAGATTTAGGCGGGCTGCTGCAGCAGGTTGCGGCGACGGC

AAGCACTGGGGCTTGGCGGGGGTCAAACCACCGCCTGCCCCGCGGATGCTGTGTTGGGTGTTGGGTTGGC

GGTCGTTGTACGTACCTGCTCAGCTCTGCTCGGCTGGGCTTCTGGTTATTGCCAGTAGCCGGGGCTGCTG

GCTGACAGGATTCGCACACGACTCGGTTCTGCAAGGAACCCCACCCCACCTGGCTGGTGCAGCGACTCCG

AGGCCTCGCCTTGCCTCCAGCTGCAGGGTTCAACGCGGCATCTCGGGTATTGCAGGTCTTGCAAACAACA

AGTCTAGAGGCGTCTTTGTCCGCTCGCCGTCGGGGCGCATCCTTTTTGCCGGTTGGCCAGCCGCCGGCGC

GACGTGATAGCGGTCACGACCCCACTGGCGATGGGCCTGAGACCCAAAAGCCCCGAATGAGCGACTGGAG

CTCCCCGCTTGAGCATCGTCAGTTCGACGACAAGGCGCATCGCATGATGTCAGCCTTGCCGGCCAAAGTT

GGCCACGCGCCGAGCATCTACCATGCCCAGACTTGCATCATAACGCCCATTCATGACATCGATACTGACA

ATGTGTGCCTACAGACACT

>H4

TCACATCCGCCGGGCGTTGGCTCGGTTCGCCGCCCGCACAATCGCCCGCACTGCTGCAGCCGGAGAATCC

TTTACACGAGCAAAAGGATACGCCTCTCCCAGCGTTGGCCCGCCAGGCATTGCAGCTGAGCCGGTTAATG

CCTCTCACGGCCTGCGGCGGCGGGCGCAACAAAGCTGGGGAAGCGGCCCCGTGTTTTGAGGAATCATTGC

CTCGGGGTCTCTCCGAGTCTGCCCCGGACTGAGATTTAGGCGGGCTGCTGCAGCAGGTTGCGGCGACGGC

AAGCACTGGGGCTTGGCGGGGGTCAAACCACCGCCTGCCCCGCGGATGCTGTGTTGGGTGTTGGGTTGGC

GGTCGTTGTACGTACCTGCTCAGCTCTGCTCGGCTGGGCTTCTGGTTATTGCCAGTAGCCGGGGCTGCTG

GCTGACAGGATTCGCACACGACTCGGTTCTGCAAGGAACCCCACCCCACCTGGCTGGTGCAGCGACTCCG

AGGCCTCGCCTTGCCTCCAGCTGCAGGGTTCAACGCGGCATCTCGGGTATTGCAGGTCTTGCAAACAACA

AGTCTAGAGGCGTCTTTGTCCGCTCGCCGTCGGGGCGCATCCTTTTTGCCGGTTGGCCAGCCGCCGGCGC

GACGTGATAGCGGTCACGACCCCACTGGCGATGGGCCTGAGACCCAAAAGCCCCGAATGAGCGACTGGAG

CTCCCCGCTTGAGCATCGTCAGTTCGACGACAAGGCGCATCGCATGATGTCAGCCTTGCCGGCCAAAGTT

GGCCACGCGCCGAGCATCTACCATGCCCAGACTTGCATCATAACGCCCATTCATGACATCGATACTGACA

ATGTGTGCCTACAGACACT

>YH6

GCAGCCGGAGAATCCTTTACACGAGCAAAAGGATACGCCTCTCCCAGCGTTGGCCCGCCA

GGCATTGCAGCTGAGCCGGTTAATGCCTCTCACGGCCTGCGGCGGCGGGCGCAACAAAGC

TGGGGAAGCGGCCCCGTGTTTTGAGGAATCATTGCCTCGGGGTCTCTCCGAGTCTGCCCC

GGACTGAGATTTAGGCGGGCTGCTGCAGCAGGTTGCGGCGACGGCAAGCACTGGGGCTTG

GCGGGGGTCAAACCACCGCCTGCCCCGCGGATGCTGTGTTGGGTGTTGGGTTGGCGGTCG

TTGTACGTACCTGCTCAGCTCTGCTCGGCTGGGCTTCTGGTTATTGCCAGTAGCCGGGGC

TGCTGGCTGACAGGATTCGCACACGACTCGGTTCTGCAAGGAACCCCACCCCACCTGGCT

GGTGCAGCGACTCCGAGGCCTCGCCTTGCCTCCAGCTGCAGGGTTCAACGCGGCATCTCG

GGTATTGCAGGTCTTGCAAACAACAAGTCTAGAGGCGTCTTTGTCCGCTCGCCGTCGGGG

CGCATCCTTTTTGCCGGTTGGCCAGCCGCCGGCGCGACGTGATAGCGGTCACGACCCCAC

TGGCGATGGGCCTGAGACCCAAAAGCCCCGAATGAGCGACTGGAGCTCCCCGCTTGAGCA

TCGTCAGTTCGACGACAAGGCGCATCGCATGATGTCAGCCTTGCCGGCCAAAGTTGGCCA

CGCGCCGAGCATCTACCATGCCCAGACTTGCATCATAACGCCCATTCATGACATCGATAC

TGACAATGTGTGCCTACAGACACTCAA

>YH7

GCAGCCGGAGAATCCTTTACACGAGCAAAAGGATACGCCTCTCCCAGCGTTGGCCCGCCA

GGCATTGCAGCTGAGCCGGTTAATGCCTCTCACGGCCTGCGGCGGCGGGCGCAACAAAGC

TGGGGAAGCGGCCCCGTGTTTTGAGGAATCATTGCCTCGGGGTCTCTCCGAGTCTGCCCC

GGACTGAGATTTAGGCGGGCTGCTGCAGCAGGTTGCGGCGACGGCAAGCACTGGGGCTTG

GCGGGGGTCAAACCACCGCCTGCCCCGCGGATGCTGTGTTGGGTGTTGGGTTGGCGGTCG

TTGTACGTACCTGCTCAGCTCTGCTCGGCTGGGCTTCTGGTTATTGCCAGTAGCCGGGGC

TGCTGGCTGACAGGATTCGCACACGACTCGGTTCTGCAAGGAACCCCACCCCACCTGGCT

GGTGCAGCGACTCCGAGGCCTCGCCTTGCCTCCAGCTGCAGGGTTCAACGCGGCATCTCG

GGTATTGCAGGTCTTGCAAACAACAAGTCTAGAGGCGTCTTTGTCCGCTCGCCGTCGGGG

CGCATCCTTTTTGCCGGTTGGCCAGCCGCCGGCGCGACGTGATAGCGGTCACGACCCCAC

TGGCGATGGGCCTGAGACCCAAAAGCCCCGAATGAGCGACTGGAGCTCCCCGCTTGAGCA

TCGTCAGTTCGACGACAAGGCGCATCGCATGATGTCAGCCTTGCCGGCCAAAGTTGGCCA

CGCGCCGAGCATCTACCATGCCCAGACTTGCATCATAACGCCCATTCATGACATCGATAC

TGACAATGTGTGCCTACAGACACTCAA

>YM2

GCAGCCGGAGAATCCTTTACACGAGCAAAAGGATACGCCTCTCCCAGCGTTGGCCCGCCA

GGCATTGCAGCTGAGCCGGTTAATGCCTCTCACGGCCTGCGGCGGCGGGCGCAACAAAGC

TGGGGAAGCGGCCCCGTGTTTTGAGGAATCATTGCCTCGGGGTCTCTCCGAGTCTGCCCC

GGACTGAGATTTAGGCGGGCTGCTGCAGCAGGTTGCGGCGACGGCAAGCACTGGGGCTTG

GCGGGGGTCAAACCACCGCCTGCCCCGCGGATGCTGTGTTGGGTGTTGGGTTGGCGGTCG

TTGTACGTACCTGCTCAGCTCTGCTCGGCTGGGCTTCTGGTTATTGCCAGTAGCCGGGGC

TGCTGGCTGACAGGATTCGCACACGACTCGGTTCTGCAAGGAACCCCACCCCACCTGGCT

GGTGCAGCGACTCCGAGGCCTCGCCTTGCCTCCAGCTGCAGGGTTCAACGCGGCATCTCG

GGTATTGCAGGTCTTGCAAACAACAAGTCTAGAGGCGTCTTTGTCCGCTCGCCGTCGGGG

CGCATCCTTTTTGCCGGTTGGCCAGCCGCCGGCGCGACGTGATAGCGGTCACGACCCCAC

TGGCGATGGGCCTGAGACCCAAAAGCCCCGAATGAGCGACTGGAGCTCCCCGCTTGAGCA

TCGTCAGTTCGACGACAAGGCGCATCGCATGATGTCAGCCTTGCCGGCCAAAGTTGGCCA

CGCGCCGAGCATCTACCATGCCCAGACTTGCATCATAACGCCCATTCATGACATCGATAC

TGACAATGTGTGCCTACAGACACTCAA

>YM7

GCAGCCGGAGAATCCTTTACACGAGCAAAAGGATACGCCTCTCCCAGCGTTGGCCCGCCA

GGCATTGCAGCTGAGCCGGTTAATGCCTCTCACGGCCTGCGGCGGCGGGCGCAACAAAGC

TGGGGAAGCGGCCCCGTGTTTTGAGGAATCATTGCCTCGGGGTCTCTCCGAGTCTGCCCC

GGACTGAGATTTAGGCGGGCTGCTGCAGCAGGTTGCGGCGACGGCAAGCACTGGGGCTTG

GCGGGGGTCAAACCACCGCCTGCCCCGCGGATGCTGTGTTGGGTGTTGGGTTGGCGGTCG

TTGTACGTACCTGCTCAGCTCTGCTCGGCTGGGCTTCTGGTTATTGCCAGTAGCCGGGGC

TGCTGGCTGACAGGATTCGCACACGACTCGGTTCTGCAAGGAACCCCACCCCACCTGGCT

GGTGCAGCGACTCCGAGGCCTCGCCTTGCCTCCAGCTGCAGGGTTCAACGCGGCATCTCG

GGTATTGCAGGTCTTGCAAACAACAAGTCTAGAGGCGTCTTTGTCCGCTCGCCGTCGGGG

CGCATCCTTTTTGCCGGTTGGCCAGCCGCCGGCGCGACGTGATAGCGGTCACGACCCCAC

TGGCGATGGGCCTGAGACCCAAAAGCCCCGAATGAGCGACTGGAGCTCCCCGCTTGAGCA

TCGTCAGTTCGACGACAAGGCGCATCGCATGATGTCAGCCTTGCCGGCCAAAGTTGGCCA

CGCGCCGAGCATCTACCATGCCCAGACTTGCATCATAACGCCCATTCATGACATCGATAC

TGACAATGTGTGCCTACAGACACTCAA

>ZH6

TCACATCCGCCGGGCGTTGGCTCGGTTCGCCGCCCGCACAATCGCCCGCACTGCTGCAGCCGGAGAATCC

TTTACACGAGCAAAAGGATACGCCTCTCCCAGCGTTGGCCCGCCAGGCATTGCAGCTGAGCCGGTTAATG

CCTCTCACGGCCTGCGGCGGCGGGCGCAACAAAGCTGGGGAAGCGGCCCCGTGTTTTGAGGAATCATTGC

CTCGGGGTCTCTCCGAGTCTGCCCCGGACTGAGATTTAGGCGGGCTGCTGCAGCAGGTTGCGGCGACGGC

AAGCACTGGGGCTTGGCGGGGGTCAAACCACCGCCTGCCCCGCGGATGCTGTGTTGGGTGTTGGGTTGGC

GGTCGTTGTACGTACCTGCTCAGCTCTGCTCGGCTGGGCTTCTGGTTATTGCCAGTAGCCGGGGCTGCTG

GCTGACAGGATTCGCACACGACTCGGTTCTGCAAGGAACCCCACCCCACCTGGCTGGTGCAGCGACTCCG

AGGCCTCGCCTTGCCTCCAGCTGCAGGGTTCAACGCGGCATCTCGGGTATTGCAGGTCTTGCAAACAACA

AGTCTAGAGGCGTCTTTGTCCGCTCGCCGTCGGGGCGCATCCTTTTTGCCGGTTGGCCAGCCGCCGGCGC

GACGTGATAGCGGTCACGACCCCACTGGCGATGGGCCTGAGACCCAAAAGCCCCGAATGAGCGACTGGAG

CTCCCCGCTTGAGCATCGTCAGTTCGACGACAAGGCGCATCGCATGATGTCAGCCTTGCCGGCCAAAGTT

GGCCACGCGCCGAGCATCTACCATGCCCAGACTTGCATCATAACGCCCATTCATGACATCGATACTGACA

ATGTGTGCCTACAGACACT

>BM6

TGTCACACCCGCCGGGCGCTGGCTCGGTTCGCCGCCCGCAAAACCGCCCGCACTGCTGCA

GCCGGAGAATCCTTTACACGAGCAAAAGGATACGCCTCTCCCAGCGTTGGCCCGCCACAC

GGTGCAGCTGAGCCGGTTAATGCCTCTCACGACCTGCGGCGGCGGGAGCAACAAAGCTGG

GGAAGCGGCCCCGTGTTTTGAGGAATTATTGCCTCGGGGTCTCTCCGAGTCTGCCCCGGA

CTGAGATTTAGGCGGGCTGCTGCAGCAGGTTGCGGCGACGGCAAGCACTGGGGCTTGGCG

GGGGTCAAACCACCGCCTGCCCCGCGGATGCTGTATTGGGTGTTGGGTTGGCGGTTGTTG

TACGTACCTGCTCAGCTCTGCTCGGCTGGGCTTCTGGTTATTGCCAGTAGCCGGGGCTGC

TGGCTGACAGGATTCGCACACGACTCGGTTCTGCAAGGAACCCCACCCCACCTGGCCGGT

GCAGCGACTCCGAGGCCTCGCCTTGCCTCCAGCTGCAGGGTTCAACGCGGCATCTCGGGT

ATTGCAGGTCTTGCAAACAAAAGTCTAGAGGCGTCGTTGTCCGCTCGCCGTCGGGGCGCA

TCCTTTTTGCCGGTTGGCCAGCCGCCGGCGCGACGTGATAGCGGTCACTACCCCACTGGC

GATGGGCCCGAGACCCAAAAGCCCCGAATGAGCGACTGGAGCTCCCCGCTTGAGCATCGT

CAGTTCGACGACAAGGCGCATCGCATGATGTCAGCCTTGCCGGCCAAAGTTGGCCACGCG

CCGAGCATTTACCATGTCCAGACTTGCATCATAACGCCACATTCACAATATCGATACTGA

CAATGTGCGTCTATAGACACTCAA

>F8

TGTCACACCCGCCGGGCGCTGGCTCGGTTCGCCGCCCGCAAAACCGCCCGCACTGCTGCA

GCCGGAGAATCCTTTACACGAGCAAAAGGATACGCCTCTCCCAGCGTTGGCCCGCCACAC

GGTGCAGCTGAGCCGGTTAATGCCTCTCACGACCTGCGGCGGCGGGAGCAACAAAGCTGG

GGAAGCGGCCCCGTGTTTTGAGGAATTATTGCCTCGGGGTCTCTCCGAGTCTGCCCCGGA

CTGAGATTTAGGCGGGCTGCTGCAGCAGGTTGCGGCGACGGCAAGCACTGGGGCTTGGCG

GGGGTCAAACCACCGCCTGCCCCGCGGATGCTGTATTGGGTGTTGGGTTGGCGGTTGTTG

TACGTACCTGCTCAGCTCTGCTCGGCTGGGCTTCTGGTTATTGCCAGTAGCCGGGGCTGC

TGGCTGACAGGATTCGCACACGACTCGGTTCTGCAAGGAACCCCACCCCACCTGGCCGGT

GCAGCGACTCCGAGGCCTCGCCTTGCCTCCAGCTGCAGGGTTCAACGCGGCATCTCGGGT

ATTGCAGGTCTTGCAAACAAAAGTCTAGAGGCGTCGTTGTCCGCTCGCCGTCGGGGCGCA

TCCTTTTTGCCGGTTGGCCAGCCGCCGGCGCGACGTGATAGCGGTCACTACCCCACTGGC

GATGGGCCCGAGACCCAAAAGCCCCGAATGAGCGACTGGAGCTCCCCGCTTGAGCATCGT

CAGTTCGACGACAAGGCGCATCGCATGATGTCAGCCTTGCCGGCCAAAGTTGGCCACGCG

CCGAGCATTTACCATGTCCAGACTTGCATCATAACGCCACATTCACAATATCGATACTGA

CAATGTGCGTCTATAGACACTCAA

>GX3

TGTCACACCCGCCGGGCGCTGGCTCGGTTCGCCGCCCGCAAAACCGCCCGCACTGCTGCA

GCCGGAGAATCCTTTACACGAGCAAAAGGATACGCCTCTCCCAGCGTTGGCCCGCCACAC

GGTGCAGCTGAGCCGGTTAATGCCTCTCACGACCTGCGGCGGCGGGAGCAACAAAGCTGG

GGAAGCGGCCCCGTGTTTTGAGGAATTATTGCCTCGGGGTCTCTCCGAGTCTGCCCCGGA

CTGAGATTTAGGCGGGCTGCTGCAGCAGGTTGCGGCGACGGCAAGCACTGGGGCTTGGCG

GGGGTCAAACCACCGCCTGCCCCGCGGATGCTGTATTGGGTGTTGGGTTGGCGGTTGTTG

TACGTACCTGCTCAGCTCTGCTCGGCTGGGCTTCTGGTTATTGCCAGTAGCCGGGGCTGC

TGGCTGACAGGATTCGCACACGACTCGGTTCTGCAAGGAACCCCACCCCACCTGGCCGGT

GCAGCGACTCCGAGGCCTCGCCTTGCCTCCAGCTGCAGGGTTCAACGCGGCATCTCGGGT

ATTGCAGGTCTTGCAAACAAAAGTCTAGAGGCGTCGTTGTCCGCTCGCCGTCGGGGCGCA

TCCTTTTTGCCGGTTGGCCAGCCGCCGGCGCGACGTGATAGCGGTCACTACCCCACTGGC

GATGGGCCCGAGACCCAAAAGCCCCGAATGAGCGACTGGAGCTCCCCGCTTGAGCATCGT

CAGTTCGACGACAAGGCGCATCGCATGATGTCAGCCTTGCCGGCCAAAGTTGGCCACGCG

CCGAGCATTTACCATGTCCAGACTTGCATCATAACGCCACATTCACAATATCGATACTGA

CAATGTGCGTCTATAGACACTCAA

>JS1

TGTCACACCCGCCGGGCGCTGGCTCGGTTCGCCGCCCGCAAAACCGCCCGCACTGCTGCA

GCCGGAGAATCCTTTACACGAGCAAAAGGATACGCCTCTCCCAGCGTTGGCCCGCCACAC

GGTGCAGCTGAGCCGGTTAATGCCTCTCACGACCTGCGGCGGCGGGAGCAACAAAGCTGG

GGAAGCGGCCCCGTGTTTTGAGGAATTATTGCCTCGGGGTCTCTCCGAGTCTGCCCCGGA

CTGAGATTTAGGCGGGCTGCTGCAGCAGGTTGCGGCGACGGCAAGCACTGGGGCTTGGCG

GGGGTCAAACCACCGCCTGCCCCGCGGATGCTGTATTGGGTGTTGGGTTGGCGGTTGTTG

TACGTACCTGCTCAGCTCTGCTCGGCTGGGCTTCTGGTTATTGCCAGTAGCCGGGGCTGC

TGGCTGACAGGATTCGCACACGACTCGGTTCTGCAAGGAACCCCACCCCACCTGGCCGGT

GCAGCGACTCCGAGGCCTCGCCTTGCCTCCAGCTGCAGGGTTCAACGCGGCATCTCGGGT

ATTGCAGGTCTTGCAAACAAAAGTCTAGAGGCGTCGTTGTCCGCTCGCCGTCGGGGCGCA

TCCTTTTTGCCGGTTGGCCAGCCGCCGGCGCGACGTGATAGCGGTCACTACCCCACTGGC

GATGGGCCCGAGACCCAAAAGCCCCGAATGAGCGACTGGAGCTCCCCGCTTGAGCATCGT

CAGTTCGACGACAAGGCGCATCGCATGATGTCAGCCTTGCCGGCCAAAGTTGGCCACGCG

CCGAGCATTTACCATGTCCAGACTTGCATCATAACGCCACATTCACAATATCGATACTGA

CAATGTGCGTCTATAGACACTCAA

>JS5

TGTCACACCCGCCGGGCGCTGGCTCGGTTCGCCGCCCGCAAAACCGCCCGCACTGCTGCA

GCCGGAGAATCCTTTACACGAGCAAAAGGATACGCCTCTCCCAGCGTTGGCCCGCCACAC

GGTGCAGCTGAGCCGGTTAATGCCTCTCACGACCTGCGGCGGCGGGAGCAACAAAGCTGG

GGAAGCGGCCCCGTGTTTTGAGGAATTATTGCCTCGGGGTCTCTCCGAGTCTGCCCCGGA

CTGAGATTTAGGCGGGCTGCTGCAGCAGGTTGCGGCGACGGCAAGCACTGGGGCTTGGCG

GGGGTCAAACCACCGCCTGCCCCGCGGATGCTGTATTGGGTGTTGGGTTGGCGGTTGTTG

TACGTACCTGCTCAGCTCTGCTCGGCTGGGCTTCTGGTTATTGCCAGTAGCCGGGGCTGC

TGGCTGACAGGATTCGCACACGACTCGGTTCTGCAAGGAACCCCACCCCACCTGGCCGGT

GCAGCGACTCCGAGGCCTCGCCTTGCCTCCAGCTGCAGGGTTCAACGCGGCATCTCGGGT

ATTGCAGGTCTTGCAAACAAAAGTCTAGAGGCGTCGTTGTCCGCTCGCCGTCGGGGCGCA

TCCTTTTTGCCGGTTGGCCAGCCGCCGGCGCGACGTGATAGCGGTCACTACCCCACTGGC

GATGGGCCCGAGACCCAAAAGCCCCGAATGAGCGACTGGAGCTCCCCGCTTGAGCATCGT

CAGTTCGACGACAAGGCGCATCGCATGATGTCAGCCTTGCCGGCCAAAGTTGGCCACGCG

CCGAGCATTTACCATGTCCAGACTTGCATCATAACGCCACATTCACAATATCGATACTGA

CAATGTGCGTCTATAGACACTCAA

>LC2

TGTCACACCCGCCGGGCGCTGGCTCGGTTCGCCGCCCGCAAAACCGCCCGCACTGCTGCA

GCCGGAGAATCCTTTACACGAGCAAAAGGATACGCCTCTCCCAGCGTTGGCCCGCCACAC

GGTGCAGCTGAGCCGGTTAATGCCTCTCACGACCTGCGGCGGCGGGAGCAACAAAGCTGG

GGAAGCGGCCCCGTGTTTTGAGGAATTATTGCCTCGGGGTCTCTCCGAGTCTGCCCCGGA

CTGAGATTTAGGCGGGCTGCTGCAGCAGGTTGCGGCGACGGCAAGCACTGGGGCTTGGCG

GGGGTCAAACCACCGCCTGCCCCGCGGATGCTGTATTGGGTGTTGGGTTGGCGGTTGTTG

TACGTACCTGCTCAGCTCTGCTCGGCTGGGCTTCTGGTTATTGCCAGTAGCCGGGGCTGC

TGGCTGACAGGATTCGCACACGACTCGGTTCTGCAAGGAACCCCACCCCACCTGGCCGGT

GCAGCGACTCCGAGGCCTCGCCTTGCCTCCAGCTGCAGGGTTCAACGCGGCATCTCGGGT

ATTGCAGGTCTTGCAAACAAAAGTCTAGAGGCGTCGTTGTCCGCTCGCCGTCGGGGCGCA

TCCTTTTTGCCGGTTGGCCAGCCGCCGGCGCGACGTGATAGCGGTCACTACCCCACTGGC

GATGGGCCCGAGACCCAAAAGCCCCGAATGAGCGACTGGAGCTCCCCGCTTGAGCATCGT

CAGTTCGACGACAAGGCGCATCGCATGATGTCAGCCTTGCCGGCCAAAGTTGGCCACGCG

CCGAGCATTTACCATGTCCAGACTTGCATCATAACGCCACATTCACAATATCGATACTGA

CAATGTGCGTCTATAGACACTCAA

>LC6

TGTCACACCCGCCGGGCGCTGGCTCGGTTCGCCGCCCGCAAAACCGCCCGCACTGCTGCA

GCCGGAGAATCCTTTACACGAGCAAAAGGATACGCCTCTCCCAGCGTTGGCCCGCCACAC

GGTGCAGCTGAGCCGGTTAATGCCTCTCACGACCTGCGGCGGCGGGAGCAACAAAGCTGG

GGAAGCGGCCCCGTGTTTTGAGGAATTATTGCCTCGGGGTCTCTCCGAGTCTGCCCCGGA

CTGAGATTTAGGCGGGCTGCTGCAGCAGGTTGCGGCGACGGCAAGCACTGGGGCTTGGCG

GGGGTCAAACCACCGCCTGCCCCGCGGATGCTGTATTGGGTGTTGGGTTGGCGGTTGTTG

TACGTACCTGCTCAGCTCTGCTCGGCTGGGCTTCTGGTTATTGCCAGTAGCCGGGGCTGC

TGGCTGACAGGATTCGCACACGACTCGGTTCTGCAAGGAACCCCACCCCACCTGGCCGGT

GCAGCGACTCCGAGGCCTCGCCTTGCCTCCAGCTGCAGGGTTCAACGCGGCATCTCGGGT

ATTGCAGGTCTTGCAAACAAAAGTCTAGAGGCGTCGTTGTCCGCTCGCCGTCGGGGCGCA

TCCTTTTTGCCGGTTGGCCAGCCGCCGGCGCGACGTGATAGCGGTCACTACCCCACTGGC

GATGGGCCCGAGACCCAAAAGCCCCGAATGAGCGACTGGAGCTCCCCGCTTGAGCATCGT

CAGTTCGACGACAAGGCGCATCGCATGATGTCAGCCTTGCCGGCCAAAGTTGGCCACGCG

CCGAGCATTTACCATGTCCAGACTTGCATCATAACGCCACATTCACAATATCGATACTGA

CAATGTGCGTCTATAGACACTCAA

>YM4

TGTCACACCCGCCGGGCGCTGGCTCGGTTCGCCGCCCGCAAAACCGCCCGCACTGCTGCA

GCCGGAGAATCCTTTACACGAGCAAAAGGATACGCCTCTCCCAGCGTTGGCCCGCCACAC

GGTGCAGCTGAGCCGGTTAATGCCTCTCACGACCTGCGGCGGCGGGAGCAACAAAGCTGG

GGAAGCGGCCCCGTGTTTTGAGGAATTATTGCCTCGGGGTCTCTCCGAGTCTGCCCCGGA

CTGAGATTTAGGCGGGCTGCTGCAGCAGGTTGCGGCGACGGCAAGCACTGGGGCTTGGCG

GGGGTCAAACCACCGCCTGCCCCGCGGATGCTGTATTGGGTGTTGGGTTGGCGGTTGTTG

TACGTACCTGCTCAGCTCTGCTCGGCTGGGCTTCTGGTTATTGCCAGTAGCCGGGGCTGC

TGGCTGACAGGATTCGCACACGACTCGGTTCTGCAAGGAACCCCACCCCACCTGGCCGGT

GCAGCGACTCCGAGGCCTCGCCTTGCCTCCAGCTGCAGGGTTCAACGCGGCATCTCGGGT

ATTGCAGGTCTTGCAAACAAAAGTCTAGAGGCGTCGTTGTCCGCTCGCCGTCGGGGCGCA

TCCTTTTTGCCGGTTGGCCAGCCGCCGGCGCGACGTGATAGCGGTCACTACCCCACTGGC

GATGGGCCCGAGACCCAAAAGCCCCGAATGAGCGACTGGAGCTCCCCGCTTGAGCATCGT

CAGTTCGACGACAAGGCGCATCGCATGATGTCAGCCTTGCCGGCCAAAGTTGGCCACGCG

CCGAGCATTTACCATGTCCAGACTTGCATCATAACGCCACATTCACAATATCGATACTGA

CAATGTGCGTCTATAGACACTCAA

>YM5

TGTCACACCCGCCGGGCGCTGGCTCGGTTCGCCGCCCGCAAAACCGCCCGCACTGCTGCA

GCCGGAGAATCCTTTACACGAGCAAAAGGATACGCCTCTCCCAGCGTTGGCCCGCCACAC

GGTGCAGCTGAGCCGGTTAATGCCTCTCACGACCTGCGGCGGCGGGAGCAACAAAGCTGG

GGAAGCGGCCCCGTGTTTTGAGGAATTATTGCCTCGGGGTCTCTCCGAGTCTGCCCCGGA

CTGAGATTTAGGCGGGCTGCTGCAGCAGGTTGCGGCGACGGCAAGCACTGGGGCTTGGCG

GGGGTCAAACCACCGCCTGCCCCGCGGATGCTGTATTGGGTGTTGGGTTGGCGGTTGTTG

TACGTACCTGCTCAGCTCTGCTCGGCTGGGCTTCTGGTTATTGCCAGTAGCCGGGGCTGC

TGGCTGACAGGATTCGCACACGACTCGGTTCTGCAAGGAACCCCACCCCACCTGGCCGGT

GCAGCGACTCCGAGGCCTCGCCTTGCCTCCAGCTGCAGGGTTCAACGCGGCATCTCGGGT

ATTGCAGGTCTTGCAAACAAAAGTCTAGAGGCGTCGTTGTCCGCTCGCCGTCGGGGCGCA

TCCTTTTTGCCGGTTGGCCAGCCGCCGGCGCGACGTGATAGCGGTCACTACCCCACTGGC

GATGGGCCCGAGACCCAAAAGCCCCGAATGAGCGACTGGAGCTCCCCGCTTGAGCATCGT

CAGTTCGACGACAAGGCGCATCGCATGATGTCAGCCTTGCCGGCCAAAGTTGGCCACGCG

CCGAGCATTTACCATGTCCAGACTTGCATCATAACGCCACATTCACAATATCGATACTGA

CAATGTGCGTCTATAGACACTCAA

>ZH3

TGTCACACCCGCCGGGCGCTGGCTCGGTTCGCCGCCCGCAAAACCGCCCGCACTGCTGCA

GCCGGAGAATCCTTTACACGAGCAAAAGGATACGCCTCTCCCAGCGTTGGCCCGCCACAC

GGTGCAGCTGAGCCGGTTAATGCCTCTCACGACCTGCGGCGGCGGGAGCAACAAAGCTGG

GGAAGCGGCCCCGTGTTTTGAGGAATTATTGCCTCGGGGTCTCTCCGAGTCTGCCCCGGA

CTGAGATTTAGGCGGGCTGCTGCAGCAGGTTGCGGCGACGGCAAGCACTGGGGCTTGGCG

GGGGTCAAACCACCGCCTGCCCCGCGGATGCTGTATTGGGTGTTGGGTTGGCGGTTGTTG

TACGTACCTGCTCAGCTCTGCTCGGCTGGGCTTCTGGTTATTGCCAGTAGCCGGGGCTGC

TGGCTGACAGGATTCGCACACGACTCGGTTCTGCAAGGAACCCCACCCCACCTGGCCGGT

GCAGCGACTCCGAGGCCTCGCCTTGCCTCCAGCTGCAGGGTTCAACGCGGCATCTCGGGT

ATTGCAGGTCTTGCAAACAAAAGTCTAGAGGCGTCGTTGTCCGCTCGCCGTCGGGGCGCA

TCCTTTTTGCCGGTTGGCCAGCCGCCGGCGCGACGTGATAGCGGTCACTACCCCACTGGC

GATGGGCCCGAGACCCAAAAGCCCCGAATGAGCGACTGGAGCTCCCCGCTTGAGCATCGT

CAGTTCGACGACAAGGCGCATCGCATGATGTCAGCCTTGCCGGCCAAAGTTGGCCACGCG

CCGAGCATTTACCATGTCCAGACTTGCATCATAACGCCACATTCACAATATCGATACTGA

CAATGTGCGTCTATAGACACTCAA
